# Supplementary material for: Neonatal expression of human FMRP isoform corrects cortical deficits and improves behavior in a mouse model of fragile X syndrome
Source: Mol Ther Nucleic Acids. 2026 Jun 12;37(3):102981. doi: 10.1016/j.omtn.2026.102981 (PMC13320503; doi:10.1016/j.omtn.2026.102981)
Supplement: Document S1. Figures S1–S15 [file mmc1.pdf]

OMTN, Volume *37*

Supplemental information

Neonatal expression of human FMRP isoform corrects cortical deficits and improves behavior in a mouse model of fragile X syndrome

Anna O. Norman, Courtney Scaramella, Dominik Biezonski, Ralph D. Hector, Alexandra Varallo, Aarushi Sahni, Nadia Farooq, Suzanne R. Burstein, Juliana Benito, Khaleel A. Razak, Jim Selfridge, Stuart Cobb, and Iryna M. Ethell

Supplemental Figures

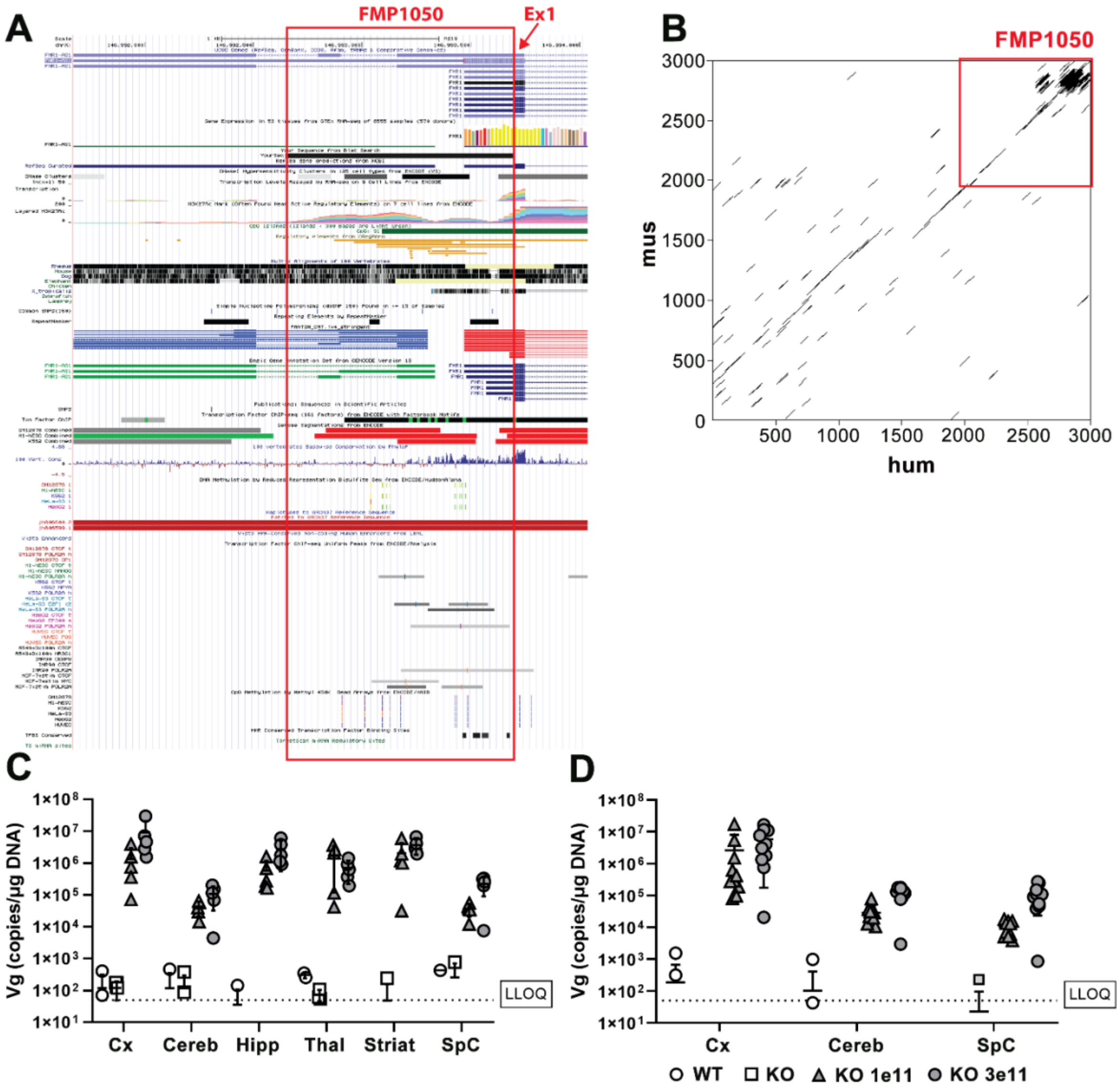

Fig. S1. Computational analysis of the human *FMR1* promoter region and analysis of transduction levels in the tissue of KO mice injected with AAV9-NG276 harvested after EEG and behavioral testing

(A) The human *FMR1* promoter region in the UCSC Genome Browser. The 1050 bp fragment (red box) contains key genetic regulatory elements needed for *FMR1* transgene expression, including CpG island and H3K27Ac marks. (B) Dotplot showing high levels of conservation between human and mouse sequences in the *FMR1* promoter region upstream of the coding exons. Generated by EMBOSS Dotmatcher. (C)

Vector genome (expressed per µg DNA) analyzed in the tissue of WT, KO, and KO injected with AAV9-NG276 at either 1e11 (low-dose) or 3e11 (high-dose) vg/animal (n=5 mice per group) after the EEG recording and (D) behavior testing. The highest dose-dependent levels of transduction were seen across the cortex, and lowest in the cerebellum and spinal cord. Data determined by qPCR analysis of tissues following test article administration. Circles represent individual animals. Error bars are ± SEM. Cx, cortex; Cereb, cerebellum; Hipp, hippocampus; Thal, thalamus; Striat, striatum; SpC, spinal cord; LLOQ, lower limit of quantification.

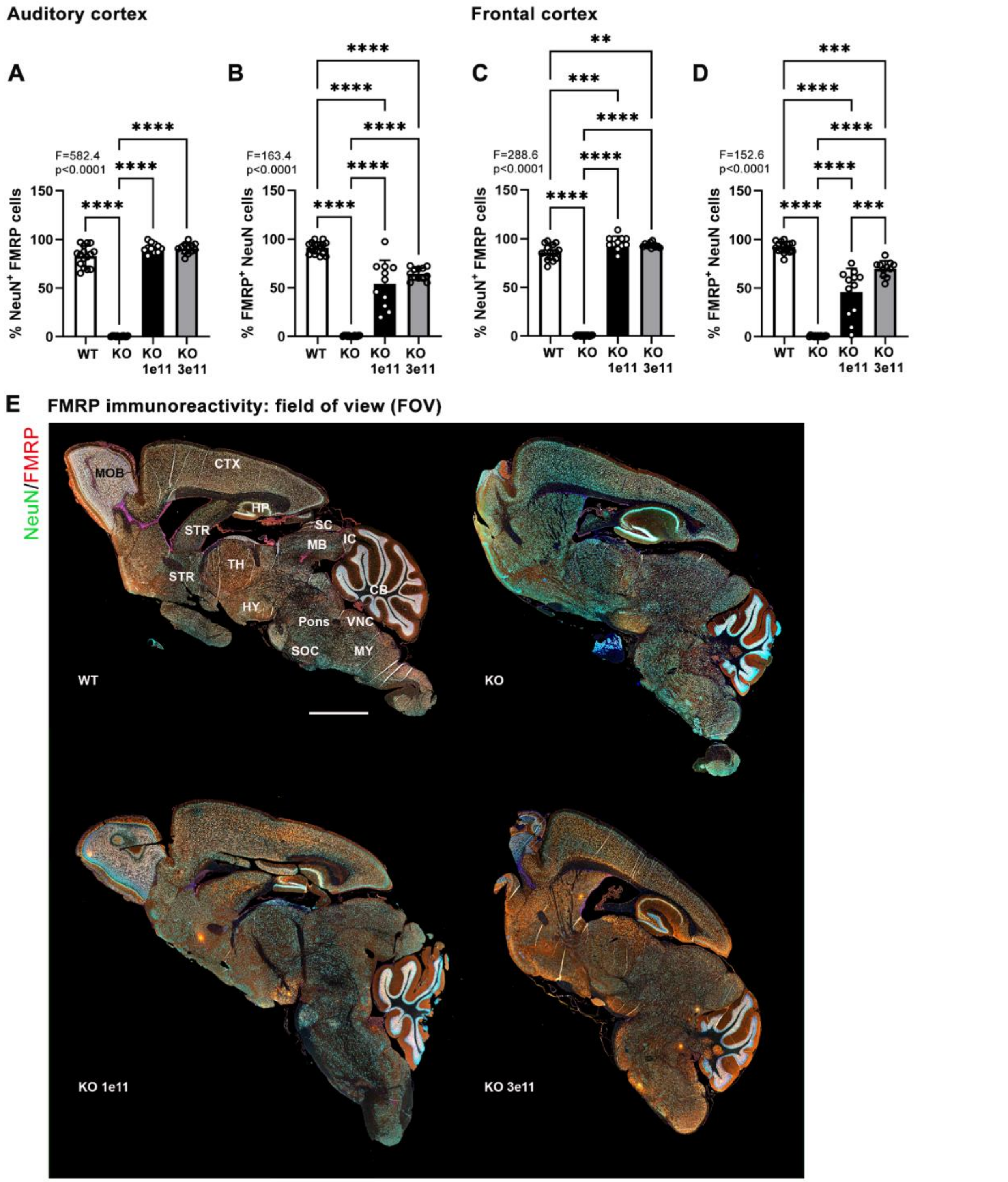

Fig. S2. FMRP levels in the AuC and FC of P28-P30 WT and KO mice ICV injected with vehicle (control) or AAV9-NG276 at a dose of 1e11 vg (1e11) or 3e11 vg (3e11) per mouse.

(A-D) Quantitative analysis of % NeuN+ FMRP cells (A, C) and % FMRP+ NeuN cells (B, D) in the AuC (A-B) and FC (C-D, n=10-15 images from 3-5 mice per group). (E) Representative images showing FMRP (red) and NeuN (green) immunoreactivity in P28-P30 vehicle-injected WT, vehicle-injected KO, and KO injected with AAV9-NG276 at low and high doses, scale bar 2 mm. CB, cerebellum; CTX, cortex; HY, hypothalamus; HP, hippocampus; IC, inferior colliculus; MB, midbrain; MOB, main olfactory bulb; MY, medulla; SC, superior colliculus; STR, striatum; TH, thalamus; VNC, vestibular nuclei. Statistical analysis was done using one-way ANOVA with a Tukey's post-hoc test: *, $p < 0.05$**, $p < 0.01$; ***, $p < 0.001$; ****, $p < 0.0001$. All graphs represent average values, and the error bars indicate SD. p values reported in the figure represent ANOVA main effects. FMRP immunoreactivity was significantly increased in the AuC and FC of KO 3e11 group.

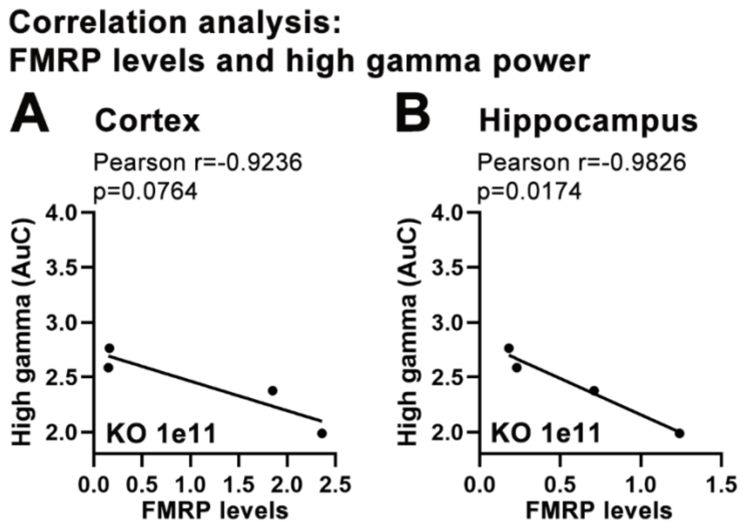

Fig. S3. Correlation between FMRP levels and high gamma power in the AuC of low-dose AAV9-NG276 mice

(A, B) Graphs show correlation between FMRP levels in cortex (A) and hippocampus (B) and high gamma power in AuC of P28-P30 KO injected with AAV9-NG276 at 1e11 (low-dose) vg/animal (n=4 mice per group). Statistical analysis was done using Pearson correlation. Negative correlation was observed between FMRP protein levels and high gamma power in the AuC of low-dose AAV9-NG276 mice.

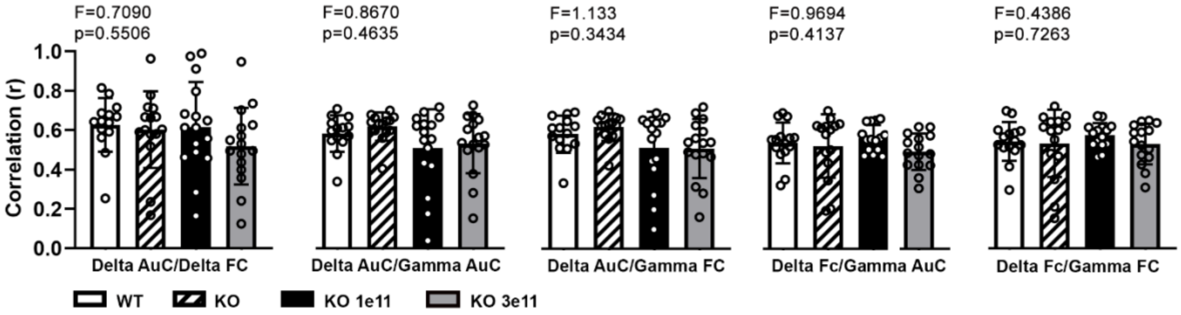

Fig. S4. No difference in delta/high gamma frequency power coupling was detected across the regions in the P28-P30 mice.

Graphs show Pearson's correlation (r) for delta/high gamma frequency power coupling within or across the regions. Values were grouped by frequency bands and analyzed with one-way ANOVA with Tukey's post hoc test. All graphs represent average values, and the error bars indicate SD. p values reported in the figure represent ANOVA main effects. No differences were detected in delta/gamma frequency power coupling across the regions.

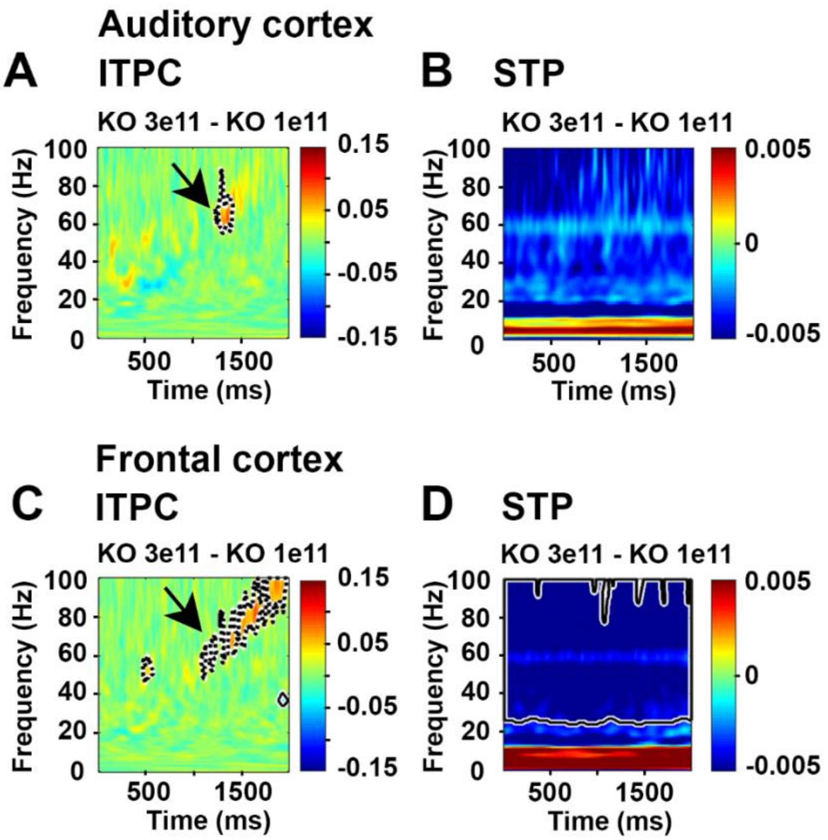

Fig. S5. Phase-locking to "up" chirp stimuli was significantly improved in high-gamma 60-100 Hz range in the P28-P30 AuC and FC of KO 3e11 compared to KO 1e11 mice.

Graphs show comparisons of ITPC (A, C) and STP (B, D) between KO 1e11 (n=16) and KO 3e11 (n=16) in the AuC (A, B) and FC (C, D) of mice. Blue areas indicate a decrease, green indicates no difference, and orange/red indicates an increase. Significant clusters ($p < 0.025$) are highlighted by bold-lined contours (the solid line depicts a significant decrease and the dotted line depicts a significant increase). Phase-locking to "up" chirp stimuli was higher in the AuC and FC of KO 3e11 compared to KO 1e11 mice.

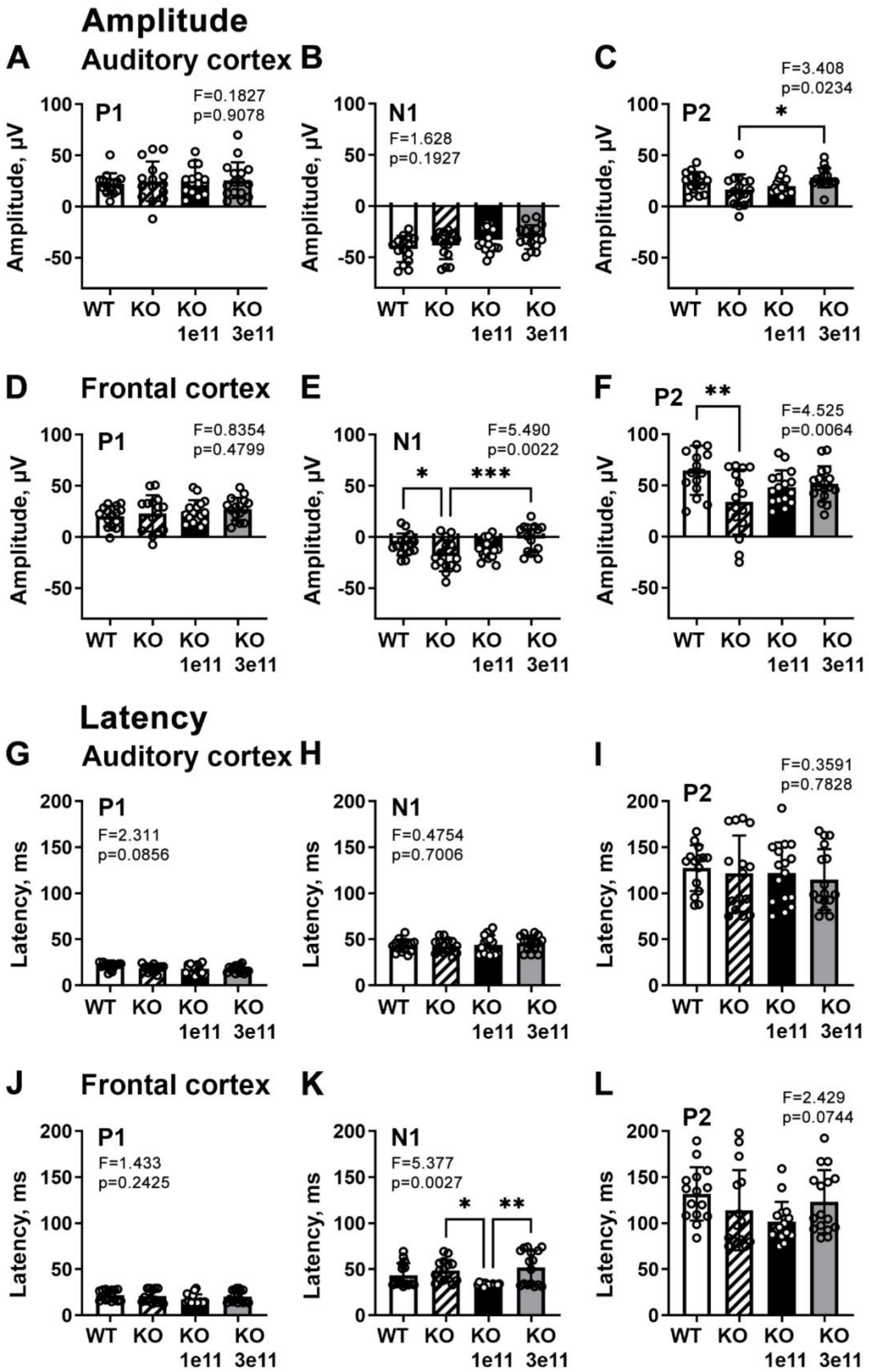

Amplitude
A
Auditory cortex
B
C
P1
F=0.1827
p=0.9078
N1
F=1.628
p=0.1927
P2
F=3.408
p=0.0234
*
D
Frontal cortex
E
F
P1
F=0.8354
p=0.4799
N1
F=5.490
p=0.0022
*
***
P2
**
F=4.525
p=0.0064
Amplitude, µV
WT
KO
KO 1e11
KO 3e11
Latency
G
Auditory cortex
H
I
P1
F=2.311
p=0.0856
N1
F=0.4754
p=0.7006
P2
F=0.3591
p=0.7828
J
Frontal cortex
K
L
P1
F=1.433
p=0.2425
N1
F=5.377
p=0.0027
*
**
P2
F=2.429
p=0.0744
Latency, ms

Fig. S6. N1 amplitude of auditory ERP was enhanced in the FC of P28-30 KO and was significantly reduced to WT levels in KO 3e11 mice.

(A-L) Auditory ERP amplitudes (A-F) and latencies (G-L) in AuC (A-C, G-I) and FC (D-F, J-L) of vehicle-injected WT (n=15), vehicle-injected KO (n=15), and KO injected with AAV9-NG276 at either 1e11 vg (n=16) or 3e11 vg/animal (n=16). Grand average ERPs obtained from P28-P30 mice in response to 100-ms broadband noise presented at 4-Hz repetition rate. P1, N1, and P2 were defined as maximum or minimum voltage deflections within 0–30 ms, 30–80 ms, or 80–150 ms, respectively. Statistical analysis was performed with a one-way ANOVA and Tukey's post hoc test. $^*p < 0.05$, $^{**} < 0.01$. All graphs represent average values and the error bars indicate SD. p values reported in the figure represent ANOVA main effects. N1 amplitude was elevated in the FC of KO compared to WT mice, and it was normalized to WT levels in KO 3e11 mice.

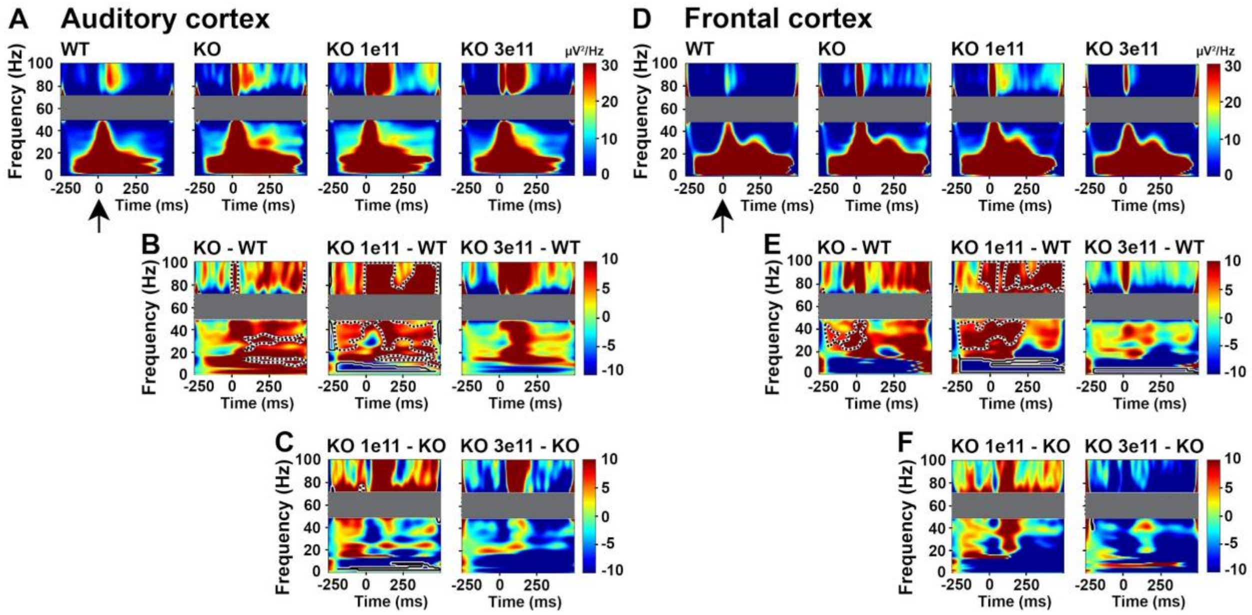

Fig. S7. The power of onset and ongoing responses to sound trains with 0.25 Hz repetition rate was impaired in the P28-P30 AuC and FC of KO mice and was improved in the high-dose group.

(A, D) Single Trial Power (STP) grand average for vehicle-injected WT (n=15), vehicle-injected KO (n=15), and KO injected with AAV9-NG276 at either 1e11 vg (n=16; low-dose) or 3e11 vg/animal (n=16; high dose) in the AuC (A-C) and FC (D-F). (B, C, E, F) Graphs show comparisons to vehicle-injected WT (B, E): KO/WT, low-dose/WT, high-dose/WT; and to vehicle-injected KO (C, F): low-dose/KO and high-dose/KO. Blue areas indicate a decrease, green indicates no difference, and orange/red indicate an increase. Significant clusters ($p < 0.025$) are highlighted by bold-lined contours (the solid line depicts a significant decrease, and the dotted line depicts a significant increase). The gray band indicates signals filtered out at ~60 Hz to prevent electrical line interference. Black arrows indicate signal presentation. The power of onset and ongoing responses to sound trains with 0.25 Hz repetition rate was enhanced in the AuC and FC of KO mice compared to WT mice, and it was improved in the high-dose, but not low-dose group.

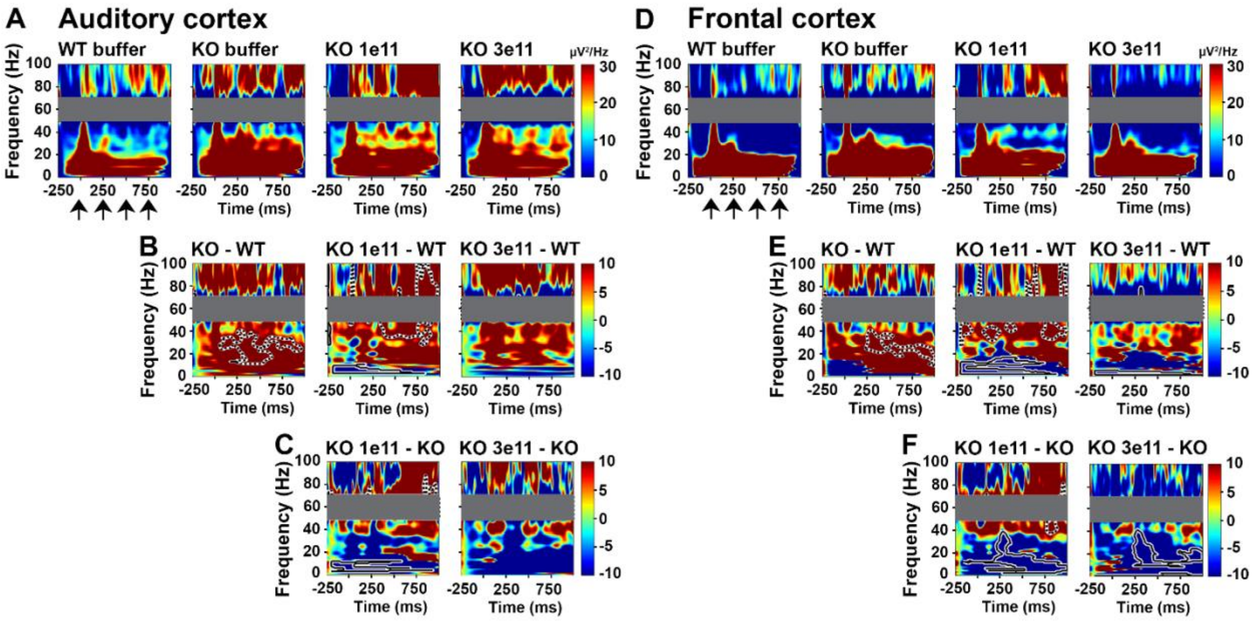

Fig. S8. The power of onset and ongoing responses to sound trains with 4 Hz repetition rate was impaired in the P28-P30 AuC and FC of KO mice and was improved in KO 3e11 but not KO 1e11 mice.

(A, D) Single Trial Power (STP) grand average for vehicle-injected WT (n=15), vehicle-injected KO (n=15), and KO injected with AAV9-NG276 at either 1e11 vg (n=16) or 3e11 vg/animal (n=16) in the AuC (A-C) and FC (D-F). (B, C, E, F) Graphs show comparisons to vehicle-injected WT (B, E): KO/WT, KO 1e11/WT, KO 3e11/WT; and to vehicle-injected KO (C, F): KO 1e11/KO and KO 3e11/KO. Blue areas indicate a decrease, green indicates no difference, and orange/red indicate an increase. Significant clusters ($p < 0.025$) are highlighted by bold-lined contours (the solid line depicts a significant decrease and the dotted line depicts a significant increase). The gray band indicates signals filtered out at ~60 Hz to prevent electrical line interference. Black arrows indicate signal presentation. The power of onset and ongoing responses to sound trains with 0.25 Hz repetition rate was enhanced in the AuC and FC of KO mice compared to WT mice, and it was improved in the KO 3e11 but not KO 1e11 group.

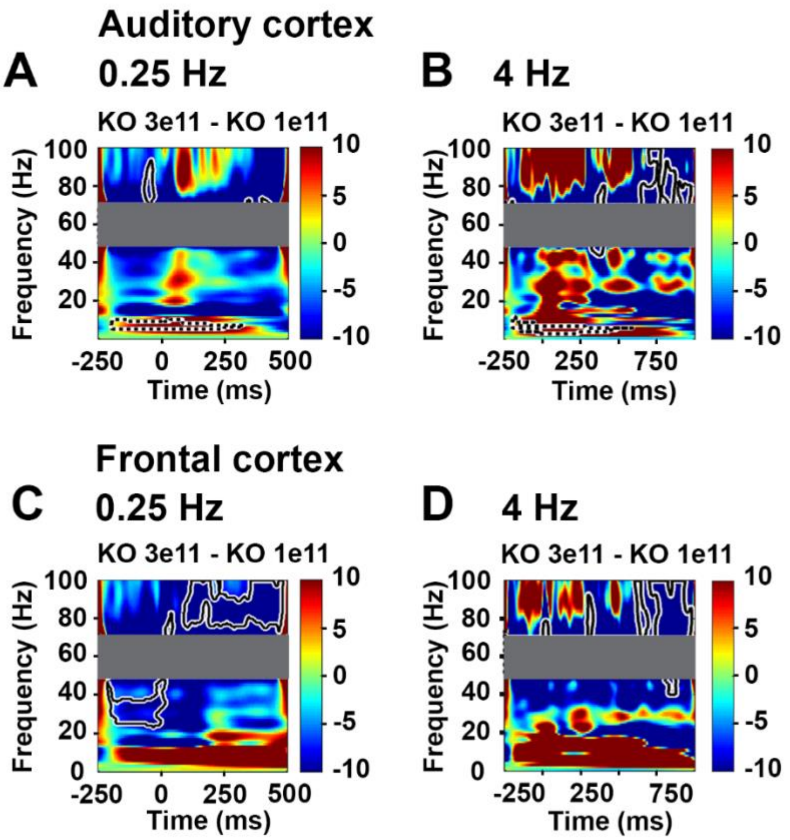

Fig. S9. The power of onset and ongoing responses to sound trains with 0.25 Hz and 4 Hz repetition rate was improved in the P28-P30 AuC and FC of KO 3e11 compared to KO 1e11 mice.

Graphs show comparisons of STP during sound trains with 0.25 Hz (A, C) and 4 Hz (B, D) repetition rate between KO 1e11 (n=16) and KO 3e11 (n=16) in the AuC (A, B) and FC (C, D) of mice. Blue areas indicate a decrease, green indicates no difference, and orange/red indicates an increase. Significant clusters ($p < 0.025$) are highlighted by bold-lined contours (the solid line depicts a significant decrease and the dotted line depicts a significant increase). The gray band indicates signals filtered out at ~60 Hz to prevent electrical line interference. The power of onset and ongoing responses to sound trains with 0.25 Hz and 4 Hz repetition rate was improved in the P28-P30 AuC and FC of KO 3e11 compared to KO 1e11 mice.

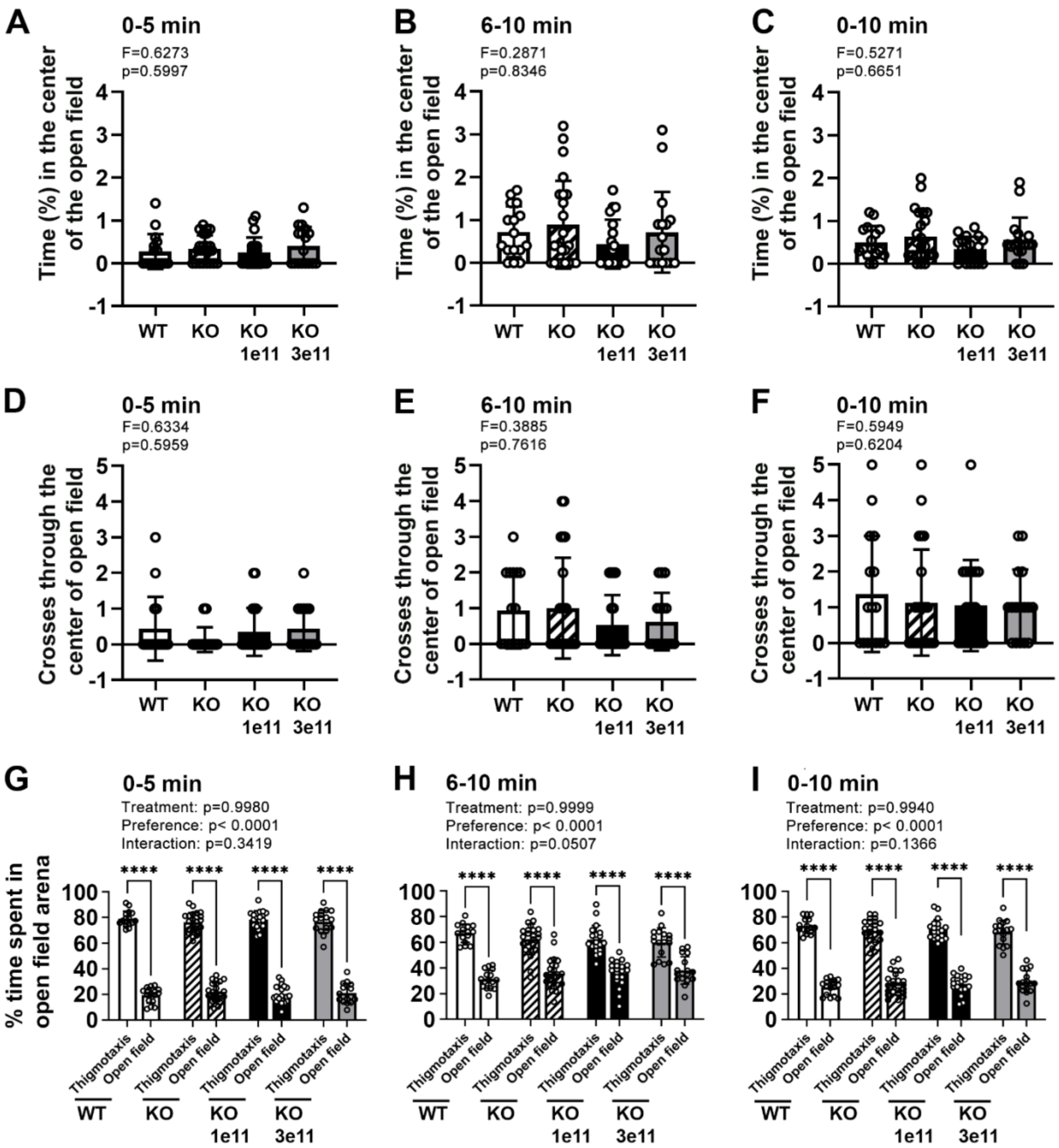

Fig. S10. No difference in exploratory behavior of P28-P30 mice was detected in the open field test.

Graphs demonstrate the performance of vehicle-injected WT (n=16), vehicle-injected KO (n=16), and KO injected with AAV9-NG276 at either 1e11 vg (n=20) or 3e11 vg/animal (n=17) in the open field test at P28-P30. Exploratory behaviors were evaluated by analyzing the % time spent in the center of the open field (A-C), crosses through the center of open field (D-F) and % time spent in thigmotaxis and open field (G-I) during first 5 min (A, D, G), second 5 min (B, E, H) and overall 10 min of testing (C, F, I). Statistical analysis was performed with a one-way (A-F) or two-way (G-I) ANOVA followed by Tukey's post hoc test.

****$p < 0.0001$. All graphs represent average values, and the error bars indicate SD. p values reported in the figure represent ANOVA main effects. No genotype and treatment difference in exploratory behavior was detected in P28-P30 mice.

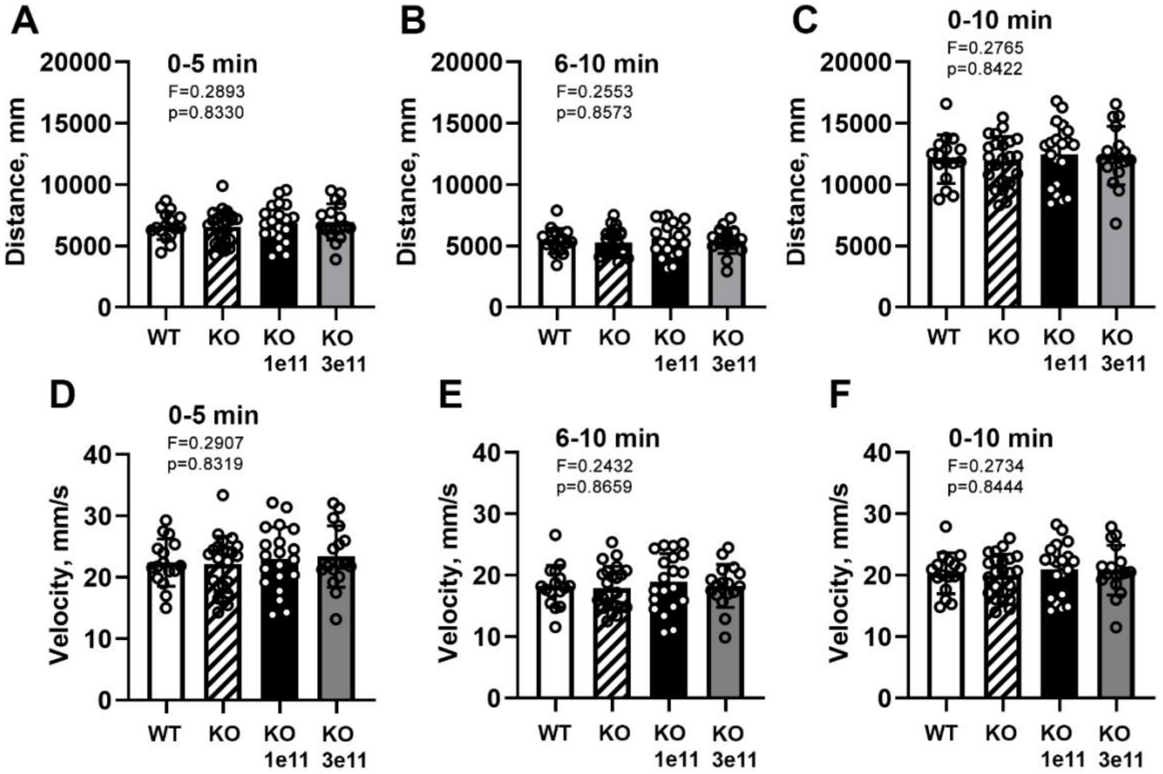

Fig. S11. No difference in locomotor activity of P28-P30 mice was detected in the open field test.

Graphs demonstrate the performance of vehicle-injected WT (n=16), vehicle-injected KO (n=16), and KO injected with AAV9-NG276 at either 1e11 vg (n=20) or 3e11 vg/animal (n=17) in the open field test at P28-P30. Locomotor activity was evaluated by analyzing overall distance traveled (A-C) and velocity (D-F) in of the open field during first 5 min (A, D), second 5 min (B, E) and overall 10 min of testing (C, F). Statistical analysis was performed with a one-way ANOVA followed by Tukey's post hoc test. All graphs represent average values, and the error bars indicate SD. p values reported in the figure represent ANOVA main effects. No genotype and treatment difference in locomotor activity was detected in P28-P30 mice.

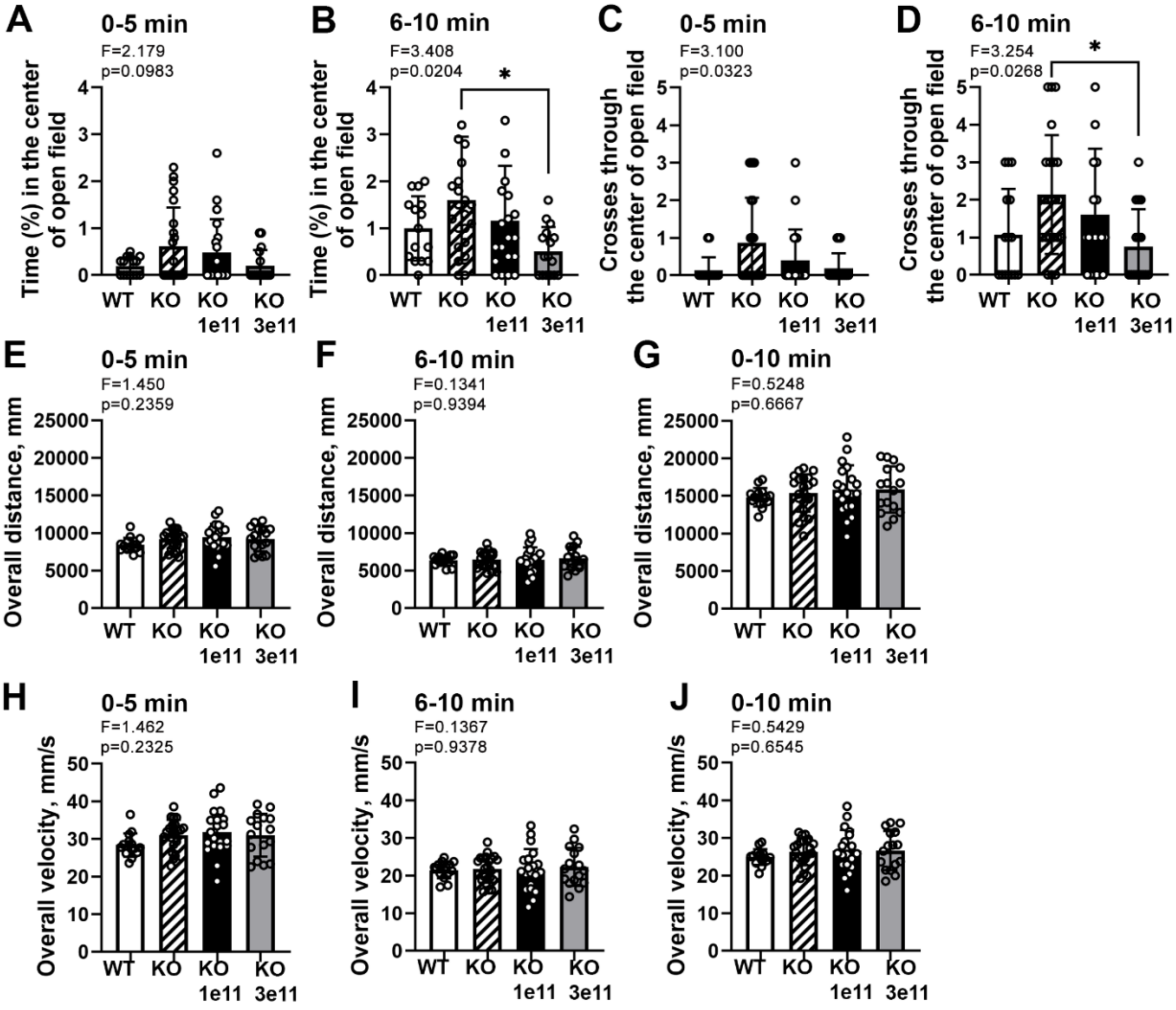

Fig. S12. No difference in locomotor activity of P60-P65 mice was detected in the open field test.

Graphs demonstrate the performance of vehicle-injected WT (n=16), vehicle-injected KO (n=16), and KO injected with AAV9-NG276 at either 1e11 vg (n=20) or 3e11 vg/animal (n=17) in the open field test at P60-P65. Exploratory behaviors were evaluated by analyzing the % time spent in the center of the open field (A, B), crosses through the center of open field (C-D) and locomotor activity was evaluated by analyzing distance (E-G) and velocity (H-J) during first 5 min (A, C, E, H), second 5 min (B, D, F, I) and overall 10 min of testing (G, J). Statistical analysis was performed with a one-way (A-F) or two-way (G-I) ANOVA followed by Tukey's post hoc test. *p < 0.05. All graphs represent average values, and the error bars indicate SD. p values reported in the figure represent ANOVA main effects. No genotype and treatment difference in locomotor activity was detected in P60-P65 mice.

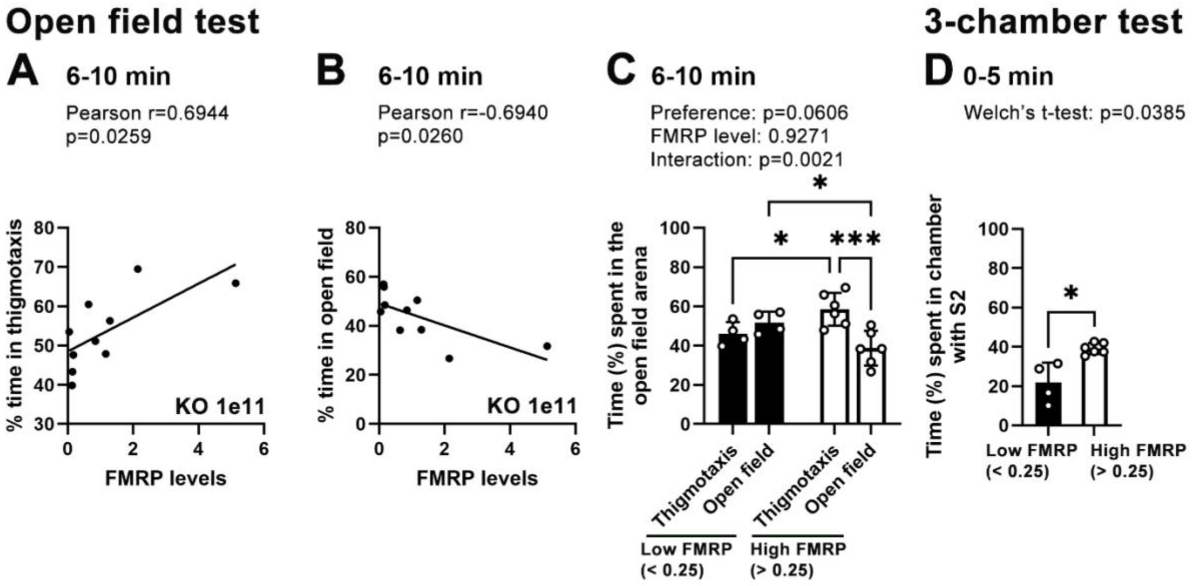

Fig. S13. Correlation between FMRP levels in cortex and exploratory behaviors of P60-P65 low-dose AAV9-NG276 mice

(A-C) Graphs show correlation between FMRP levels in cortex and exploratory behaviors, including % time spent in thigmotaxis (A), open field (B) and location preference based on FMRP levels (C) of P60-P65 KO injected with AAV9-NG276 at 1e11 (low-dose) vg/animal (n=10 mice per group). (D) Graph shows social novelty preference based on FMRP levels (low < 0.25 or high >0.25 FMRP compared to WT levels) in the cortex of low-dose KO mice. Statistical analysis was done using Pearson's correlation (A, B), two-way ANOVA followed by Fisher's LSD post-hoc test (C) or Welch's t-test (D). *p < 0.05, ***p < 0.001. Data represent mean ± standard deviation (SD). Negative correlation was observed between FMRP protein levels in cortex and exploratory behaviors of low-dose AAV9-NG276 mice.

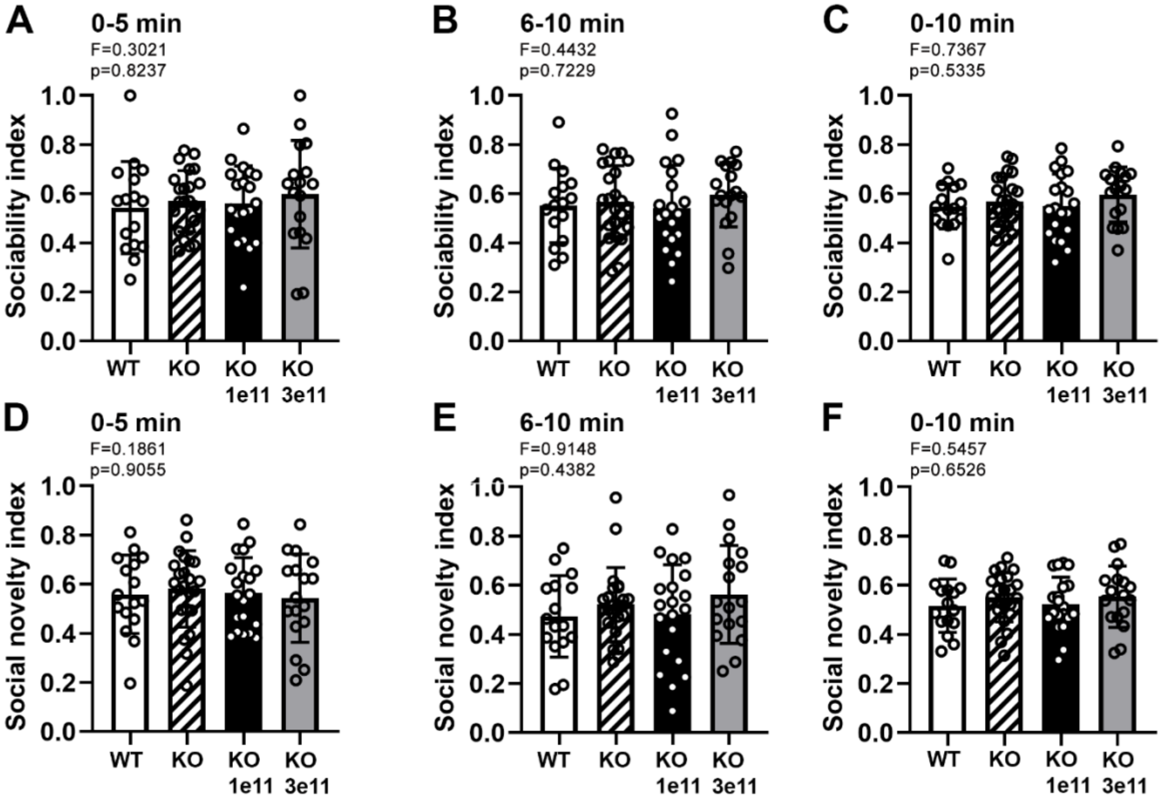

Fig. S14. No difference in sociability index of social preference index of P28-P30 mice was detected in the 3-chamber test.

Graphs demonstrate performance of P28-P30 vehicle-injected WT (n=16), vehicle-injected KO (n=16), and KO injected with AAV9-NG276 at either 1e11 vg (n=20) or 3e11 vg/animal (n=17) in the 3-chamber test (A-F). Sociability index (A-C) was evaluated by measuring the time spent with Stranger 1 mouse compared to the empty cage during session 1. Social novelty index (D-F) was evaluated by assessing the time spent with Stranger 2 mouse compared to the now familiar S1 mouse during session 2. For the sociability index, > 0.5 indicates more time spent in the chamber containing Stranger 1. For the social novelty preference index, > 0.5 indicates more time spent in the chamber containing Stranger 2 or a new stranger mouse. Analysis of first 5 min (A, D), second 5 min (B, E) and overall 10 min (C, F) of testing was done. Statistical analysis was performed with a one-way ANOVA followed by Fisher's LSD post-hoc test. All graphs represent average values and the error bars indicate SD. p values reported in the figure represent ANOVA main effects. No difference in sociability index of social preference index of P28-P30 mice was detected in the 3-chamber test.

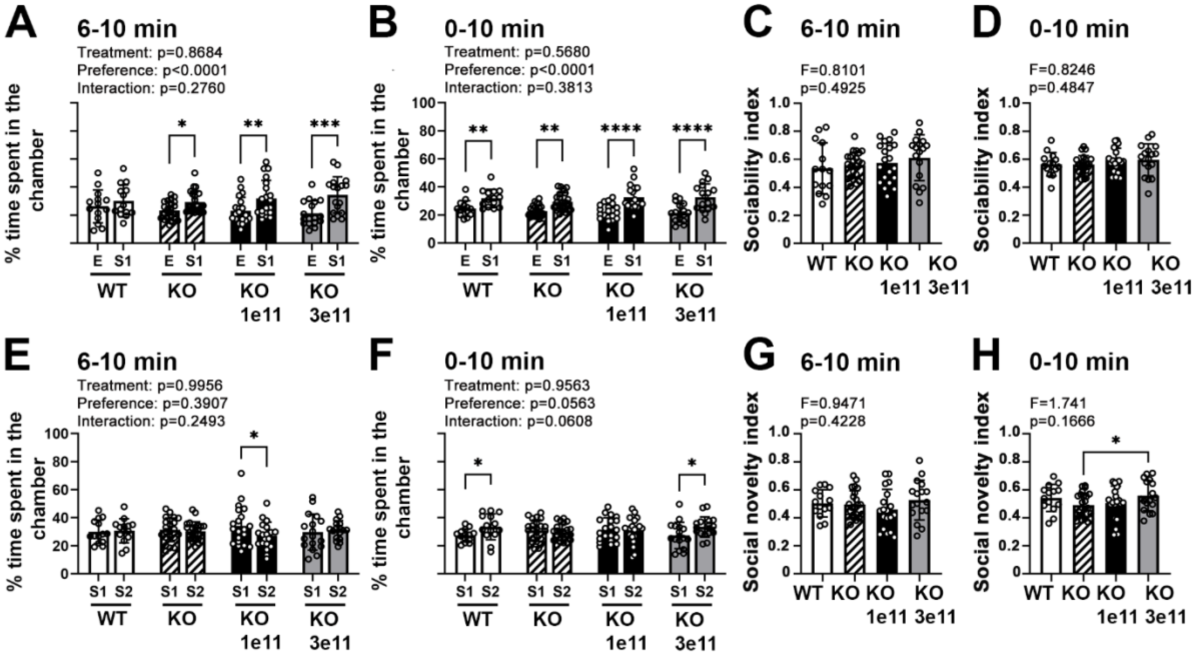

Fig. S15. No difference in sociability and social preference was detected in adult mice during second 5 min of testing.

Graphs show sociability (A, B), sociability index (C, D; evaluated by measuring the time spent with Stranger 1 mouse (S1) compared to the empty cage (E)), social novelty preference (E, F) and social novelty index (G, H; evaluated by assessing the time spent with Stranger 2 mouse (S2) compared to the now familiar S1 mouse (S1)) during second 5 min (A, C, E, G) and overall 10 min (B, D, F, H) in vehicle-injected WT (n=16), vehicle-injected KO (n=16), and KO injected with AAV9-NG276 at either 1e11 vg (n=20) or 3e11 vg/animal (n=17). For the sociability index, > 0.5 indicates more time spent in the chamber containing Stranger 1. For the social novelty preference index, > 0.5 indicates more time spent in the chamber containing Stranger 2 or a new stranger mouse. Statistical analysis was performed with a one-way (C, D, G, H) or two-way (A, B, E, F) ANOVA followed by Fisher's LSD post-hoc test. *p < 0.05, **p < 0.01, ***p < 0.001, ****p < 0.0001. All graphs represent average values and the error bars indicate SD. p values reported in the figure represent ANOVA main effects. No difference in sociability and social preference was detected in adult mice during second 5 min or overall 10 min of testing.

Supplemental tables

Table S1_Statistics for Figure 1

Table S2_Statistics for Figure S2

Table S3_Statistics for Figure 2

Table S4_Statistics for Figure S3

Table S5_Statistics for Figure S4

Table S6_Statistics for Figure 5

Table S7_Statistics for Figure S6

Table S8_Statistics for Figure S10

Table S9_Statistics for Figure S11

Table S10_Statistics for Figure 6

Table S11_Statistics for Figure S12

Table S12_Statistics for Figure S13

Table S13_Statistics for Figure S14

Table S14_Statistics for Figure 7

Table S15_Statistics for Figure S15
