# Supplementary material for: Neonatal expression of human FMRP isoform corrects cortical deficits and improves behavior in a mouse model of fragile X syndrome
Source: Mol Ther Nucleic Acids. 2026 Jun 12;37(3):102981. doi: 10.1016/j.omtn.2026.102981 (PMC13320503; doi:10.1016/j.omtn.2026.102981)
Supplement: Document S2. Article plus supplemental information [file mmc17.pdf]

Molecular Therapy
Nucleic Acids
Original Article

Neonatal expression of human FMRP isoform corrects cortical deficits and improves behavior in a mouse model of fragile X syndrome

Anna O. Norman,[1,6] Courtney Scaramella,[2,3,6] Dominik Biezonski,[4] Ralph D. Hector,[5] Alexandra Varallo,[1] Aarushi Sahni,[1] Nadia Farooq,[1] Suzanne R. Burstein,[4] Juliana Benito,[4] Khaleel A. Razak,[2,3] Jim Selfridge,[5] Stuart Cobb,[4,5] and Iryna M. Ethell[1,2]

[1]Division of Biomedical Sciences, University of California School of Medicine, Riverside, CA 92521, USA; [2]Graduate Neuroscience Program, University of California Riverside, Riverside, CA 92521, USA; [3]Department of Psychology, University of California Riverside, Riverside, CA 92521, USA; [4]Neurogene Inc., New York, NY 10011, USA; [5]Simons Initiative for the Developing Brain, Institute for Neuroscience and Cardiovascular Research, University of Edinburgh, Edinburgh EH8 9XD, UK

Fragile X syndrome (FXS) is a neurodevelopmental disorder caused by CGG trinucleotide repeat expansion in the *fragile X messenger ribonucleoprotein 1* (*FMR1*) gene and the resulting loss of fragile X messenger ribonucleoprotein (FMRP). Gene therapy using recombinant adeno-associated virus (AAV) to restore FMRP expression, particularly in the brain, is a promising therapeutic strategy targeting the underlying cause of FXS. We examined the impact of AAV serotype 9 (AAV9)-mediated expression of a brain-abundant human FMRP isoform (isoform 7) driven by a fragment of the human FMR1 promoter on circuit and behavioral dysfunctions in the male *Fmr1* knockout (KO) mouse, FXS model. Following intracerebroventricular (i.c.v.) injection of AAV9-NG276 into neonatal KO mice at a low (1e11 vg/animal) or high (3e11 vg/animal) dose, we assessed cortical phenotypes using electroencephalography (EEG) recordings and behavioral testing. High-dose AAV9-NG276 normalized baseline gamma power, improved sound-evoked responses, and reduced background neural activity. Analysis of behavioral deficits in adult KO mice showed that high-dose neonatal AAV9-NG276 delivery normalized exploratory behaviors, social preference, and probabilistic reversal learning. Thus, early AAV-mediated delivery of human *FMR1* isoform 7 ameliorates cortical dysfunction and behavioral deficits in a murine FXS model and suggests that widespread cortical biodistribution is required for therapeutic benefit.

INTRODUCTION

Fragile X syndrome (FXS) is the most common single-gene cause of autism spectrum disorder (ASD).[1–3] FXS mostly occurs due to CGG repeat expansion in the 5′ prime untranslated region (5′ UTR) of *fragile X messenger ribonucleoprotein 1* (*FMR1*), resulting in methylation of the promoter region, transcriptional silencing, and a significant reduction or loss of fragile X messenger ribonucleoprotein (FMRP).[4,5] FMRP is an RNA-binding protein that regulates numerous mRNAs critical for synapse formation, maturation, and function.[6–9] Loss of FMRP results in abnormal synaptic function and increased cortical hyperexcitability, which may underlie sensory hypersensitivity.[10] Indeed, hypersensitivity to sensory stimuli is a prominent feature in FXS[11] and has been demonstrated in different sensory domains, including auditory, visual, and somatosensory cortices.[12–17] In addition, many individuals with FXS exhibit increased susceptibility to seizures, anxiety, intellectual disability, developmental delays, repetitive behaviors, and social communication deficits.[18–20]

Current treatments for FXS are limited to pharmacotherapeutics that suppress symptoms, such as anxiety, hyperactivity, irritability, aggression, depression, and seizures.[21] Existing treatments have limited efficacy, induce deleterious side effects, and do not address the root cause of the disorder.[22,23] Viral vector-mediated gene therapy using recombinant adeno-associated virus (rAAV) represents another therapeutic approach and is increasingly considered to be a promising long-term, comprehensive treatment.[24,25] Several studies have explored AAV-mediated expression of FMRP in rodent models via neonatal intracerebroventricular (i.c.v.) or intrathecal (IT) and adult intravenous (i.v.) injections of an AAV vector encoding mouse and rat FMRP homologs. These studies showed partial or full correction of specific biochemical and physiological phenotypes as well as abnormal behaviors.[21,26–31]

In this study, we tested for the first time a novel gene therapy expressing a human *FMR1* isoform 7 under control of a critical fragment of the human *FMR1* promoter (AAV9-NG276) in an *Fmr1* knockout (KO) mouse model. Among over 20 transcript isoforms of human *FMR1* detected *in vivo*,[32] we selected human *FMR1* isoform 7 as our transgene sequence for two reasons: (1) isoform 7 is thought to be one of two most abundant isoforms in adult brain tissue, and

Received 1 April 2026; accepted 9 June 2026;
https://doi.org/10.1016/j.omtn.2026.102981.

[6]These authors contributed equally

Correspondence: Iryna M. Ethell, Division of Biomedical Sciences, University of California Riverside, School of Medicine, Riverside, CA, USA.
E-mail: iryna.ethell@medsch.ucr.edu

© 2026 The Author(s). Published by Elsevier Inc. on behalf of The American Society of Gene and Cell Therapy. This is an open access article under the CC BY license (http://creativecommons.org/licenses/by/4.0/).

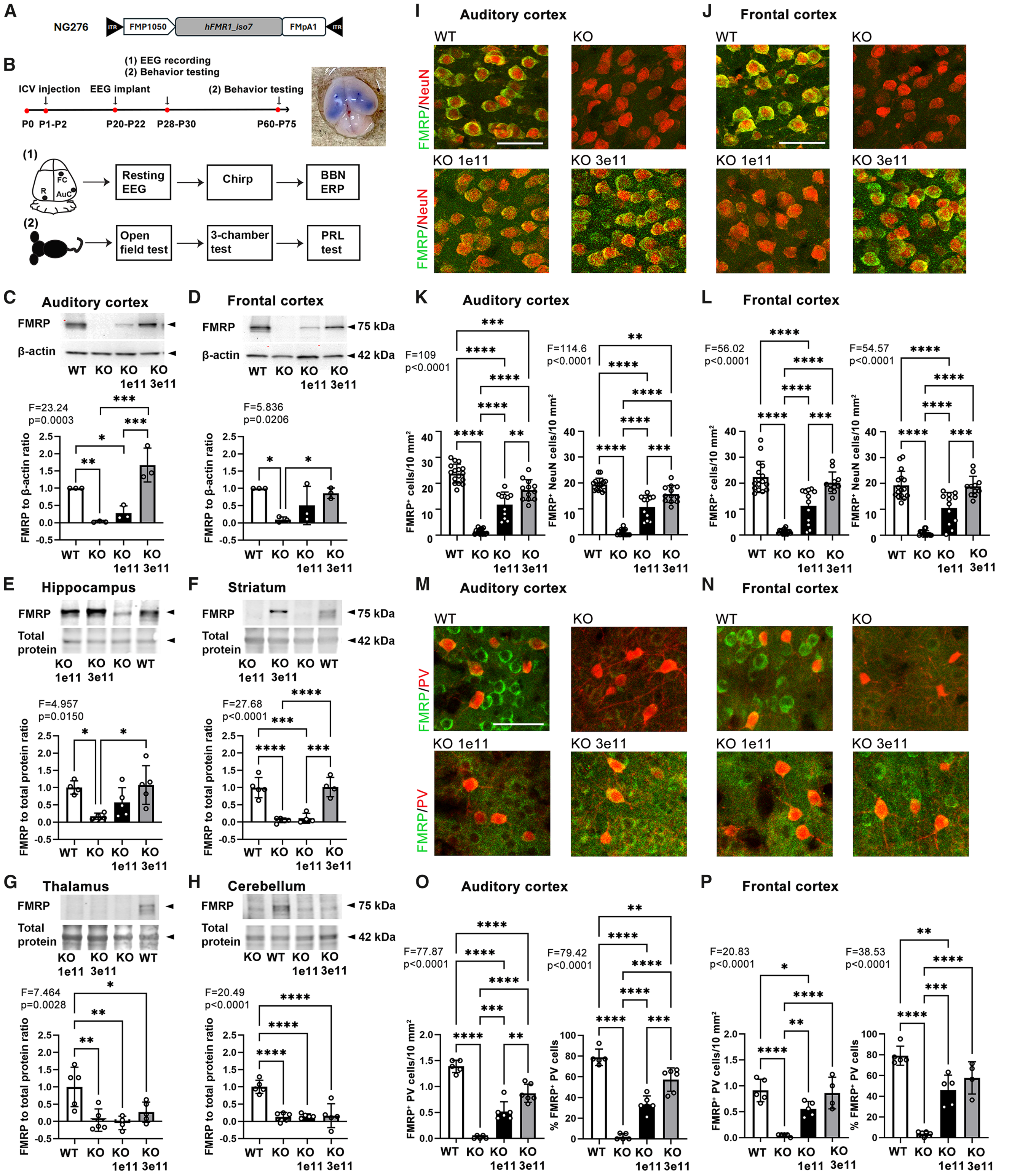

(legend on next page)

(2) isoform 7 lacks exon 12 (due to alternative splicing of human *FMR1*).[32,33] Isoforms lacking exon 12 show an increased affinity for kissing complex RNA.[32,34,35] AAV9-NG276 was delivered neonatally via i.c.v. injection to target transduction of the brain. We found that neonatal delivery of AAV9-NG276 at a high dose of 3e11 vg/mouse normalized baseline gamma power, improved consistency of timing of evoked responses to spectrotemporally modulated sounds in gamma frequencies, reduced background neural activity, and improved habituation to repeated stimuli showing a dose-dependent reduction in ongoing cortical responses in adolescent KO mice. Moreover, we showed that the treatment at a high dose of 3e11 vg/mouse normalized both innate and learned behaviors, including exploratory behaviors, social preference, and reversal learning in adult KO mice. These findings highlight the potential for AAV9-NG276 to ameliorate neurocircuit deficits that are likely critical in driving FXS symptoms in humans.

RESULTS

Neonatal i.c.v. delivery of AAV9-NG276 is sufficient to express FMRP in the auditory and frontal cortex of KO mice to WT levels

We first assessed the efficacy of an AAV-based approach to deliver human FMRP in the KO mouse model. AAV9-NG276, an AAV9 vector that expresses human *FMR1* isoform 7 driven by a fragment of the human *FMR1* promoter (Figure 1A; Figures S1A and S1B), was delivered to neonatal KO mice by i.c.v. injections at two doses: 1e11 or 3e11 vg/mouse. Wild-type (WT) and KO control groups received vehicle. The study plan is described in Figure 1B. Electrophysiology recordings were performed at postnatal day (P)28–P30, and behavioral testing was performed in adolescent mice at P28–P30 and adult mice at P60–P75 (Figure 1B). Vector genome biodistribution was evaluated by qPCR following electroencephalography (EEG) recording (Figure S1C) and after behavioral testing (Figure S1D). Dose-dependent vector genome biodistribution was observed throughout the brain across key CNS regions implicated in FXS pathogenesis (Figures S1C and S1D).

FMRP expression was also evaluated by western blot in 3–5 animals per group at P28–P30 (after EEG) and P70–P75 (after behavioral testing). In the auditory cortex (AuC) and frontal cortex (FC), FMRP protein was expressed in a dose-dependent manner (Figures 1C and 1D; Table S1). At the high dose, FMRP expression was detected at near-WT levels in both the AuC and FC (Figures 1C and 1D; Table S1). As expected, FMRP was not detected in vehicle-treated KO animals. At the high dose, FMRP expression was also detected in the hippocampus and striatum (Figures 1E and 1F; Table S1) but not thalamus and cerebellum of P28–P30 KO mice (Figures 1G and 1H; Table S1). Interestingly, a low-dose treatment was sufficient to achieve 50% of WT FMRP levels in the hippocampus (Figure 1E; Table S1). In addition, we assessed vector-derived FMRP expression in the AuC and FC of adolescent (P28–P30) WT and KO mice by immunohistochemistry (IHC). Treatment of KO mice with AAV9-NG276 (high and low dose) resulted in a significant increase in the density of $FMRP^+$ cells and $FMRP^+$ $NeuN^+$ neurons compared to vehicle-injected KO. However, $FMRP^+$ cell density in both the AuC and FC was lower in KO animals administered the low dose compared to those receiving the high dose (Figures 1I–1L; Table S1). Interestingly, the effects of the treatment were greater in the FC where the density of $FMRP^+$ cells reached WT levels (Figures 1I–1L; Table S1). While the majority of $FMRP^+$ cells were NeuN positive in the WT group (82.86% ± 10.55% AuC, 86.12% ± 7.56% FC) and KO group treated with the high dose (90.81% ± 5.18% AuC, 93.18% ± 2.49% FC, Figures S2A and S2C; Table S2), the remaining $FMRP^+$ cells most likely represent glial cells, indicating broader expression of FMRP in different cell types. Analysis of neuron-specific FMRP expression showed a significantly higher percentage of NeuN-positive cells expressing the transgene in the high-dose KO (69.87% ± 8.09% FC; 64.27% ± 6.24% AuC) compared to the low-dose KO group (45.94% ± 24.16% FC; 54.30% ± 23.96% AuC), although it remained lower than in the WT group (91.99% ± 5.59% FC; 91.06% ± 6.16% AuC, Figures S2B and S2D; Table S2). As the development of parvalbumin (PV)-expressing cells is affected in the AuC of KO mice,[36,37] we also assessed FMRP levels in PV cells

Figure 1. FMRP levels in the AuC and FC of P28–P30 WT and KO mice intracerebroventrically injected with vehicle (control) or AAV9-NG276 at low and high dose

(A) AAV9-NG276 construct design incorporating the endogenous human *FMR1* promoter (FMP1050) and 3′ UTR (*FMpA1*) fragments. These regulatory elements drive and regulate expression of a human *FMR1* isoform 7 transgene (*hMFR1_iso7*). The construct is flanked by AAV2 inverted terminal repeat sequences (ITRs). (B) Schematic shows the experimental timeline of bilateral i.c.v. injections, EEG recordings, and behavioral testing. Note that different groups of mice were used for EEG recordings (1) and behavior tests (2). (1) EEG electrodes were implanted in the auditory cortex (AuC), frontal cortex (FC), and occipital lobe (reference electrode, R) at P20–P22, and EEG recordings were performed at P28–P30, in the following order: resting EEG (no sound), chirp, and broadband noise (BBN). (2) Mouse behavior was assessed at P60–P75 in the following order: open field test, 3-chamber test, and probabilistic reversal learning (PRL) test. Image shows the injected sites and the distribution of blue dye within the ventricles following i.c.v. injection of P1–P2 pups with the buffer containing trypan blue. (C and D) Western blot images of FMRP and β-actin, and quantitative analysis of FMRP levels in P70–P75 AuC (C) and FC (D) of vehicle-injected WT, vehicle-injected KO, and KO mice injected with AAV9-NG276 at either 1e11 (low dose) or 3e11 (high dose) vg/animal (n = 3 mice per group). (E–H) Western blot images of FMRP and total protein (β-actin band), and quantitative analysis of FMRP levels in P28–P30 hippocampus (E), striatum (F), thalamus (G), and cerebellum (H) of vehicle-injected WT, vehicle-injected KO, and KO mice injected with AAV9-NG276 at either 1e11 (low dose) or 3e11 (high dose) vg/animal (n = 4–5 mice per group). (I and J) Confocal images showing FMRP (green) and NeuN (red) immunoreactivity in P28–P30 AuC (I) and FC (J) of vehicle-injected WT, vehicle-injected KO, and KO mice injected with AAV9-NG276 at low and high doses. Scale bars, 50 μm. (K and L) Quantitative analysis of density of $FMRP^+$ cells and $FMRP^+$ NeuN cells in AuC (G) and FC (H, n = 10–15 images from 3 to 5 mice per group). (M and N) Confocal images showing FMRP (green) and PV (red) immunoreactivity in P28–P30 AuC (M) and FC (N) of vehicle-injected WT, vehicle-injected KO, and KO mice injected with AAV9-NG276 at low and high doses. Scale bars, 50 μm. (O and P) Quantitative analysis of density of $FMRP^+$ PV cells and percentage of $FMRP^+$ PV cells in the AuC (O) and FC (P, n = 4–6 images from 3 mice per group). Statistical analysis was performed using one-way ANOVA with a Tukey's post hoc test: *$p < 0.05$, **$p < 0.01$, ***$p < 0.001$, ****$p < 0.0001$. Data represent mean ± standard deviation (SD). p values reported in the figure represent ANOVA main effects. The FMRP levels and the density of FMRP-positive neurons were significantly increased in the AuC and FC of high-dose KO group. FMRP immunoreactivity was also significantly increased in the low-dose KO group but did not reach WT levels.

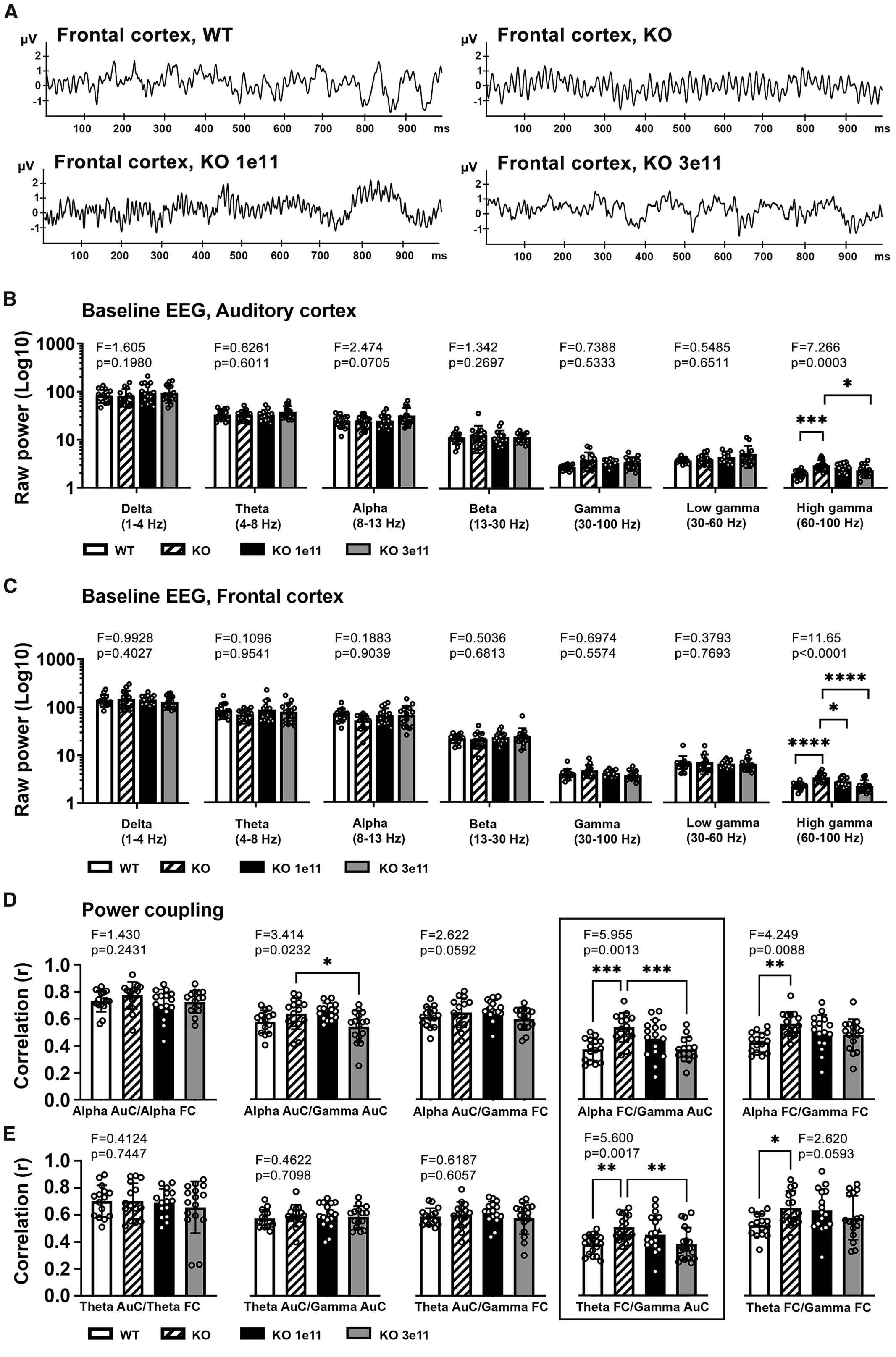

(legend on next page)

in the AuC and FC of P28–P30 mice. We detected FMRP expression in PV cells in both low- and high-dose KO groups. There were 30%–40% PV cells expressing FMRP in a low-dose group and around 60% FMRP$^+$ PV cells in a high-dose KO group compared to 80% FMRP$^+$ PV cells in the WT group in both the AuC and FC (Figures 1M–1P; Table S1). We also detected FMRP immunoreactivity in the cortex, hippocampus, and striatum, but not thalamus, of adult P70–P75 mice after behavioral testing (Figure S2E).

Together, FMRP expression was increased to near-WT levels in the cortex, hippocampus, and striatum in the high-dose group and to intermediate levels in the low-dose group. Results were consistent in adolescent (P28–P30) and adult (P70–P75) mice with both western blot and IHC.

FMRP expression restored cortical resting-state EEG power in the high-gamma range to WT levels

We have previously shown that both adolescent and adult KO mice[38–40] exhibit increased baseline gamma power, a phenomenon that is also observed in FXS individuals.[41–43] Here, we assessed whether vehicle-injected KO mice also exhibit increased baseline gamma power and determined the effect of AAV9-NG276 treatment on this phenotype. We found increased baseline power of gamma oscillations (60–100 Hz) in both the AuC and FC of KO mice compared to WT mice (Figures 2A–2C; Table S3), which is consistent with previous findings.[38–40] Compared to vehicle-injected KO mice, treatment of KO mice with high-dose AAV9-NG276 resulted in a significant decrease in the power of high-gamma oscillations in the AuC and FC to WT levels, with a greater effect in the FC. We also observed partial effects in the low-dose group, showing a slight but significant decrease in the power of gamma oscillations in the FC. Although we did not see significant differences between KO and low-dose KO groups in the AuC, we found a negative correlation between FMRP protein levels and high gamma power (Figures S3A and S3B; Table S4) with mice showing higher FMRP levels having lower high gamma power. No changes in other frequencies were observed (Figures 2B and 2C; Table S3). Since elevated resting gamma power relates to background "noisy" cortical activity,[44] these results suggest an amelioration of this FXS-related phenotype in adolescent KO mice injected with high-dose AAV9-NG276.

Human EEG studies demonstrated impaired power coupling between low- and high-frequency oscillations in individuals with FXS and ASD.[11,43] Here, we analyzed power coupling between low (theta, or alpha) and high (gamma) frequency bands in the AuC and FC as well as between these two cortical areas (Figures 2D and 2E; Figure S4; Tables S3 and S5). These measures indicate how the activity of neuronal populations within and across cortical regions is coordinated through oscillatory rhythms. Consistent with prior reports in KO mice and humans with FXS,[39,45] we found elevated alpha/gamma and theta/gamma coupling at rest compared to controls (Figures 2D and 2E; Table S3), which was significantly improved in the high-dose group to WT levels. This rescue was not observed in the low-dose group. Regional and frequency-specific low-/high-frequency power coupling is important in coordinating activity within and across active neural populations and is critical for specific sensory and cognitive tasks.[46,47] Our data suggest a normalization of such coupling in the high-dose group.

Phase locking to the chirp was significantly improved in the AuC and FC of adolescent KO mice following human FMRP expression

We next assessed the effect of AAV9-NG276 neonatal FMRP expression on the fidelity of temporal processing to time-varying stimuli, which was previously shown to be impaired in KO mice.[37,39,48] This was accomplished by testing responses to "up chirps" in the cortex of adolescent mice via EEG recordings.

In the AuC, we observed a significant decrease in phase locking to the chirp in KO mice compared to WT mice in two main clusters that included the low-gamma band (30–55 Hz) and high-gamma band (60–80 Hz, Figures 3A and 3B). The low-dose group also showed a similar decrease compared to WT and was not different from the KO buffer group (Figures 3A–3C). However, there was a significant improvement in the high-dose group compared to vehicle-injected KO or low-dose groups in the 60–80 Hz high-gamma band (Figures 3A and 3C; Figure S5A), with the deficit still observed in the 30–55 Hz low-gamma band compared to the vehicle-injected WT group (Figures 3A and 3B).

In the FC, there was also a significant decrease in phase locking to the chirp in KO mice compared to WT mice in the gamma band (40–90 Hz; Figures 3D and 3E). The low-dose group also showed a significant decrease compared to WT and was not different from the KO buffer group (Figures 3D–3F). There was a significant

Figure 2. Resting-state high gamma power was enhanced in the P28–P30 AuC and FC of KO mice but was normalized to WT levels in the high-dose group
(A) Graphs show representative EEG traces from the FC of vehicle-injected WT (left) and vehicle-injected KO (right) mice. The resting-state frequency power of EEG traces was enhanced in the FC of vehicle-injected KO compared to WT mice. (B and C) Graphs show average frequency power in the (B) AuC and (C) FC of vehicle-injected WT (n = 15), vehicle-injected KO (n = 15), KO mice injected with AAV9-NG276 at either 1e11 vg (n = 16; low dose) or 3e11 vg/animal (n = 16; high dose). (D and E) Graphs show Pearson's correlation (r) for low-/high-frequency power coupling: alpha/alpha, and alpha/high gamma (D), or theta/theta and theta/high gamma (E) in the AuC or FC as well as between these two cortical areas. Values were grouped by frequency bands and analyzed with one-way ANOVA with Bonferroni's post hoc test. *$p < 0.05$, **$p < 0.01$, ***$p < 0.001$, ****$p < 0.0001$. Data represent mean ± standard deviation (SD). p values reported in the figure represent ANOVA main effects. Resting-state high gamma power (60–100 Hz) was enhanced in the AuC and FC of KO compared to WT mice, and it was normalized in the AuC and FC of KO 3e11 and FC of KO 1e11. No differences were detected in other frequencies. Alpha/gamma and theta/gamma frequency power coupling across the regions was impaired in KO mice and normalized to WT levels in KO 3e11 mice.

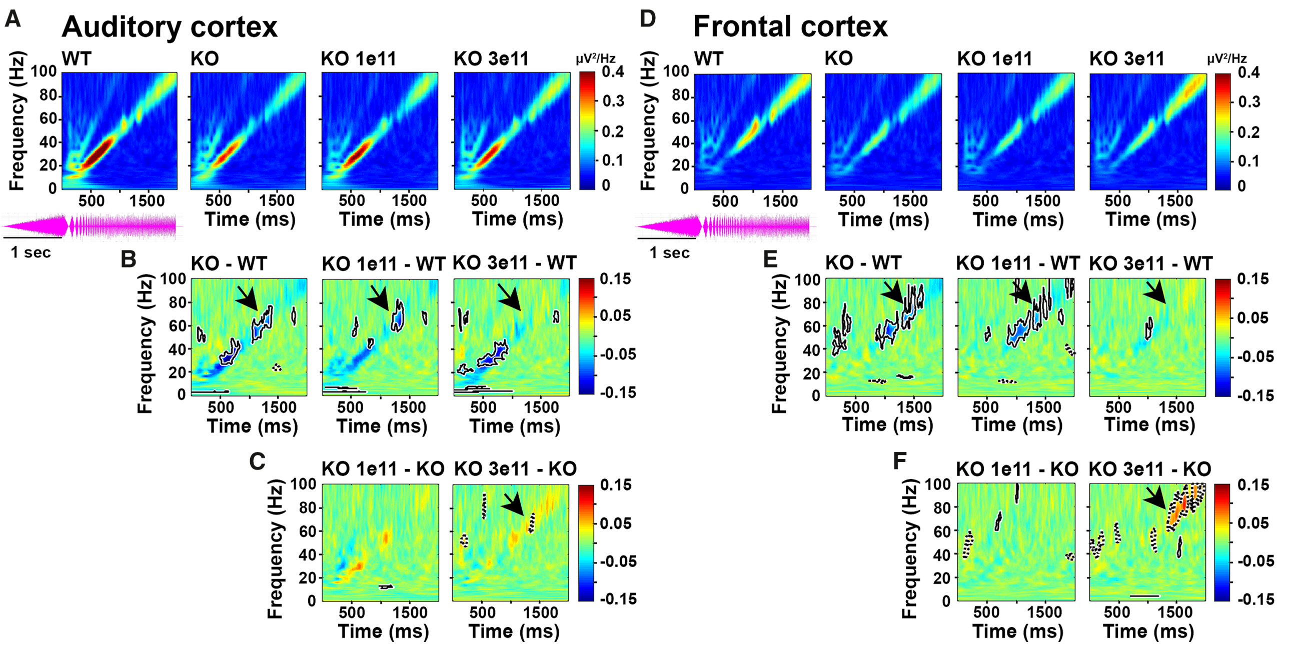

Figure 3. Impaired phase locking to auditory "up" chirp stimuli was significantly improved in high-gamma 60–100 Hz range in the P28–P30 AuC and FC of the high-dose group

(A and D) Inter-trial phase coherence (ITPC) grand average for vehicle-injected WT (n = 15), vehicle-injected KO (n = 15), and KO mice injected with AAV9-NG276 at either 1e11 vg (n = 16; low dose) or 3e11 vg/animal (n = 16; high dose) in the AuC (A–C) and FC (D–F). (B, C, E, and F) Graphs show comparisons to vehicle-injected WT mice (B and E): KO/WT, low dose/WT, high dose/WT; and to vehicle-injected KO mice (C and F): low dose/KO and high dose/KO. Blue areas indicate a decrease, green indicates no difference, and orange/red indicates an increase. Significant clusters ($p < 0.025$) are highlighted by bold-lined contours (the solid line depicts a significant decrease, and the dotted line depicts a significant increase). Phase locking to up-chirp stimuli was decreased in the AuC and FC of KO compared to WT mice, and it was significantly improved to WT levels in high-gamma range in the AuC and FC of KO 3e11 but not KO 1e11 group (depicted by arrows).

improvement in the high-dose group compared to the vehicle-injected KO or low-dose groups in the 60–100 Hz high-gamma range (Figures 3D–3F; Figure S5C). While the response was also improved in the 40–60 Hz gamma range, it was still reduced compared to the WT group (Figures 3D–3F). Together, these data indicate improved phase locking to spectrotemporally dynamic sounds in both the AuC and FC of the high-dose group.

To examine whether the juvenile KO mice also display increased background power during the chirp presentation similar to adult KO mice[38] and the impact of AAV9-NG276, we assessed non-phase-locked single-trial power (STP) during chirp stimulation in the AuC and FC. We found that P28–P30 KO mice exhibit a significant increase in STP in the AuC and FC in the beta to gamma range (20–100 Hz) compared to WT control mice (Figures 4A–4F). Treatment of KO mice with high-dose AAV9-NG276 did not alter background power in the AuC, which was still similar to that of KO and low-dose KO mice (Figures 4A and 4B; Figure S5B). However, FC showed a significant improvement in the high-dose group compared to either the KO buffer or low-dose groups in the entire 20–100 Hz range, with a similar trend observed in the AuC (Figures 4D and 4F; Figure S5D). In both the AuC and FC, the background power in the high-dose group was not significantly different from that of the WT group (Figures 4A, 4B, 4D, and 4E).

Together, our data show that P28–P30 KO mice exhibit impaired consistency of timing in responses to spectrotemporally modulated sounds, particularly for low- and high-gamma oscillations, and increased background gamma power in both the AuC and FC. This phenomenon is also observed in adult KO mice as well as humans with FXS.[38,41,42,48] High-dose AAV9-NG276 ameliorated the deficits in timing of the responses to chirp in the high-gamma range and reduced background gamma power in the AuC and FC of KO mice.

Responses to broadband noise were normalized in the P28–P30 FC of the high-dose group

The sound-evoked responses during repeated auditory stimulus presentation were reported to be higher in humans with FXS than in healthy controls as well as in mouse models of FXS.[36,38,49,50] Here, we assessed whether a similar phenotype occurs in P28–P30 vehicle-injected WT and KO mice by analyzing responses to broadband noise. To determine if there was an effect of AAV9-NG276 on the amplitudes and latencies of the P1, N1, and P2 components of the event-related potential (ERP), we first analyzed responses to 0.25 and 4 Hz sound trains (Figures 5A–5L; Figures S6A–S6L; Tables S6 and S7). We found that P28–P30 KO mice exhibit a significant increase in N1 amplitude in response to sound with 0.25 and 4 Hz repetition rates in

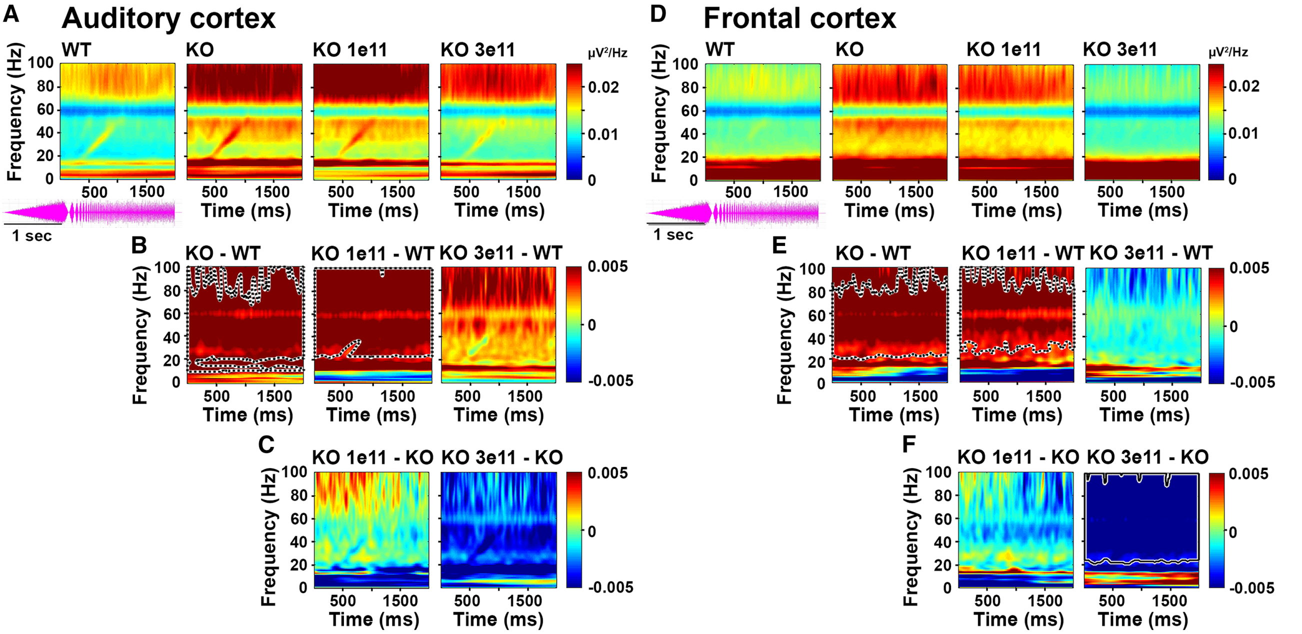

Figure 4. Non-phase-locked (background) power during auditory up chirp stimuli was significantly enhanced in the AuC and FC of KO mice and was normalized to WT levels in the high-dose group

(A and D) Single-trial power (STP) grand average for vehicle-injected WT (n = 15), vehicle-injected KO (n = 15), and KO mice injected with AAV9-NG276 at either 1e11 vg (n = 16; low dose) or 3e11 vg/animal (n = 16; high dose) in the AuC (A–C) and FC (D–F). (B, C, E, and F) Graphs show comparisons to vehicle-injected WT mice (B and E): KO/WT, low dose/WT, high dose/WT; and to vehicle-injected KO mice (C and F): low dose/KO and high dose/KO. Blue areas indicate a decrease, green indicates no difference, and orange/red indicates an increase. Significant clusters (p < 0.025) are highlighted by bold-lined contours (the solid line depicts a significant decrease, and the dotted line depicts a significant increase). Background power during up-chirp stimuli was significantly increased in the AuC and FC of KO compared to WT mice, and it was normalized to WT levels in the AuC and FC of KO 3e11 but not KO 1e11 group.

the FC compared to WT control mice (Figures 5E and S6E; Tables S6 and S7), but we observed no difference in the latency between the WT and KO groups (Figures 5G–5L; Figures S6G–S6L; Tables S6 and S7). Treatment of KO mice with low-dose AAV9-NG276 did not correct the deficits in N1 amplitude (Figures 5E and S6E; Tables S6 and S7). However, there was a significant decrease in N1 amplitude in the high-dose group compared to the KO buffer group in the FC, which was not different from the WT group (Figures 5E and S6E; Tables S6 and S7). This indicates a reduction in hyperresponsiveness to sounds with the treatment.

In addition, we found that, compared to WT mice, adolescent KO mice exhibit a significant increase in the power of onset (0–50 ms) and ongoing (50–500 ms) responses to 0.25 Hz sounds in the 30–100 Hz gamma range in the AuC and FC (Figures S7A, S7B, S7D, and S7E). Treatment of KO mice with low-dose AAV9-NG276 did not correct the power, which was still significantly higher compared to WT mice and similar to KO mice (Figures S7A–S7F). However, there was a significant improvement in the high-dose group compared to the low-dose group in the AuC and FC for both onset and ongoing responses that were not significantly different from the WT group (Figures S7B, S7E, S9A, and S9C).

We also found that, when compared to WT control mice, P28–P30 KO mice exhibit a significant increase in the power of onset and ongoing responses to sounds with a 4 Hz repetition rate in the 30–100 Hz gamma range in the AuC and FC (Figures S8A, S8B, S8D, and S8E). Treatment of KO mice with low-dose AAV9-NG276 did not correct the power in the AuC or FC, which was still significantly higher than in WT mice (Figures S8A, S8B, S8D, and S8E). There was a significant improvement in the FC of the high-dose group compared to both the KO buffer and low-dose groups that was not significantly different from the WT group (Figures S8A–S8F, S9B, and S8D).

In summary, adolescent KO mice exhibit a significant increase in N1 amplitude and overall power in the 30–100 Hz gamma range in response to sound trains with 0.25 and 4 Hz repetition rates in the AuC and FC compared to WT mice. Treatment with high-dose AAV9-NG276 ameliorated these deficits in the FC, with a similar trend in the AuC. In the FC, there was a significant improvement in the overall power of the responses in the high-dose group compared to both the KO buffer and low-dose groups. In addition, N1 amplitude was also significantly decreased in the FC of the high-dose group, compared to the vehicle-injected KO group, and was restored to the normal WT level.

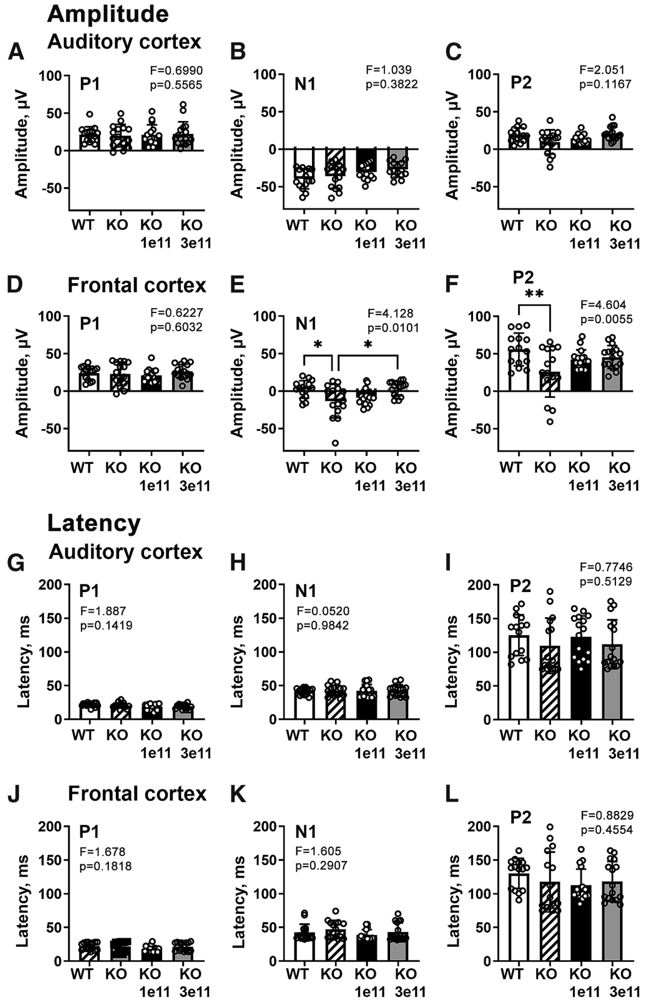

Figure 5. N1 amplitude of auditory ERP was enhanced in the FC of P28–30 KO mice and was significantly reduced to WT levels in the high-dose group

(A–L) Auditory ERP amplitudes (A–F) and latencies (G–L) in the AuC (A–C and G–I) and FC (D–F and J–L) of vehicle-injected WT (n = 15), vehicle-injected KO (n = 15), and KO mice injected with AAV9-NG276 at either 1e11 vg (n = 16; low dose) or 3e11 vg/animal (n = 16; high dose). Grand average ERPs obtained from P28–P30 mice in response to 100 ms broadband noise presented at a 0.25 Hz repetition rate. P1, N1, and P2 were defined as maximum or minimum voltage deflections within 0–30, 30–80, or 80–150 ms, respectively. Statistical analysis was performed with a one-way ANOVA and Bonferroni post hoc test. *$p < 0.05$, **$p < 0.01$. Data represent mean ± standard deviation (SD). p values reported in the figure represent ANOVA main effects. N1 amplitude was elevated in the FC of KO compared to WT mice, and it was normalized to WT levels in the high-dose group.

Exploratory behaviors were normalized to WT levels in adult KO mice treated with high-dose AAV9-NG276

Humans with FXS as well as KO mice display multiple behavioral impairments including hyperactivity, increased anxiety, and altered social behaviors.[48,51,52] Here, we assessed whether adolescent (P28–P30) and adult (P60–P65) vehicle-injected KO mice also exhibit increased locomotor activity and altered exploratory behaviors compared to their WT littermates and determined the effect of the neonatal injection of AAV9-NG276 on these phenotypes. We used an open field test (Figure 6A) to evaluate exploratory behaviors by analyzing the time spent in the center of the arena (%), the number of crosses through the center of the arena, the time spent in the perimeter of the arena (thigmotaxis), and the time spent in the open field (open field arena without thigmotaxis). Locomotor activity was assessed by analyzing the overall distance traveled and velocity in the arena. Since we did not observe a model effect in P28–P30 mice (Figures S10A–S10I and S11A–S11F; Tables S8 and S9), we further studied the therapeutic impact of neonatal AAV9-NG276 i.c.v. treatment on locomotor activity and exploratory behaviors in P60–P65 mice, or 2 months following the treatment (Figures 6A–6E; Figures S12A–S12J; Tables S10 and S11). We found that vehicle-injected KO mice spent more time in the center of the arena (Figure 6B; Table S10) and made more crosses through the center of the arena (Figure 6C; Table S10), which is consistent with previous reports.[51,53–55] Treatment of KO mice with high-dose AAV9-NG276, but not low-dose AAV9-NG276, showed beneficial effects, decreasing their time spent in the center of the arena and the number of crosses through the center compared to control KO mice (Figures 6B and 6C; Table S10). Since control KO mice spent more time in the center of the open field arena, we further analyzed the percent time spent in thigmotaxis (along the walls) vs. the open field (without thigmotaxis). We found that during the first 5 min of the test, when mice habituated to the arena, control WT, KO, and AAV9-NG276-treated KO mice (at both low and high doses) spent significantly more time in thigmotaxis compared to the open field (Figure 6D; Table S10). However, while WT mice still showed a preference for thigmotaxis during the second 5 min of the test (behaviors considered to be normal as mice usually avoid open spaces), control KO mice showed no such preference and spent less time in thigmotaxis than WT mice (Figure 6E; Table S10). Treatment with high-dose AAV9-NG276, but not low-dose AAV9-NG276, significantly increased the percent time spent in thigmotaxis compared to control KO mice, showing a preference toward thigmotaxis that was similar to WT mice (Figure 6E; Table S10). We did not observe a difference in locomotor activity between control buffer-injected WT and KO mice (Figures S12E–S12J; Table S11). Although we did not observe behavior improvements in the low-dose KO group, we further performed a correlation analysis between FMRP protein levels and behavior. We found a significant correlation between FMRP levels and the percentage of time spent in thigmotaxis or the open field only (Figures S13A and S13B; Table S12). Mice with high FMRP levels (>0.25) showed a preference for thigmotaxis similarly to WT, while mice with low FMRP levels showed no location preference similarly to KO mice (Figure S13C; Table S12).

In summary, although we did not observe the genotype difference in P28–P30 mice, we found that exploratory behaviors were altered in KO mice at P60–P65 and were normalized to WT levels in the high-dose group. In addition, mice with high cortical FMRP expression in the low-dose group also displayed WT-like exploratory behavior.

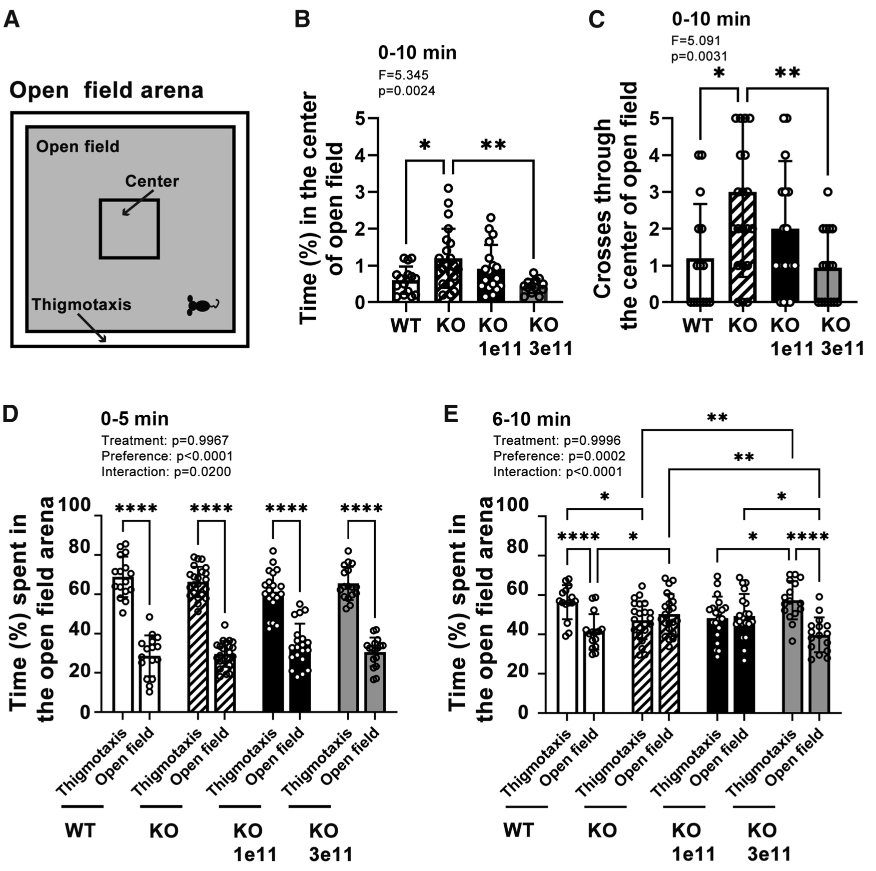

Figure 6. Exploratory behavior in the open field test was impaired in adult KO mice and was normalized to WT levels in the high-dose group

(A) Graphics showing the open field arena, including thigmotaxis, the center of the arena, and open field (an arena without thigmotaxis). (B–E) Graphs demonstrate the performance of vehicle-injected WT (n = 16), vehicle-injected KO (n = 16), and KO mice injected with AAV9-NG276 at either 1e11 vg (n = 20; low dose) or 3e11 vg/animal (n = 17; high dose) in the open field test at P60–P65. Exploratory behaviors were evaluated by analyzing the percentage of time spent in the center of the open field (B), crosses through the center of open field (C), and percentage of time spent in thigmotaxis and open field (D and E) during 10 min of testing (B and C), first 5 min (D) and second 5 min (E). Statistical analysis was performed with a one-way (A–C) or two-way (D and E) ANOVA followed by Tukey's post hoc test. $^{*}p < 0.05$, $^{**}p < 0.01$, $^{****}p < 0.0001$. Data represent mean ± standard deviation (SD). p values reported in the figure represent ANOVA main effects. The vehicle-injected KO mice showed altered exploratory behaviors compared to WT mice (spent more time in the center of the arena, made more crosses through the center of the arena, and spent more time in the open field). Abnormal exploratory behaviors were normalized to WT levels in the high-dose group.

Social novelty preference and probabilistic reversal learning were impaired in adult KO mice and were normalized to WT levels in the high-dose group

Impaired social interactions are common behavioral observations in FXS, especially during development. KO mice were reported to display a reduced preference for novelty in social settings.[48,56] In this study, we evaluated whether P28–P30 and P60–P65 control KO mice had altered sociability and social novelty preference and determined the effect of AAV9-NG276 on these phenotypes in treated animals. We used a social novelty test (3-chamber test) to assess sociability and social novelty preference in mice. At P28–P30, we did not find any significant differences between control WT and KO mice in the sociability index (Figures S14A–S14C; Table S13) or the social novelty preference index (Figures S14D–S14F; Table S13). Since there was no model effect, we were unable to determine if there was an effect of treatment on the social interaction behaviors.

We next studied social behaviors in P60–P65 mice. We did not observe a difference in sociability (by calculating the sociability index) between control WT and KO mice (Figures 7A–7C; Figures S15A–S15D; Tables S14 and S15). Since there was no model effect, we could not determine the effect of treatment with low and high doses of AAV9-NG276. While we also found no difference in the social preference index between control WT and KO during the entire 10 min of testing (Figures S15F and S15H; Table S15), further analysis showed a decreased social novelty index in KO compared to WT mice during the first but not the second 5 min of the testing (Figures 7D–7F; Figures S15E and S15G; Tables S14 and S15). This difference can be explained by the fact that WT mice show a preference for a novel mouse during the first 5 min of testing and lose the preference during the second 5 min, while KO mice show no preference for a novel mouse throughout testing. Analyzing the effect of treatment, we found that KO mice injected with high-dose AAV9-NG276, but not low-dose AAV9-NG276, showed significantly improved performance compared to control vehicle-injected KO mice, with the social novelty index and preference for a novel mouse similar to WT mice (Figures 7D–7F; Table S14). However, further analysis demonstrated that mice with high FMRP levels (>0.25) in a low-dose AAV9-NG276 group spent significantly more time with a novel mouse similarly to WT mice, while mice with low FMRP levels showed no preference (Figure S13D; Table S12).

Learning and cognitive flexibility deficits have also been reported in individuals with FXS as well as KO mice.[57] Probabilistic reversal learning represents one test that could be used as a cross-species paradigm related to FXS to identify altered cognitive processes underlying cognitive inflexibility.[57] The test is used to identify whether a reversal learning deficit results from initial perseveration of the previously learned choice pattern and/or from maintaining the new choice pattern after the initial selection (Figure 7G; Table S14). In

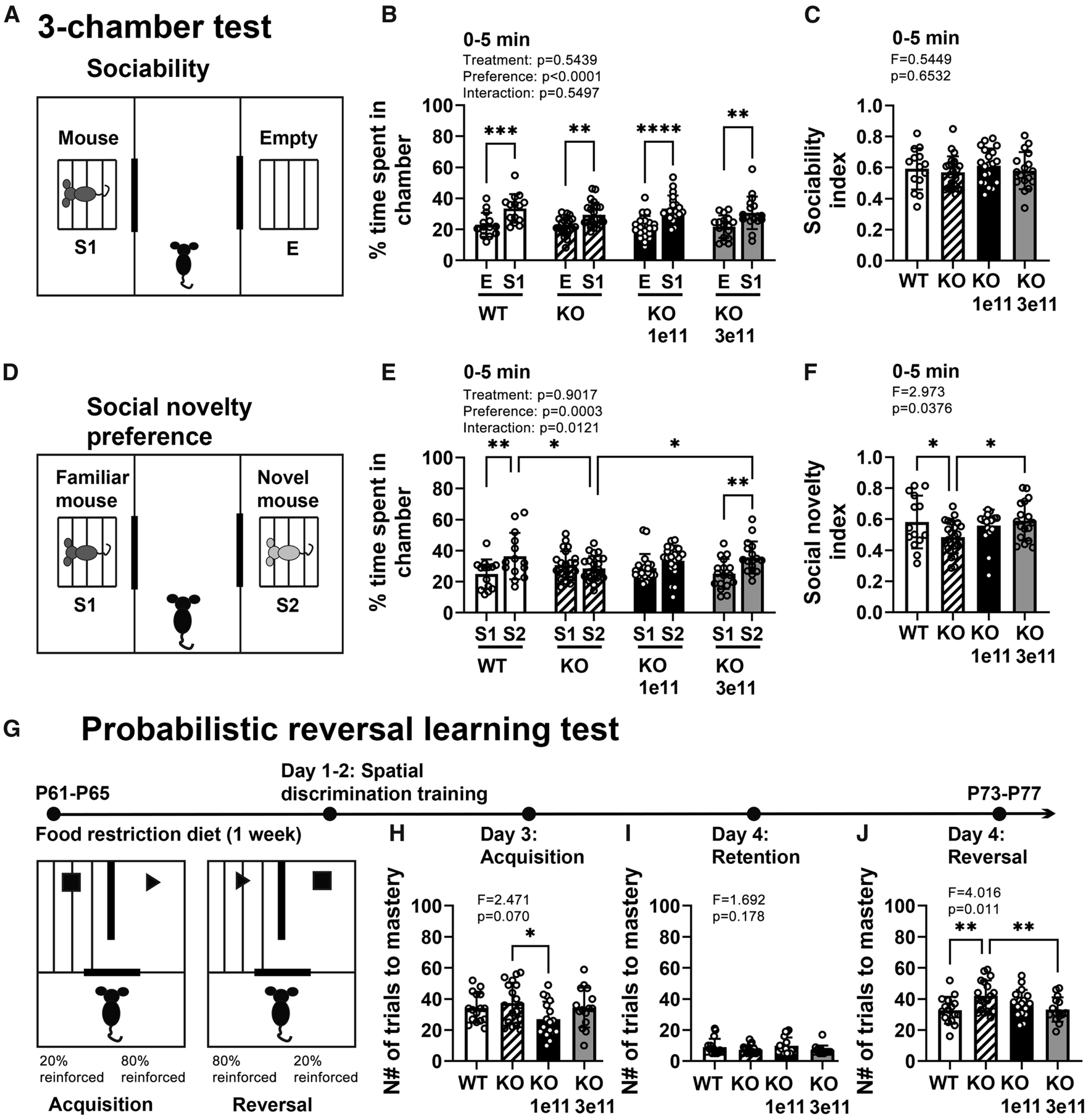

Figure 7. Social novelty preference and probabilistic reversal learning were impaired in adult KO mice and normalized to WT levels in the high-dose group

(A–J) Graphs demonstrate the performance of P60–P65 vehicle-injected WT (*n* = 16), vehicle-injected KO (*n* = 16), and KO injected mice with AAV9-NG276 at either 1e11 vg (*n* = 20; low dose) or 3e11 vg/animal (*n* = 17; high dose) in the 3-chamber (A–F) and probabilistic reversal learning test (G–J). (A and D) Graphics showing the 3-chamber arena for assessing sociability (A) and social novelty preference (D) of mice. Sociability (B) and sociability index (C) were evaluated by measuring the time spent with stranger 1 mouse (S1) compared to the empty cage (E) during session 1 (first 5 min). Social novelty preference (E) and social novelty index (F) were evaluated by assessing the time spent with stranger 2 mouse (S2) compared to the now familiar S1 mouse (S1) during session 2 (first 5 min). For the sociability index, >0.5 indicates more time spent in the chamber containing stranger 1. For the social novelty preference index, >0.5 indicates more time spent in the chamber containing stranger 2 or a new stranger mouse. (G) Graphics showing the timeline of the testing, including food restriction diet for 1 week, spatial discrimination training on days 1–2, acquisition on day 3, and both retention testing and reversal training on day 4. (H–J) Graphs demonstrate assessing acquisition (H), retention (I), and reversal learning (J). Statistical analysis was performed with a one-way (C, F, and H–J) or two-way (B and E) ANOVA followed by Fisher's LSD post hoc test. *$p < 0.05$, **$p < 0.01$, ***$p < 0.001$, ****$p < 0.0001$. Data represent mean ± standard deviation (SD). *p* values reported in the figure represent ANOVA main effects. The vehicle-injected KO mice had impaired social novelty preference (spent more time with a familiar compared to a novel mouse) and reversal learning compared to WT mice. Impaired social behavior and reversal learning were normalized to WT levels in KO 3e11 but not KO 1e11 mice. No difference was observed in sociability, memory acquisition, and retention between WT, KO, and KO 1e11 or 3e11 mice.

addition, the paradigm allows detection of whether a reversal learning deficit arises because of altered sensitivity to positive reinforcement and/or because of negative reinforcement. Since we did not observe a difference between control WT and KO mice in other behavioral testing at P28–P30, we studied probabilistic learning and/or reversal learning at P60–P75. We found no genotype

difference in the number of trials to achieve the acquisition and retention criteria (Figures 7H and 7I; Table S14). However, control KO mice consistently required more trials to achieve the reversal learning criterion compared to WT mice (Figure 7J; Table S14). We next analyzed the effect of treatment on reversal learning. We found that only KO mice injected with high-dose, but not low-dose, AAV9-NG276 showed significantly improved performance in achieving the reversal learning criterion (Figure 7J; Table S14).

Together, neonatal treatment with high-dose AAV9-NG276 led to significant improvements in the behavioral performance of KO mice when tested at P60–P75, specifically, improving social novelty preference and reversal learning. Our study suggests that high-dose treatment with AAV9-NG276 early in development is sufficient to improve outcomes in adult KO mice.

DISCUSSION

AAV gene therapy has been successfully developed to treat several genetic disorders, such as spinal muscular atrophy, RPE65 mutation-associated retinal dystrophy, hemophilia A and B, and aromatic L-amino acid decarboxylase deficiency, as reviewed in Byrne et al.[58] As a monogenic disorder, FXS is a candidate for viral vector-based gene therapy, as reviewed in Hampson et al.[24] Indeed, there are multiple reports demonstrating that restoring FMRP in rodent models of FXS using different AAV serotypes, *FMR1* isoforms, promoters, and routes of administration results in full or partial rescue of functional and behavioral deficits.[21,26,28–31,59]

In this study, we explored whether an AAV gene therapy cassette that expresses human FMRP isoform 7 under the control of an endogenous *FMR1* promoter fragment and 3′ UTR elements can rescue cortical and behavioral phenotypes in KO mice. We found a significant normalization of EEG patterns in the right FC of adolescent KO mice treated with the higher dose of AAV9-NG276 tested, including (1) a reduction of enhanced baseline gamma power, (2) improved timing of the evoked responses to frequency-modulated sounds in gamma frequencies and reduced background neural activity, and (3) improved habituation, showing a dose-dependent reduction in ongoing responses elicited by broadband noise trains with 0.25 and 4 Hz repetition rates. Moreover, we observed that high-dose AAV9-NG276 can correct behavioral deficits in KO mice. Specifically, we found improvements in exploratory behavior, social novelty preference, and reversal learning in adult KO mice following neonatal treatment with AAV9-NG276 (Figure 8).

A major challenge in the treatment of FXS is that *FMR1* can undergo extensive alternative splicing to yield multiple isoforms, many of which do not have a clear role in FXS pathogenesis.[32] Previous studies have found a beneficial impact of other abundant human *FMRP* isoforms (1, 5, and 17) on similar phenotypes in FXS mice as examined in this study.[21,30] This may suggest redundancy across multiple FMRP isoforms. The current study demonstrates the strong functionality and therapeutic potential of utilizing isoform 7 of FMRP in treating FXS.

Translationally relevant EEG phenotypes in the KO model include elevated baseline gamma power,[38–43,60] decreased gamma power after perceptual experience in visual cortex (V1),[61] increased sound-evoked power, reduced habituation of sound-evoked responses, and impaired selectivity and timing of the responses.[39,40,48,50,62] An earlier study reported that i.c.v. injection of AAV9 expressing the rodent homolog of human isoform 17 driven by the mouse *Mecp2* promoter in neonatal mice rescued delta power abnormality but did not affect elevated gamma power in the adult KO mice.[29] Here, we found that high-dose AAV9-NG276 normalized high gamma power to WT levels in both the AuC and FC of KO mice, while low-dose AAV9-NG276 decreased the elevated gamma power in the FC but not the AuC of KO mice. A similar decrease of elevated gamma power (low gamma) was previously reported after developmental expression of mouse FMRP in cortical excitatory neurons of KO mice.[48] As inhibitory fast-spiking PV interneurons have been implicated in regulating gamma oscillations in the brain,[63,64] altered gamma responses in EEG recordings were suggested to reflect abnormal development and function of PV-expressing GABAergic interneurons in the KO mouse cortex.[65,66] Low PV expression and function in the KO mouse cortex have been attributed to abnormal communications between pyramidal neurons and PV cells.[67,68] Therefore, correction of abnormal gamma power with high-dose AAV9-NG276 may indicate correction of FMRP expression in PV interneurons and excitatory neurons that restore cortical communications. Indeed, we observed a significant FMRP expression in cortical neurons and PV interneurons to WT levels. This is consistent with the findings of Rais et al.,[48] who observed that early postnatal FMRP re-expression in cortical excitatory neurons restored PV levels and normalized gamma power to WT levels. In addition, high-dose AAV9-NG276 achieved higher transduction levels compared to those reported by Hooper et al.[29]

Similarly to that reported by Rais et al.,[48] we did not observe a difference in delta power or power of other frequencies between control WT and KO mice. However, a power coupling analysis revealed an abnormal low/high EEG power coupling between theta and high-gamma bands and between alpha and high-gamma bands across regions (FC to AuC) in KO mice compared to WT mice. Similar findings were previously demonstrated in young and adult KO mice.[39,69] Treatment with high-dose AAV9-NG276 restored theta/high gamma and alpha/high-gamma power coupling in KO mice to WT levels. Since we did not observe a difference in alpha and theta power between control WT and KO mice, the difference in power coupling was most likely driven by changes in gamma power.

Another finding of this work was that high-dose AAV9-NG276 improved fidelity of temporal processing and reduced background gamma power in the AuC and FC of young KO mice. As reported in previous studies, control KO mice displayed a reduced ability to synchronize to stimulus-induced oscillations in the 30–55 and 60–80 Hz ranges in the AuC and the 60–80 Hz range in the FC.[38,39] In addition, these animals exhibited enhanced background gamma power in the AuC and FC. We observed improvements in

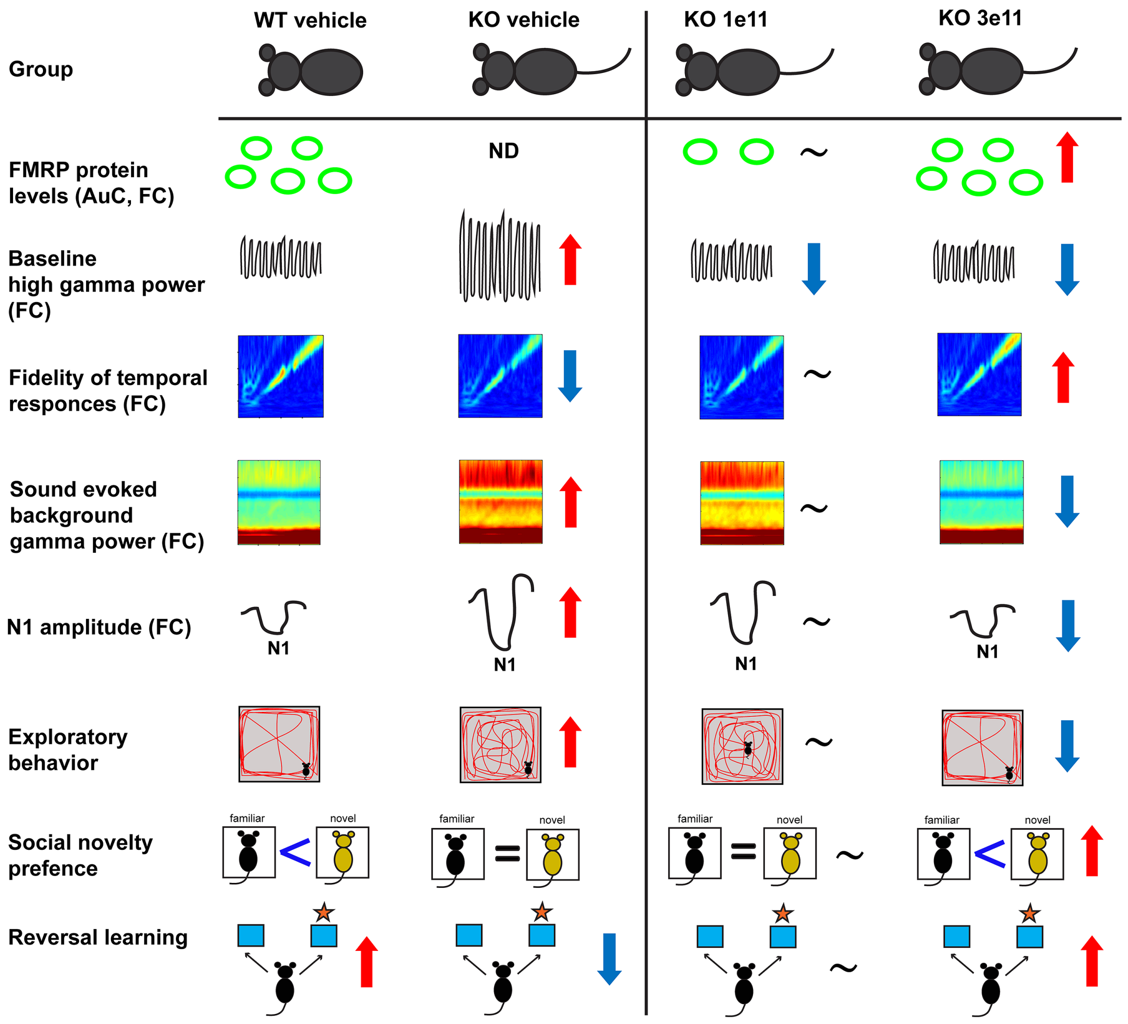

Figure 8. Summary schematic of main changes observed in KO mice after the neonatal i.c.v. AAV9-NG276 treatment

WT vehicle: vehicle-treated WT mice show normal levels of FMRP, baseline gamma power, fidelity of temporal responses to chirp, evoked background power, N1 amplitude in response to broadband noise with 0.25 Hz repetition rate in the frontal cortex, behaviors, such as exploratory activity, social novelty preference, and reversal learning. KO vehicle: vehicle-treated KO mice show (1) loss of FMRP protein, (2) increased baseline gamma power, (3) decreased fidelity of temporal responses to chirp, (4) increased sound evoked background gamma power, (5) increased N1 amplitude in response to broadband noise with a 0.25 Hz repetition rate in the frontal cortex, and (6) altered behaviors, such as exploratory activity, social novelty preference, and reversal learning compared to vehicle-treated WT mice. KO 1e11: KO mice treated with low-dose AAV9-NG276 show no changes in all measures except baseline gamma power, which was decreased in the FC compared to vehicle-treated KO mice. KO 3e11: KO mice treated with high-dose AAV9-NG276 show improvement in all measures compared to vehicle-treated KO mice. Blue arrows depict decrease and red arrows depict increase, wavy lines indicate no changes, and a horizontal line indicates absence, and ND indicates not detected.

synchronization in the 60–80 Hz range in both the AuC and FC of KO mice treated with high-dose AAV9-NG276. Similar improvements in the ability to phase-lock to spectrotemporally modulated sounds were previously reported in mice after FMRP re-expression in excitatory neurons[48] and pharmacological treatments, such as minocycline[70] and the matrix metalloproteinase-9 (MMP-9) inhibitor SB3-CT.[39] In this study, we did not see the improvement in the low-gamma range (30–55 Hz) in contrast to this deficit rescue reported by Rais et al.[48] The difference between the two studies may be explained by the fact that Rais et al.[48] recorded from P60–P70 mice with FMRP re-expression for a longer duration than that used here. Another point of consideration is that chirp inter-trial phase coherence (ITPC) may depend on both forebrain and midbrain expression levels of FMRP. Holley and colleagues showed that FMRP re-expression only in the midbrain is sufficient to improve ITPC for chirp.[71] The extent to which the gene was expressed in the current study in forebrain and midbrain can lead to differences in ITPC.

Previous studies in the adult AuC showed enhanced responses to tones[14,72] and reduced habituation of ERPs to repeated sounds in KO mice.[62] In addition, human studies reported increased STP

during auditory stimulus presentation in patients with FXS.[50] Here, we demonstrated that adolescent KO mice exhibit a significant increase in the power of onset and ongoing responses to sounds with a 0.25 or 4 Hz repetition rate in 30–100 Hz gamma range in the AuC and FC. Treatment with high-dose AAV9-NG276 ameliorated overall power in the 30–100 Hz gamma range in response to sound trains with both repetition rates in the FC and showed a similar trend in the AuC. Moreover, we found that treatment with high-dose AAV9-NG276 decreased N1 amplitude in response to both 0.25 and 4 Hz tone in the FC of P28–P30 mice. Elevated N1 amplitude was observed in the FC of control KO mice, and it was also previously reported in adult KO mice[73,74] and humans with FXS.[41,49,75,76]

Among the most common symptoms reported in FXS are deficits in cognitive and social skills that have been linked to the prefrontal cortex (PFC) and associated fronto-striatal networks.[77–80] A study showed that elimination of mouse FMRP from the PFC alone leads to dramatic deficits in learning, and restoration of FMRP rescues performance in FXS mice.[81] Another study showed that FMRP loss disrupts cortico-striatal circuitry that drives repetitive behavior in a mouse model of FXS.[82] In humans, associations between EEG spectral signatures and some behavioral parameters, such as social and cognitive skills, have been reported.[83–85] We found that human FMRP transgene expression resulted in significant EEG improvement in the FC, and high-dose AAV9-NG276 ameliorated learning deficits in KO mice. The improvements in cognitive flexibility that we observed in KO mice after the treatment with AAV9-NG276 may indicate the improvement in synaptic and neuronal functions, which are consistent with the improvements observed in EEG in the FC of the treated mice.

Impaired social interactions are among typical behavioral observations in FXS, especially during development. KO mice were also reported to display a reduced preference for novelty in social settings.[48,56] We confirmed impaired social novelty preference in adult control KO mice compared to control WT mice. High-dose AAV9-NG276 normalized social preference in mice to WT levels. Similar findings were reported by other studies.[30] Using alternative approaches to test the effects of AAV gene therapy on social interactions in KO mice, a few studies showed rescue of social dominance in KO mice after treatment.[28,29] All these findings indicate that FMRP restoration using AAV-based approaches is promising for correcting social behavior deficits in FXS.

Other common behavioral problems seen in FXS include increased anxiety and hyperactivity reviewed in Cregenzán-Royo et al. and Davidson et al.[86,87] Numerous studies showed that KO mice also exhibit multiple FXS-associated behavior alterations, such as altered exploratory behaviors, increased locomotor activity, impaired attention, increased anxiety, and repetitive behaviors.[48,53–55,88–90] Although variability in behavioral assessments of exploratory behaviors and locomotor activity was also observed, several studies showed that both young and adult KO mice display increased locomotor activity in the open field and elevated plus maze[39,48,51,54,69] and demonstrate more exploratory behaviors by spending less time in thigmotaxis and making more center entries.[51,53–55] Here, we confirmed previous findings showing increased exploratory behaviors in P60–P65 KO mice. We also demonstrated that neonatal injection of high-dose AAV9-NG276 significantly improved exploratory behaviors in KO mice. Moreover, we found a significant correlation between FMRP levels and exploratory behaviors in a low-dose KO group, where mice with high FMRP levels had a phenotype similar to WT. This finding indicates that low-dose AAV9-NG276 also has an effect in mice with >25% FMRP expression relative to WT levels.

As we did not observe a difference in locomotor activity between control KO and WT mice, we were unable to determine the effect of the AAV transgene on locomotor activity. Similar findings were reported in a previous study, where WT and KO mice did not display a difference in locomotor activity.[30] In contrast, other studies observed a genotype difference in locomotion between control WT and KO mice and the following partial or full rescue[26,29] or no rescue of locomotor activity in injected mice.[27,91] Interestingly, overexpression of FMRP was demonstrated to increase locomotion in adult mice.[26–28]

The current study focused mainly on frontal and auditory circuits and demonstrated a clear dose-response relationship between the proportion of neurons expressing the vector-derived transgene and the normalization of circuit function. This demonstrates the importance of restoring FMRP to a sufficient number of cells in the cortex to rescue the behaviors evaluated (i.e., between 50% and 70% of cortical neurons in this animal model). However, as restoration of expression across multiple central nervous system structures is likely important to address multiple FXS phenotypes, a limitation of the current study is that it did not evaluate the cell type specificity of the vector-derived FMRP expression. In addition, we did not assess molecular pathways contributing to cellular, functional, and behavioral impairments in FXS, such as MMP-9 activity,[56,66,92,93] mGluR5[94–96] and muscarinic acetylcholine receptor M4 signaling,[97,98] or the AMPA/NMDA receptor ratio.[99,100] Future studies may explore broader cell specificity, molecular mechanisms, and improvement in functional assays to elucidate the brain circuits that require correction to achieve efficacy and further establish the translational potential of this construct.

The finding that phenotypic benefit was achieved at the high dose evaluated has important implications for the translational potential of gene therapy for FXS. The human equivalent of the high dose evaluated in mice is likely to be beyond a dose that could be delivered safely to pediatric and adult subjects, limiting the feasibility of current gene therapy approaches, such as i.c.v. delivery of AAV9.[101,102] This implies that while the construct tested in this study demonstrates therapeutic benefit, FXS is a condition that requires the use of improved delivery technologies (e.g., a highly brain-penetrant AAV capsid) to transduce the number of cells across the brain required for efficacy.

Another important factor to consider is the timing of gene therapy delivery. Since the age of treatment administration has an impact on biodistribution, it is difficult to parse the impact of age of delivery vs. phenotypic correction at different developmental stages. Furthermore, the expression level of genes, including FMRP,[91] changes during development, but FMRP expression throughout life suggests that it has an ongoing function and does not rule out the potential for correction at later ages. This would require testing empirically, which is beyond the scope of this initial proof-of-concept study.

In summary, this study demonstrates that expression of human FMRP isoform 7 driven by a human *FMR1* promoter fragment can normalize frontal neurocircuit deficits that are likely to improve altered behavior phenotypes, such as exploratory behaviors, social novelty preference, and cognitive flexibility. With broad transduction of cells across the brain, this construct could provide a transformative therapy for FXS individuals.

METHODS

Ethics statement

All mouse studies were done according to the guidelines approved by the Institutional Animal Care and Use Committee at the University of California, Riverside, and were performed in accordance with NIH "Guide for the Care and Use of Laboratory Animals."

Mice

KO mice on the C57BL/6 background (JAX: 003025; Jackson Laboratories, CA, USA) and their WT counterparts (JAX: 000664; Jackson Laboratories, CA, USA) were bred as littermates (by crossing heterozygous KO females and WT or KO males). Animals were maintained in an AAALAC-accredited facility under a 12-h light/dark cycle and fed standard mouse chow. Water and food were provided to the mice *ad libitum*.

Vector description

AAV9-NG276 is a recombinant AAV9 encoding isoform 7 of the human *FMR1* (*hFMR1*) *transgene*.[103] The transgene is encoded within a single-stranded AAV genome composed of a WT AAV2 5′ inverted terminal repeat (WT AAV2 5′ ITR), a 1,050 bp fragment of the human *FMR1* promoter, the *FMR1* coding sequence for human isoform 7, a 1,400 bp fragment of the human *FMR1* 3′ untranslated region (3′ UTR) encompassing a polyadenylation signal, and a WT AAV2 3′ inverted terminal repeat (WT AAV2 3′ ITR) (Figure S1A). This promoter fragment has high levels of sequence conservation with other species, including mouse and cynomolgus macaque. The transgene is additionally regulated by endogenous elements from the *FMR1* 3′ UTR. We included a 1,400 bp fragment of the human *FMR1* 3′ UTR containing putative regulatory elements including miRNA recognition sites and the predominant polyadenylation signal. Combined, we predicted that these regulatory elements would drive *FMR1* expression to reflect the tissue and cell specificity of endogenous FMRP expression, which might lead to greater efficacy and less off-target effects than the use of strong, ubiquitous promoters.

The vector was produced by Virovek Inc. (Houston, TX) using a Baculovirus Expression Vector System using *Spodoptera frugiperda* (Sf9) insect cells and was formulated in a PBS buffer containing 0.001% Poloxamer 188. Vector titer was determined using a qualified digital droplet PCR (ddPCR) assay targeting human *FMR1* sequences.

I.c.v. injections

P1–P2 WT and KO male littermates were dosed via i.c.v. injection with 4 μL (2 μL per side). Mice received vehicle (PBS buffer containing 0.001% Poloxamer 188) or AAV9-NG276 at one of two doses: 1e11 or 3e11 vg/mouse. Each litter contained 2–5 male pups that were all injected either with buffer or virus to prevent possible infection of buffer-injected pups by viral-injected pups. For the injection, pups were individually removed from cages and placed onto a glass Petri dish pre-chilled to 4°C and then placed on ice. After approximately 5 min, the state of anesthetization was determined by the foot pinch response. If no movement occurred, pups were subjected to i.c.v. injection using a head bar to stabilize the head. The site of injection was approximately equidistant from the lambdoid suture and eye and 2 mm lateral to the sagittal suture. A Hamilton syringe needle was then inserted to the 2 mm mark, to ensure penetration into the lateral ventricle. To reduce intracranial pressure, grip was loosened on the head of the pup, and the needle was slowly removed following the injection. After each injection, the pups were placed on a heating pad for 5 min. Pups were gently rotated every 30 s with mild stimulation. Once pink and moving, pups were returned to their biological mother. The experimenter ensured that the mother attended to and nursed the injected pups within 20 min of placing them into the home cage. If the mother failed to collect the pups within this time, pups were transferred to a foster mother of the same genotype. Upon completion of the procedure, the animals were returned to the vivarium. Mice were monitored daily for any signs of pain and discomfort (including but not limited to weight loss, reduced mobility, hunching, and respiratory distress).

Quantitative polymerase chain reaction

The DNeasy Blood and Tissue Kit (QIAGEN, MD, USA) was used to extract DNA from snap-frozen tissues. Tissues were homogenized in buffer ATL + proteinase K as per manufacturer's instructions in a Bead Mill 24 (Fisher Scientific, MA, USA) for 20 s and incubated at 56°C for 1 h before following manufacturer's instructions for DNA purification. DNA concentrations were measured using a Nanodrop One (Thermo Fisher Scientific, MA, USA). Vector copy number was determined using Taqman absolute quantitative PCR (qPCR) assays targeting the 3′ end of the *FMR1* gene plus an anchor sequence in the 3′ UTR of the construct. Known copy numbers of a linearized plasmid containing the *FMR1* sequence were used as a standard. qPCR was performed using Taqman Fast Advanced Master Mix (Thermo Fisher Scientific, MA, USA) that included forward and reverse primers at final concentrations of 900 nM, probe at a final concentration of 250 nM, and 200 ng DNA per well. The following primer and probe sequences of the vector biodistribution assay were used in these studies: h*FMR1*-3′

UTR-forward primer: AAAGAGAAGCCAGACAGC; h*FMR1*-3′ UTR-reverse primer: GCCATAAGGTCATGTACTGG; h*FMR1*-3′ UTR-probe (FAM-PrimeTime chemistry, IDT): ACTCGTGAA TGGAGTACCCTGA. Assays were performed using 3–6 biological replicates (depending on group sizes) and in technical triplicates. All plates were run on a QuantStudio 7 Pro Real-Time PCR System (Applied Biosystems, CA, USA) using StepOne Software v.2.3 or Design and Analysis 2.8.0 (Applied Biosystems, CA, USA). Copy numbers per well were extrapolated from the standard curve using Microsoft Excel and visualized using GraphPad Prism 10 software (RRID: SCR_002798; GraphPad Prism, Boston, MA, USA). Data were expressed as vector genomes per ug DNA.

Immunofluorescence

Mice were euthanized with isoflurane at P28–P30 and perfused transcardially first with cold phosphate-buffered saline (PBS, 0.1 M) and then with 4% paraformaldehyde (PFA) in PBS. Brains were removed, post-fixed for 24 h in 4% PFA followed by 48 h in 30% sucrose, and 100-μm coronal sections containing the AuC and FC were obtained using a cryostat (HM500M; Microm, Rotherham, UK). Identification of the AuC was performed using hippocampal landmarks.[66] On average, 5–6 slices containing the AuC and FC were obtained from each brain. Immunolabeling of sections was performed using the protocol as described[29] with modifications. Briefly, brain slices were washed in 0.1 M PBS and then quenched in a 0.8% sodium borohydride solution (in 0.1 M PBS) for 5 min at room temperature. Following washes in PBS, antigen retrieval was performed (10 mM in citric buffer containing 0.05% Tween 20, pH 6.0) for 15 min at 75°C. After cooling down on ice, slices were washed in 0.1 M PBS. Then, brain tissues were incubated with a blocking solution containing 3% bovine serum albumin (BSA, #BP9703100; Fisher Scientific, MA, USA), 5% normal donkey serum (NDS, #017000121; Jackson ImmunoResearch Laboratories, PA, USA), and 0.2% Triton X-100 in a 0.1 M PBS solution to block tissue nonspecific staining. Sections were further incubated with primary mouse anti-FMRP antibodies (#7G1-1, 1:400, RRID: AB_528251; DSHB, IA, USA) and rabbit anti-NeuN antibodies (#ab104225, 1:1,000, RRID: AB_10711153; Abcam, MA, USA) or rabbit anti-PV antibodies (#PV27, 1:5,000, RRID: AB_2631173; SWANT, Switzerland) in blocking buffer for 24 h. Next, slices were washed in 0.1 M PBS containing 0.1% Tween (PBST) and incubated with secondary antibodies, donkey anti-mouse Alexa 488 (4 μg/mL, #A21202, RRID: AB_141607; Thermo Fisher Scientific, MA, USA) and donkey anti-rabbit Alexa 594 (4 μg/mL, #A21207, RRID: AB_141637; Thermo Fisher Scientific, MA, USA), in PBST for 2 h. Brain tissues were further washed with PBST and PBS, mounted with Vectashield (# H-1200; Vector Labs, CA, USA), and sealed with Cytoseal (#8310–16; Thermo Scientific, MA, USA). Each slice was imaged with a Zeiss 880 confocal microscope using a series of 10 high-resolution optical sections (2,048 × 2,048-pixel format) and a 20×, 0.6× zoom at 1 μm step (z stack). Image analysis was performed using ImageJ. Statistical analysis was performed using one-way ANOVA with Tukey's post hoc test and GraphPad Prism 10 software (RRID: SCR_002798; GraphPad Prism, Boston, MA, USA). A subset of samples was processed at Histowiz (NY, USA) using the Leica Bond RX automated stainer (Leica Microsystems, Germany) and fully automated workflow. First, brains were sectioned at 4 μm. The slides were dewaxed using Leica's Bond Dewax Solution (#AR9222; Leica Microsystems, Germany). Next, heat-induced epitope retrieval of the formalin-fixed, paraffin-embedded tissue was performed for 15 min at 100°C using EDTA-based solution pH 9 (#AR9640; Leica Microsystems). The tissues were incubated with 10% goat serum (#005-000-121; Jackson ImmunoResearch Laboratories, PA, USA) in 1× PBS (#10010023; Gibco, MA, USA), followed by incubation with the primary antibody cocktail for 60 min. The following primary antibodies (diluted in 10% normal goat serum) were used: mouse anti-FMRP (#2F5-1, 4 μg/mL, RRID: AB_10805421; DSHB, IA, USA) and chicken anti-NeuN (#ABN91; 1:500, RRID: AB_11205760; MilliporeSigma, MA, USA). After the washes, tissue slices were incubated with the secondary antibodies (diluted in 10% goat serum) for 60 min. The following antibodies were used: goat anti-mouse Alexa 488 (#4408, 1:500; Cell Signaling Technology, MA, USA) and goat anti-chicken Alexa 594 (#A-11044, 1:500; Invitrogen, CA, USA). After washes (using Bond Wash Solution), slides were incubated with 10× spectral DAPI (#FP1490; Akoya Biosciences, MA, USA), washed with Bond Wash Solution, dried, coverslipped (#CV5030; Leica Microsystems, Germany), and visualized using an Akoya Vectra Polaris slide scanner (Akoya Biosciences, MA, USA) at 20× magnification.

Western blotting

Mice were euthanized at P70–P75 with isoflurane, and cervical dislocation was performed. The AuC and FC were identified based on methods described earlier and dissected as previously described[36] with modifications and placed on dry ice (stored at −80°C). Tissue samples were homogenized in RIPA buffer (50 mM Tris-HCl, pH 7.4; 150 mM NaCl; 1 mM EDTA, pH 8.0; 1% Triton X-100; 0.1% SDS; protease inhibitor cocktail; 0.5 mM sodium pervanadate). Samples were rotated at 4°C for 1 h to allow for complete cell lysis and then cleared by centrifugation at 13,200 rpm for 20 min at 4°C. Samples were boiled in reducing sample buffer (Laemmli 2× concentrate, S3401; Sigma, MA, USA) and separated on 8%–16% Tris-glycine SDS-PAGE precast gels (Invitrogen, CA, USA). Proteins were transferred onto Protran BA 85 nitrocellulose membrane (GE Healthcare, IL, USA), washed two times (5 min each) with Milli-Q water, and further blocked for 1 h at room temperature in 5% skim milk (#170-6404; Bio-Rad, CA, USA). Incubation with primary antibody diluted in TBS/0.1% Tween 20/5% BSA was performed overnight at 4°C. The following primary antibodies for protein detection were used: rabbit anti-FMRP (1:1,000, #F4055, RRID: AB_1840858; Sigma, MA, USA) and mouse anti-β-actin (1:1,000, #sc-47778, RRID: AB_626632; Santa Cruz, TX, USA). Blots were washed three times for 10 min with TBS/0.1% Tween 20 and incubated with the appropriate HRP-conjugated secondary antibodies for 2 h at room temperature in a TBS/0.1% Tween 20/5% BSA solution. The secondary antibodies used were anti-mouse-HRP at 1:5,000 (#715-035-150, RRID: AB_2340770; Jackson ImmunoResearch Laboratories, PA, USA) and anti-rabbit-HRP at 1:5,000 (#111-035-003,

RRID: AB_2313567; Jackson ImmunoResearch Laboratories, PA, USA) post-anti-FMRP and anti-β-actin treatment, respectively. After secondary antibody incubations, blots were washed three times for 10 min in TBS/0.1% Tween 20, incubated in ECL 2 western blotting substrate (#32106; Thermo Scientific, MA, USA), and imaged using iBright imaging system (RRID: SCR_026565; Thermo Fisher Scientific, MA, USA). For re-probing, membrane blots were washed in stripping buffer (2% SDS, 100 mM β-mercaptoethanol, 50 mM Tris-HCl, pH 6.8) for 30 min at 55°C, then rinsed repeatedly with TBS/0.1% Tween 20 (5 × 5 min), blocked with 5% skim milk, and then re-probed. Band density was analyzed by measuring band and background intensity using Adobe Photoshop software (RRID: SCR_014199; San Jose, CA, USA). Samples from different treatment groups (WT buffer, KO buffer, KO 1e11 and KO 3e11) were run on the same blot, and precision/tolerance (P/T) ratios for KO samples were normalized to averaged P/T ratios of WT samples. A subset of samples including hippocampus, striatum, thalamus, cerebellum, and whole cortex (P28–P30 and P70–P75) were processed at NeuroLink (Houston, TX) according to the following protocol. First, samples were added to sterile tubes pre-filled with ceramic beads and homogenized in 300 μL NE1 buffer (20 mM HEPES, 10 mM KCl, 1 mM MgCl2, 0.1% Triton X-100, 20% glycerol, 0.5 mM DTT, 1× Pierce Complete Protease Inhibitors) using a Bead Mill 24 homogenizer (Fisher Scientific, MA, USA). Samples were chilled on ice; 1 μL benzonase (#E1014-5KU; Sigma, MA, USA) was added per tube, briefly mixed, and allowed to incubate at room temperature for 15 min. A fraction of the homogenate was diluted in NE1 buffer, and protein concentrations were quantified using BioRad DC II (Bio-Rad, CA, USA) on an ID3 plate reader (Molecular Devices, CA, USA). 100 μL of 4× Laemmli buffer was added to each sample, and the sample was boiled at 100°C for 10 min before being stored at ≤−60°C. Protein concentrations were extrapolated in Microsoft Excel, and volumes for loading 10–20 μg of protein per lane were determined, taking into account dilution factors and the addition of 1/3rd volume of 4× Laemmli buffer. Samples were further resolved on a pre-cast 4%–20% acrylamide gel until the ladder (#1610374, Dual Precision Plus; Bio-Rad, CA, USA) was fully resolved. Proteins were transferred to nitrocellulose membranes for 2 h at 85 V at 4°C. After transfer, total protein values were collected using the Revert 700 Total Protein kit (#926-11016; LI-COR, NE, USA). Dye was removed and membranes were blocked in Intercept TBS Blocking Buffer (#926–60003; LI-COR, NE, USA) for 1 h at room temperature. Rabbit anti-FMRP antibody (1:1,000, #ab17722, RRID: AB_2278530; Abcam, Cambridge, United Kingdom) was diluted in Intercept TBS blocking buffer, and membranes were incubated overnight at 4°C with gentle rocking. Primary antibody was removed by washing 3× 10 min in excess TBS-T. Secondary antibody (donkey anti-rabbit CW800, 1:10,000, #926-32213, RRID: AB_621848; LI-COR, NE, USA) was diluted in Intercept TBS blocking buffer and incubated for 1 h at room temperature with gentle rocking. After three times washing in excess TBS-T, membranes were rinsed in TBS and scanned on the Odyssey Classic Licor (LI-COR, NE, USA). Relative fluorescence unit (RFU) data were captured using the Empiria Studio Software (LI-COR, NE, USA) by drawing boxes around areas of fluorescence for both total protein (700 nm wavelength signal over the whole lane) and FMRP (800 nm wavelength signal at ∼75 kDa; all isoforms included in quantification). Data were transferred to Microsoft Excel where total protein RFU was used to normalize FMRP RFU for the same lane. Average normalized FMRP signal was used to normalize data from each gel, and finally the average of vehicle-treated WT was used to normalize the entire dataset. A one-way ANOVA with Tukey's post hoc test was used for the analysis. Statistical analysis was performed using GraphPad Prism 10 software (RRID: SCR_002798; GraphPad Prism, Boston, MA, USA).

EEG study

Surgery for in vivo *EEG recordings*

Three weeks following neonatal vector delivery, surgical procedures were performed on P20–P22 male mice as described previously[37,39,40,70,104] with modifications. Mice were anesthetized with isoflurane inhalation (0.2%–0.5%) and an injection of ketamine and xylazine (K/X) (intraperitoneal [i.p.], 80/10 mg/kg) and then secured in a bite bar and placed in a stereotaxic apparatus (model 930; Kopf, CA, USA). Ethiqa XR (buprenorphine extended-release injectable suspension; 3.25 mg/kg body weight; Fidelis pharma Inc., NJ, USA) and meloxicam (non-steroid anti-inflammatory drug; 5 mg/kg; Covetrus, ME, USA) were injected subcutaneously. Both drugs were administered before the surgery to assure that there was an adequate therapeutic drug level present post-surgically. Artificial tear gel (Puralube Vet ointment, Dechra, KS, USA) was applied to the eyes to prevent drying. Toe pinch reflex was used to measure anesthetic state every 10 min throughout the surgery, and supplemental doses of K/X were administered as needed. Once the mouse was anesthetized, a midline sagittal incision was made along the scalp to expose the skull. A Foredom dental drill was used to drill 1 mm diameter holes in the skull overlying the right AuC (−1.6 mm, +4.0 mm), right FC (+2.6 mm, +1.0 mm), and left occipital (−3.5 mm, −5.2 mm) (coordinate relative to bregma: anterior/posterior, medial/lateral). Three channel electrode posts from Plastics One (#MS333-2-A-SPC; Protech International, TX, USA) were attached to 1 mm stainless steel screws from Plastics One (#8L003905201F; Protech International, TX, USA), and screws were advanced into drilled holes until secure. Special care was taken not to advance the screws beyond the point of contact with the dura. Dental cement was applied around the screws, on the base of the post, and exposed skull. Triple antibiotic was applied along the edges of the dental cement. Mice were placed on a heating pad to aid recovery from anesthesia. A second meloxicam injection was administered between 12 and 24 h after the surgery (and 48 h after the surgery if needed). Mice were group housed, returned to the vivarium, and monitored daily until the day of EEG recordings, which allowed 4–5 days recovery post-surgery before recording.

EEG recordings

All EEG recordings were obtained on P28–P30 mice using the BioPac system (BIOPAC Systems, Inc.) from awake and freely moving mice as published previously.[37,39,40,70,104,105] Mice were

connected to the BioPac system through a three-channel tether under brief isoflurane anesthesia and placed inside a grounded Faraday cage, inside an anechoic foam-lined sound-attenuating room (Gretch-Ken Industries, OR, USA) after recovery from isoflurane. The tether was connected to a commutator located directly above the cage. Mice were then allowed to habituate while being connected to the tether for 20 min before EEG recordings were obtained.

The BIOPAC MP150 acquisition system was connected to two EEG 100C amplifier units (one for each channel) to which the commutator was attached. The lead to the occipital cortex was used as reference for both AuC and FC electrodes. The acquisition hardware was set to high-pass (>0.5 Hz) and low-pass (<−100 Hz) filters. EEG data were collected with the gain maintained at the same level (10,000×) between all recordings. Data were sampled at a rate of either 2.5 or 5 kHz using Acqknowledge software and down-sampled to 1,024 Hz post hoc using Analyzer 2.1 (Brain Vision Inc., NC, USA). Sound delivery was synchronized with EEG recording using a transistor-transistor logic (TTL) pulse to mark the onset of each sound in a train. Baseline (no auditory stimuli) EEGs were recorded for 5 min, followed by recordings in response to auditory stimulation. After these experiments were completed, mice were returned to the colony and used for tissue dissection on a later date.

Acoustic stimulation

All experiments were conducted in a sound-attenuated chamber lined with anechoic foam (Gretch-Ken Industries, OR, USA) as previously described.[37,39,40,48,70,104,105] Acoustic stimuli were generated using RVPDX software and RZ6 hardware (Tucker-Davis Technologies, FL, USA) and presented through a free-field speaker (MF1 Multi-Field Magnetic Speaker; Tucker-Davis Technologies, FL, USA) located 12 inches directly above the cage. Sound pressure level (SPL) was modified using programmable attenuators in the RZ6 system. The speaker output was ∼65–70 dB SPL at the floor of the recording chamber.

We used acoustic stimulation paradigms that have been previously established in KO mice,[38] which is analogous to work in humans with FXS.[42] A chirp-modulated signal (henceforth, "chirp") to induce synchronized oscillations in cortical responses was used to measure temporal response fidelity. The chirp is a 2 s broadband noise stimulus with amplitude modulated (100% modulation depth) by a sinusoid whose frequencies increase (up-chirp) or decrease (down-chirp) linearly in the 1–100 Hz range.[106–108] The chirp facilitates a rapid measurement of transient oscillatory response (delta to gamma frequency range) to auditory stimuli of varying frequencies and can be used to compare oscillatory responses in different groups in clinical and preclinical settings.[108] Inter-trial coherence analysis[109] can then be used to determine the ability of neural generators to synchronize oscillations to the frequencies present in the stimulus.

To avoid onset responses contaminating phase locking to the amplitude modulation of the chirp, the stimulus was ramped in sound level from 0% to 100% over 1 s (rise time), which then smoothly transitioned into chirp modulation of the noise. Up-chirp trains were presented 300 times each (for a total of 600 trains).

To study evoked response amplitudes and habituation, trains of 100 ms broadband noise were presented at two repetition rates, 0.25 Hz (a non-habituating rate) and 4 Hz (a strongly habituating rate).[62] Each train consisted of 10 noise bursts, and the inter-train interval was 8 s. Each repetition rate was presented 100 times in an alternating pattern.[62] The onset of trains and individual noise bursts were tracked with separate TTL pulses that were used to quantify the latency of response.

EEG data analysis

Data were extracted from AcqKnowledge (BIOPAC Systems, CA, USA) and saved in a file format (EDF) compatible with BrainVision Analyzer 2.1 software as previously described.[39,70,104] All data were notch filtered at 60 Hz to remove residual line frequency power from recordings. EEG artifacts were removed using a semi-automatic procedure in Analyzer 2.1 for all recordings. Less than 30% of data were rejected due to artifacts from any single mouse. If more than 30% of data were rejected, the animal was excluded from the analysis. Baseline EEG data were divided into 2-s segments, and fast Fourier transforms (FFTs) were calculated on each segment using 0.5 Hz bins and then average power ($\mu V^2/Hz$) was calculated for each mouse from 1 to 100 Hz. Power was then binned into standard frequency bands: delta (1–4 Hz), theta (4–10 Hz), alpha (10–13 Hz), beta (13–30 Hz), low gamma (30–55 Hz), and high gamma (65–100 Hz). Responses to chirp trains were analyzed using Morlet wavelet analysis. Chirp trains were segmented into windows from 500 ms before chirp onset to 500 ms after the end of the chirp sound (total of 3 s because each chirp was 2 s in duration). EEG traces were processed with Morlet wavelets from 1 to 100 Hz using complex number output (voltage density, μV/Hz) for ITPC calculations and using power density ($\mu V^2/Hz$) for non-phase-locked STP. Wavelets were run with a Morlet parameter of 10 as this gave the best frequency/power discrimination. This parameter was chosen since studies in humans found the most robust difference around 40 Hz, where this parameter is centered.[42] To measure phase synchronization at each frequency across trials, ITPC was calculated. The equation used to calculate ITPC is

$$ITPC(f,t) = \frac{1}{n}\sum_{k=1}^{n}\frac{F_k(f,t)}{|F_k(f,t)|}, \quad \text{(Equation 1)}$$

where f is the frequency, t is the time point, and k is the trial number. Thus, $F_k(f,t)$ refers to the complex wavelet coefficient at a given frequency and time for the kth trial. There were no less than 225 trials (out of 300) for any given mouse after segments containing artifacts were rejected.

EEG statistical analysis

All statistical analysis was performed as described previously.[37–40,48,70,104,105] Statistical group comparisons of chirp responses (ITPC and STP) and broadband noise trains (ITPC and

STP) were quantified using MATLAB (RRID: SCR_001622; MathWorks, Natick, MA, USA). The analysis was conducted by binning time into 256 parts and frequency into 100 parts, resulting in a 100×256 matrix. Non-parametric cluster analysis was used to determine contiguous regions in the matrix that were significantly different from a distribution of 1,000 randomized Monte Carlo permutations.[110] Briefly, if the cluster sizes of the real genotype assignments (both positive and negative direction, resulting in a two-tailed alpha of $p = 0.025$) were larger than 97.25% of the random group assignments, those clusters were considered significantly different between genotypes. This method avoids statistical assumptions about the data and corrects for multiple comparisons.

Behavior assessment

Open field test

Exploratory-like behaviors and locomotor activity were tested in P28–P30 and P60–P65 mice as described previously.[37,48,51,104,105] The cages with mice were transferred to the behavioral room 30 min before the testing. A 72×72 cm open field arena with 50-cm-high walls was constructed from opaque acrylic sheets with a clear acrylic sheet for the bottom. The open field arena was placed in a brightly lit room (304 lux), and one mouse at a time was placed in a corner of the open field and allowed to explore for 10 min while being recorded with digital video from above. The floor was cleaned with 2%–3% acetic acid, 70% ethanol, and water between tests to eliminate odor trails. The mice were tested between the hours of 8:00 a.m. and 2:00 p.m., and this test was always performed before the 3-chamber test. The arena was subdivided into a 4×4 grid of squares with the middle of the grid defined as the center. A line 4 cm from each wall was added to measure thigmotaxis. Locomotor activity was scored by total distance traveled and velocity using TopScan Lite software (Clever Sys., Inc., VA, USA). A tendency to travel to the center (crosses through the center), time spent in the center of the open field (arena without thigmotaxis), and the time in thigmotaxis were used as an indicator of exploratory behaviors. The analysis was performed for the first and second 5 min and total 10 min period of testing. Assessments of the digital recordings were performed blind to the condition. Data represent mean ± standard deviation (SD).

3-Chamber test

Sociability and social memory were studied at P28–P30 and P60–65 using a three-chamber test as described previously.[37,56,111,112] Briefly, a rectangular box contained three adjacent chambers, 19×45 cm each, with 30-cm-high walls and a bottom constructed from clear Plexiglas. The three chambers were separated by dividing walls, which were made from clear Plexiglas with openings between the middle chamber and each side chamber. Removable doors over these openings permitted chamber isolation or free access to all chambers. All testing was done in a brightly lit room (304 lux), between 9:00 a.m. and 4:00 p.m. The test mouse was placed in the central chamber with no access to the left and right chambers and allowed to habituate to the test chamber for 5 min before testing began. Session 1 measured sociability. In session 1, another mouse (stranger 1) was placed in a wire cup-like container in one of the side chambers. The opposite side had an empty cup of the same design. The doors between the chambers were removed, and the test mouse was allowed to explore all three chambers freely for 10 min, while being digitally recorded from above. The following parameters were monitored: the duration of direct contact between the test mouse and either the stranger mouse or empty cup and the duration of time spent in each chamber. Session 2 measured social memory and social novelty preference. In session 2, a new mouse (stranger 2) was placed in the empty wire cup in the second side chamber. Stranger 1, a now familiar mouse, remained in the first side chamber. The test mouse was allowed to freely explore all three chambers for another 10 min, while being recorded, and the same parameters were monitored. Placement of stranger 1 in the left or right side of the chamber was randomly altered between trials. The floor of the chamber was cleaned with 2%–3% acetic acid, 70% ethanol, and water between tests to eliminate odor trails. Assessments of the digital recordings were done using TopScan Lite software (Clever Sys., Inc., VA). To measure changes in sociability and social memory, percent time spent in each chamber was calculated in each test. Further, the sociability index and social novelty preference index were calculated as described previously[112,113]:

$$\text{sociability index} = \left(\frac{\textit{time in S1 chamber}}{\textit{time in S1 chamber+time in empty chamber}}\right), \quad \text{(Equation 2)}$$

$$\text{social novelty preference index} = \left(\frac{\textit{time in S2 chamber}}{\textit{time in S2 chamber+time in S1 chamber}}\right). \quad \text{(Equation 3)}$$

For the sociability index, values <0.5 indicate more time spent in the empty chamber, >0.5 indicate more time spent in the chamber containing stranger 1, and 0.5 indicates no preference. For the social novelty preference index, values <0.5 indicate more time spent in the chamber containing stranger 1 or a now familiar mouse, >0.5 indicate more time spent in the chamber containing stranger 2 or a new stranger mouse, and 0.5 indicates no preference. Statistical analysis was performed using two-way ANOVA followed by Fisher's LSD post hoc test to determine the effects of location preference and treatment or one-way ANOVA using GraphPad Prism 10 software (RRID: SCR_002798; GraphPad Prism, Boston, MA, USA). Data represent mean ± SD.

Probabilistic reversal learning test

Reversal learning tasks are used in mice to assess cognitive-behavioral flexibility. The test was performed after the open field and the 3-chamber tests were completed, starting at P61–P65. One week before training, P61–P65 animals were placed on a food restriction diet to reduce body weight to 85% of baseline. The test consisted of four phases: spatial discrimination training, acquisition training, retention testing, and reversal learning.

Spatial discrimination training (day 1–2): two food wells were baited with a 1/2 piece of a food reward (fruity pebble). Mice were placed in the holding chamber first, after which they were allowed to enter the choice chamber. Mice were allowed to consume the food pebble from within each food well, after which the door was immediately raised to allow the mouse to return to the holding chamber. If the mouse failed to exit the door and return to the start area, the experimenter would nudge it gently back to the holding chamber. By the last session of training, all mice returned to the start area within 3–5 s after consuming both food wells. Once a mouse had completed 4–6 trials in 15 min for 2 consecutive days, acquisition training would begin on the following day.

Acquisition training (day 3): the food bait was placed in the "correct location 1" in 80% of trials and in the "incorrect location 2" in 20% of trials. The location of 1 and 2 was counterbalanced within and across groups. Mice were placed in the holding chamber first, after which the door opened to allow the mouse to enter the choice area where they would choose to enter one of the two spatial locations. The choice of correct location 1 would yield a food reward in 80% of the trials. When the well was not baited, the food bait was removed from location 2. Once the mouse finished a pebble, the door was raised immediately to allow it to return to the holding chamber. If it chose the incorrect location 2, it would be presented with a cereal pebble in 20% of trials. The choice area was cleaned with 2% ammonium chloride solution to minimize the use of odor cue. Mice would complete the acquisition following 6 consecutive correct trials.

Retention test (conducted just prior to reversal learning) (day 4): during the retention test, six trials were repeated similar to the acquisition phase. If a mouse made the correct choice in five out of six trials, it would progress to the reversal learning session.

Reversal learning (day 4): the food bait was placed in the "incorrect spatial location 2" now considered the "correct choice" in 80% of trials and in correct location 1 now considered the "incorrect choice" in 20% of the trials (i.e., the 80/20 probabilities of receiving a food reward would switch between chambers from what was initially learned). Mice were placed in the holding chamber, after which the door was opened to allow the mouse to enter the choice area. Mice would have a choice of entering one of the two spatial locations. Choosing a correct choice or location 2 would introduce the cereal pebble in 80% of trials. When the well was not baited, the food bait would be removed from location 2. After the mouse consumed the food reward, the door was raised immediately to allow the mouse to return to the holding chamber. If the mouse chose an incorrect choice or location 1, it was presented with the cereal pebble in 20% of trials. The choice area was cleaned with 2% ammonium chloride solution to minimize the use of odor cue. Each mouse completed the reversal learning phase when it made 6 consecutive correct trials. The number of trials to mastery was recorded for each mouse as a measure of behavioral flexibility.

DATA AVAILABILITY

The data generated and analyzed in the current study will be available upon request.

ACKNOWLEDGMENTS

This work was supported by a sponsored research agreement from Neurogene. The development of the gene therapy construct was supported by the Simons Initiative for the Developing Brain. Work in Ethell laboratory was also supported by a grant from the National Institute of Neurological Disorders and Stroke (NS129555 to I.M.E.) and a FRAXA Research Foundation fellowship (to A.O.N.). We thank Cirila Arteaga for technical support with the mouse colony; members of the Ethell, Razak, and Goel laboratories for helpful discussions; and Dr. David Carter for advice on confocal microscopy.

AUTHOR CONTRIBUTIONS

A.O.N.: experiments, data analysis, and manuscript writing/editing; C.S.: experiments, data analysis, and manuscript review/editing; D.B.: conceptualization and manuscript review/editing; R.D.H.: vector design and creation and manuscript review/editing; A.V.: experiments and data analysis; A.S.: experiments and data analysis; N.F.: data analysis; S.R.B.: conceptualization and manuscript review/editing; J.B.: experiments, data analysis, and manuscript review/editing; K.A.R.: conceptualization, supervision, and manuscript review/editing; J.S.: conceptualization and manuscript review/editing; S.C.: conceptualization and manuscript review/editing; I.M.E.: conceptualization, data analysis, supervision, and manuscript review/editing.

DECLARATION OF INTERESTS

D.B., J.B., S.R.B., and S.C. are employees at Neurogene Inc.

SUPPLEMENTAL INFORMATION

Supplemental information can be found online at https://doi.org/10.1016/j.omtn.2026.102981.

REFERENCES

1. Crawford, D.C., Acuña, J.M., and Sherman, S.L. (2001). FMR1 and the fragile X syndrome: human genome epidemiology review. Genet. Med. *3*, 359–371. https://doi.org/10.1097/00125817-200109000-00006.

2. Hagerman, R.J. (1987). Fragile X syndrome. Curr. Probl. Pediatr. *17*, 626–674. https://doi.org/10.1016/0045-9380(87)90011-9.

3. Kaplan, A., Malik, S., Persaud, N.A., and Ganti, L. (2025). Exploring the relationship between Fragile X Syndrome and autism: a bibliometric analysis of global research trends. Cureus *17*, e88159. https://doi.org/10.7759/cureus.88159.

4. Pieretti, M., Zhang, F., Fu, Y.H., Warren, S.T., Oostra, B.A., Caskey, C.T., and Nelson, D.L. (1991). Absence of expression of the FMR-1 gene in fragile X syndrome. Cell *66*, 817–822. https://doi.org/10.1016/0092-8674(91)90125-i.

5. Verkerk, A.J.M.H., Pieretti, M., Sutcliffe, J.S., Fu, Y.H., Kuhl, D.P.A., Pizzuti, A., Reiner, O., Richards, S., Victoria, M.F., Zhang, F., et al. (1991). Identification of a gene (FMR-1) containing a CGG repeat coincident with a breakpoint cluster region exhibiting length variation in fragile X syndrome. Cell *65*, 905–914. https://doi.org/10.1016/0092-8674(91)90397-h.

6. Pfeiffer, B.E., and Huber, K.M. (2007). Fragile X mental retardation protein induces synapse loss through acute postsynaptic translational regulation. J. Neurosci. *27*, 3120–3130. https://doi.org/10.1523/JNEUROSCI.0054-07.2007.

7. Wang, X., Sela-Donenfeld, D., and Wang, Y. (2023). Axonal and presynaptic FMRP: Localization, signal, and functional implications. Hear. Res. *430*, 108720. https://doi.org/10.1016/j.heares.2023.108720.

8. Wang, X., Zorio, D.A.R., Schecterson, L., Lu, Y., and Wang, Y. (2018). Postsynaptic FMRP regulates synaptogenesis in vivo in the developing cochlear nucleus. J. Neurosci. *38*, 6445–6460. https://doi.org/10.1523/JNEUROSCI.0665-18.2018.

9. Zang, T., Maksimova, M.A., Cowan, C.W., Bassel-Duby, R., Olson, E.N., and Huber, K.M. (2013). Postsynaptic FMRP bidirectionally regulates excitatory synapses as a function of developmental age and MEF2 activity. Mol. Cell. Neurosci. *56*, 39–49. https://doi.org/10.1016/j.mcn.2013.03.002.

10. Contractor, A., Klyachko, V.A., and Portera-Cailliau, C. (2015). Altered neuronal and circuit excitability in Fragile X Syndrome. Neuron *87*, 699–715. https://doi.org/10.1016/j.neuron.2015.06.017.

11. Sinclair, D., Oranje, B., Razak, K.A., Siegel, S.J., and Schmid, S. (2017). Sensory processing in autism spectrum disorders and Fragile X syndrome-From the clinic to animal models. Neurosci. Biobehav. Rev. *76*, 235–253. https://doi.org/10.1016/j.neubiorev.2016.05.029.

12. Desai, N.S., Casimiro, T.M., Gruber, S.M., and Vanderklish, P.W. (2006). Early postnatal plasticity in neocortex of Fmr1 knockout mice. J. Neurophysiol. *96*, 1734–1745. https://doi.org/10.1152/jn.00221.2006.

13. Frankland, P.W., Wang, Y., Rosner, B., Shimizu, T., Balleine, B.W., Dykens, E.M., Ornitz, E.M., and Silva, A.J. (2004). Sensorimotor gating abnormalities in young males with fragile X syndrome and Fmr1-knockout mice. Mol. Psychiatry *9*, 417–425. https://doi.org/10.1038/sj.mp.4001432.

14. Rotschafer, S.E., and Razak, K.A. (2014). Auditory processing in fragile x syndrome. Front. Cell. Neurosci. *8*, 19. https://doi.org/10.3389/fncel.2014.00019.

15. Schneider, A., Leigh, M.J., Adams, P., Nanakul, R., Chechi, T., Olichney, J., Hagerman, R., and Hessl, D. (2013). Electrocortical changes associated with minocycline treatment in fragile X syndrome. J. Psychopharmacol. *27*, 956–963. https://doi.org/10.1177/0269881113494105.

16. Van der Molen, M.J.W., Van der Molen, M.W., Ridderinkhof, K.R., Hamel, B.C.J., Curfs, L.M.G., and Ramakers, G.J.A. (2012). Auditory and visual cortical activity during selective attention in fragile X syndrome: a cascade of processing deficiencies. Clin. Neurophysiol. *123*, 720–729. https://doi.org/10.1016/j.clinph.2011.08.023.

17. Van der Molen, M.J.W., Van der Molen, M.W., Ridderinkhof, K.R., Hamel, B.C.J., Curfs, L.M.G., and Ramakers, G.J.A. (2012). Auditory change detection in fragile X syndrome males: a brain potential study. Clin. Neurophysiol. *123*, 1309–1318. https://doi.org/10.1016/j.clinph.2011.11.039.

18. Bailey, D.B., Raspa, M., Olmsted, M., and Holiday, D.B. (2008). Co-occurring conditions associated with FMR1 gene variations: findings from a national parent survey. Am. J. Med. Genet. *146A*, 2060–2069. https://doi.org/10.1002/ajmg.a.32439.

19. Kau, A.S.M., Tierney, E., Bukelis, I., Stump, M.H., Kates, W.R., Trescher, W.H., and Kaufmann, W.E. (2004). Social behavior profile in young males with fragile X syndrome: characteristics and specificity. Am. J. Med. Genet. *126A*, 9–17. https://doi.org/10.1002/ajmg.a.20218.

20. Musumeci, S.A., Hagerman, R.J., Ferri, R., Bosco, P., Bernardina, B.D., Tassinari, C.A., De Sarro, G.B., and Elia, M. (1999). Epilepsy and EEG findings in males with fragile X syndrome. Epilepsia *40*, 1092–1099. https://doi.org/10.1111/j.1528-1157.1999.tb00824.x.

21. Wong, H., Hooper, A.W.M., Kang, H.R., Lee, S.J., Zhao, J., Sadhu, C., Rawat, S., Gray, S.J., and Hampson, D.R. (2023). CNS-dominant human FMRP isoform rescues seizures, fear, and sleep abnormalities in Fmr1-KO mice. JCI Insight *8*, e169650. https://doi.org/10.1172/jci.insight.169650.

22. Berry-Kravis, E., Sumis, A., Hervey, C., and Mathur, S. (2012). Clinic-based retrospective analysis of psychopharmacology for behavior in fragile x syndrome. Int. J. Pediatr. *2012*, 1–11. https://doi.org/10.1155/2012/843016.

23. Hagerman, R.J., and Polussa, J. (2015). Treatment of the psychiatric problems associated with fragile X syndrome. Curr. Opin. Psychiatry *28*, 107–112. https://doi.org/10.1097/YCO.0000000000000131.

24. Hampson, D.R., Hooper, A.W.M., and Niibori, Y. (2019). The application of adeno-associated viral vector gene therapy to the treatment of Fragile X Syndrome. Brain Sci. *9*, 32. https://doi.org/10.3390/brainsci9020032.

25. Velinov, M. (2025). Gene therapy for Fragile X Syndrome, challenges, and promises. J. Gene Med. *27*, e70049. https://doi.org/10.1002/jgm.70049.

26. Arsenault, J., Gholizadeh, S., Niibori, Y., Pacey, L.K., Halder, S.K., Koxhioni, E., Konno, A., Hirai, H., and Hampson, D.R. (2016). FMRP expression levels in mouse central nervous system neurons determine behavioral phenotype. Hum. Gene Ther. *27*, 982–996. https://doi.org/10.1089/hum.2016.090.

27. Chadman, K.K., Adayev, T., Udayan, A., Ahmed, R., Dai, C.-L., Goodman, J.H., Meeker, H., Dolzhanskaya, N., and Velinov, M. (2023). Efficient delivery of FMR1 across the blood brain barrier using AAVphp construct in adult FMR1 KO mice suggests the feasibility of gene therapy for Fragile X Syndrome. Genes *14*, 505. https://doi.org/10.3390/genes14020505.

28. Gholizadeh, S., Arsenault, J., Xuan, I.C.Y., Pacey, L.K., and Hampson, D.R. (2014). Reduced phenotypic severity following adeno-associated virus-mediated Fmr1 gene delivery in fragile X mice. Neuropsychopharmacology *39*, 3100–3111. https://doi.org/10.1038/npp.2014.167.

29. Hooper, A.W.M., Wong, H., Niibori, Y., Abdoli, R., Karumuthil-Melethil, S., Qiao, C., Danos, O., Bruder, J.T., and Hampson, D.R. (2021). Gene therapy using an ortholog of human fragile X mental retardation protein partially rescues behavioral abnormalities and EEG activity. Mol. Ther. Methods Clin. Dev. *22*, 196–209. https://doi.org/10.1016/j.omtm.2021.06.013.

30. Jiang, Y., Han, L., Meng, J., Wang, Z., Zhou, Y., Yuan, H., Xu, H., Zhang, X., Zhao, Y., Lu, J., et al. (2022). Gene therapy using human FMRP isoforms driven by the human FMR1 promoter rescues fragile X syndrome mouse deficits. Mol. Ther. Methods Clin. Dev. *27*, 246–258. https://doi.org/10.1016/j.omtm.2022.10.002.

31. Zeier, Z., Kumar, A., Bodhinathan, K., Feller, J.A., Foster, T.C., and Bloom, D.C. (2009). Fragile X mental retardation protein replacement restores hippocampal synaptic function in a mouse model of fragile X syndrome. Gene Ther. *16*, 1122–1129. https://doi.org/10.1038/gt.2009.83.

32. Fu, X., Zheng, D., Liao, J., Li, Q., Lin, Y., Zhang, D., Yan, A., and Lan, F. (2015). Alternatively spliced products lacking exon 12 dominate the expression of fragile X mental retardation 1 gene in human tissues. Mol. Med. Rep. *12*, 1957–1962. https://doi.org/10.3892/mmr.2015.3574.

33. Brackett, D.M., Qing, F., Amieux, P.S., Sellers, D.L., Horner, P.J., and Morris, D.R. (2013). FMR1 transcript isoforms: association with polyribosomes; regional and developmental expression in mouse brain. PLoS One *8*, e58296. https://doi.org/10.1371/journal.pone.0058296.

34. Denman, R.B., and Sung, Y.J. (2002). Species-specific and isoform-specific RNA binding of human and mouse fragile X mental retardation proteins. Biochem. Biophys. Res. Commun. *292*, 1063–1069. https://doi.org/10.1006/bbrc.2002.6768.

35. Xie, W., Dolzhanskaya, N., LaFauci, G., Dobkin, C., and Denman, R.B. (2009). Tissue and developmental regulation of fragile X mental retardation 1 exon 12 and 15 isoforms. Neurobiol. Dis. *35*, 52–62. https://doi.org/10.1016/j.nbd.2009.03.015.

36. Kulinich, A.O., Reinhard, S.M., Rais, M., Lovelace, J.W., Scott, V., Binder, D.K., Razak, K.A., and Ethell, I.M. (2020). Beneficial effects of sound exposure on auditory cortex development in a mouse model of Fragile X Syndrome. Neurobiol. Dis. *134*, 104622. https://doi.org/10.1016/j.nbd.2019.104622.

37. Norman, A.O., Farooq, N., Sahni, A., Tapia, K., Breiner, D., Razak, K.A., and Ethell, I.M. (2025). Differential effects of sound repetition rate on auditory cortex development and behavior in fragile X syndrome mouse model. Exp. Neurol. *387*, 115184. https://doi.org/10.1016/j.expneurol.2025.115184.

38. Lovelace, J.W., Ethell, I.M., Binder, D.K., and Razak, K.A. (2018). Translation-relevant EEG phenotypes in a mouse model of Fragile X Syndrome. Neurobiol. Dis. *115*, 39–48. https://doi.org/10.1016/j.nbd.2018.03.012.

39. Pirbhoy, P.S., Rais, M., Lovelace, J.W., Woodard, W., Razak, K.A., Binder, D.K., and Ethell, I.M. (2020). Acute pharmacological inhibition of matrix metalloproteinase-9 activity during development restores perineuronal net formation and normalizes auditory processing in Fmr1 KO mice. J. Neurochem. *155*, 538–558. https://doi.org/10.1111/jnc.15037.

40. Wen, T.H., Lovelace, J.W., Ethell, I.M., Binder, D.K., and Razak, K.A. (2019). Developmental changes in EEG phenotypes in a mouse model of Fragile X Syndrome. Neuroscience *398*, 126–143. https://doi.org/10.1016/j.neuroscience.2018.11.047.

41. Ethridge, L.E., De Stefano, L.A., Schmitt, L.M., Woodruff, N.E., Brown, K.L., Tran, M., Wang, J., Pedapati, E.V., Erickson, C.A., and Sweeney, J.A. (2019). Auditory EEG biomarkers in Fragile X Syndrome: clinical relevance. Front. Integr. Neurosci. *13*, 60. https://doi.org/10.3389/fnint.2019.00060.

42. Ethridge, L.E., White, S.P., Mosconi, M.W., Wang, J., Pedapati, E.V., Erickson, C.A., Byerly, M.J., and Sweeney, J.A. (2017). Neural synchronization deficits linked to cortical hyper-excitability and auditory hypersensitivity in fragile X syndrome. Mol. Autism *8*, 22. https://doi.org/10.1186/s13229-017-0140-1.

43. Wang, J., Ethridge, L.E., Mosconi, M.W., White, S.P., Binder, D.K., Pedapati, E.V., Erickson, C.A., Byerly, M.J., and Sweeney, J.A. (2017). A resting EEG study of neocortical hyperexcitability and altered functional connectivity in fragile X syndrome. J. Neurodev. Disord. *9*, 11. https://doi.org/10.1186/s11689-017-9191-z.
44. Baradits, M., Kakuszi, B., Bálint, S., Fullajtár, M., Mód, L., Bitter, I., and Czobor, P. (2019). Alterations in resting-state gamma activity in patients with schizophrenia: a high-density EEG study. Eur. Arch. Psychiatry Clin. Neurosci. *269*, 429–437. https://doi.org/10.1007/s00406-018-0889-z.
45. Wang, J., Barstein, J., Ethridge, L.E., Mosconi, M.W., Takarae, Y., and Sweeney, J.A. (2013). Resting state EEG abnormalities in autism spectrum disorders. J. Neurodev. Disord. *5*, 24. https://doi.org/10.1186/1866-1955-5-24.
46. Canolty, R.T., and Knight, R.T. (2010). The functional role of cross-frequency coupling. Trends Cogn. Sci. *14*, 506–515. https://doi.org/10.1016/j.tics.2010.09.001.
47. Esghaei, M., Treue, S., and Vidyasagar, T.R. (2022). Dynamic coupling of oscillatory neural activity and its roles in visual attention. Trends Neurosci. *45*, 323–335. https://doi.org/10.1016/j.tins.2022.01.003.
48. Rais, M., Lovelace, J.W., Shuai, X.S., Woodard, W., Bishay, S., Estrada, L., Sharma, A.R., Nguy, A., Kulinich, A., Pirbhoy, P.S., et al. (2022). Functional consequences of postnatal interventions in a mouse model of Fragile X syndrome. Neurobiol. Dis. *162*, 105577. https://doi.org/10.1016/j.nbd.2021.105577.
49. Castrén, M., Pääkkönen, A., Tarkka, I.M., Ryynänen, M., and Partanen, J. (2003). Augmentation of auditory N1 in children with fragile X syndrome. Brain Topogr. *15*, 165–171. https://doi.org/10.1023/a:1022606200636.
50. Ethridge, L.E., White, S.P., Mosconi, M.W., Wang, J., Byerly, M.J., and Sweeney, J.A. (2016). Reduced habituation of auditory evoked potentials indicate cortical hyper-excitability in Fragile X Syndrome. Transl. Psychiatry *6*, e787. https://doi.org/10.1038/tp.2016.48.
51. Dansie, L.E., Phommahaxay, K., Okusanya, A.G., Uwadia, J., Huang, M., Rotschafer, S.E., Razak, K.A., Ethell, D.W., and Ethell, I.M. (2013). Long-lasting effects of minocycline on behavior in young but not adult Fragile X mice. Neuroscience *246*, 186–198. https://doi.org/10.1016/j.neuroscience.2013.04.058.
52. Tranfaglia, M.R. (2011). The psychiatric presentation of fragile x: evolution of the diagnosis and treatment of the psychiatric comorbidities of fragile X syndrome. Dev. Neurosci. *33*, 337–348. https://doi.org/10.1159/000329421.
53. Peier, A.M., McIlwain, K.L., Kenneson, A., Warren, S.T., Paylor, R., and Nelson, D.L. (2000). (Over)correction of FMR1 deficiency with YAC transgenics: behavioral and physical features. Hum. Mol. Genet. *9*, 1145–1159. https://doi.org/10.1093/hmg/9.8.1145.
54. Spencer, C.M., Alekseyenko, O., Serysheva, E., Yuva-Paylor, L.A., and Paylor, R. (2005). Altered anxiety-related and social behaviors in the Fmr1 knockout mouse model of fragile X syndrome. Genes Brain Behav. *4*, 420–430. https://doi.org/10.1111/j.1601-183X.2005.00123.x.
55. Yan, Q.J., Asafo-Adjei, P.K., Arnold, H.M., Brown, R.E., and Bauchwitz, R.P. (2004). A phenotypic and molecular characterization of the fmr1-tm1Cgr fragile X mouse. Genes Brain Behav. *3*, 337–359. https://doi.org/10.1111/j.1601-183X.2004.00087.x.
56. Sidhu, H., Dansie, L.E., Hickmott, P.W., Ethell, D.W., and Ethell, I.M. (2014). Genetic removal of matrix metalloproteinase 9 rescues the symptoms of fragile X syndrome in a mouse model. J. Neurosci. *34*, 9867–9879. https://doi.org/10.1523/JNEUROSCI.1162-14.2014.
57. Schmitt, L.M., Arzuaga, A.L., Dapore, A., Duncan, J., Patel, M., Larson, J.R., Erickson, C.A., Sweeney, J.A., and Ragozzino, M.E. (2023). Parallel learning and cognitive flexibility impairments between Fmr1 knockout mice and individuals with fragile X syndrome. Front. Behav. Neurosci. *16*, 1074682. https://doi.org/10.3389/fnbeh.2022.1074682.
58. Byrne, B.J., Flanigan, K.M., Matesanz, S.E., Finkel, R.S., Waldrop, M.A., D'Ambrosio, E.S., Johnson, N.E., Smith, B.K., Bönnemann, C., Carrig, S., et al. (2025). Current clinical applications of AAV-mediated gene therapy. Mol. Ther. *33*, 2479–2516. https://doi.org/10.1016/j.ymthe.2025.04.045.
59. Yang, C., Tian, Y., Su, F., Wang, Y., Liu, M., Wang, H., Cui, Y., Yuan, P., Li, X., Li, A., et al. (2022). Restoration of FMRP expression in adult V1 neurons rescues visual deficits in a mouse model of fragile X syndrome. Protein Cell *13*, 203–219. https://doi.org/10.1007/s13238-021-00878-z.
60. Razak, K.A., Binder, D.K., and Ethell, I.M. (2021). Neural correlates of auditory hypersensitivity in Fragile X Syndrome. Front. Psychiatry *12*, 720752. https://doi.org/10.3389/fpsyt.2021.720752.
61. Kissinger, S.T., Wu, Q., Quinn, C.J., Anderson, A.K., Pak, A., and Chubykin, A.A. (2020). Visual experience-dependent oscillations and underlying circuit connectivity changes are impaired in Fmr1 KO mice. Cell Rep. *31*, 107486. https://doi.org/10.1016/j.celrep.2020.03.050.
62. Lovelace, J.W., Wen, T.H., Reinhard, S., Hsu, M.S., Sidhu, H., Ethell, I.M., Binder, D.K., and Razak, K.A. (2016). Matrix metalloproteinase-9 deletion rescues auditory evoked potential habituation deficit in a mouse model of Fragile X Syndrome. Neurobiol. Dis. *89*, 126–135. https://doi.org/10.1016/j.nbd.2016.02.002.
63. Cardin, J.A., Carlén, M., Meletis, K., Knoblich, U., Zhang, F., Deisseroth, K., Tsai, L.-H., and Moore, C.I. (2009). Driving fast-spiking cells induces gamma rhythm and controls sensory responses. Nature *459*, 663–667. https://doi.org/10.1038/nature08002.
64. Selby, L., Zhang, C., and Sun, Q.-Q. (2007). Major defects in neocortical GABAergic inhibitory circuits in mice lacking the fragile X mental retardation protein. Neurosci. Lett. *412*, 227–232. https://doi.org/10.1016/j.neulet.2006.11.062.
65. Guyon, N., Zacharias, L.R., Fermino de Oliveira, E., Kim, H., Leite, J.P., Lopes-Aguiar, C., and Carlén, M. (2021). Network asynchrony underlying increased broadband gamma power. J. Neurosci. *41*, 2944–2963. https://doi.org/10.1523/JNEUROSCI.2250-20.2021.
66. Wen, T.H., Afroz, S., Reinhard, S.M., Palacios, A.R., Tapia, K., Binder, D.K., Razak, K.A., and Ethell, I.M. (2018). Genetic reduction of matrix metalloproteinase-9 promotes formation of perineuronal nets around parvalbumin-expressing interneurons and normalizes auditory cortex responses in developing Fmr1 knock-out mice. Cereb. Cortex *28*, 3951–3964. https://doi.org/10.1093/cercor/bhx258.
67. Contractor, A., Ethell, I.M., and Portera-Cailliau, C. (2021). Cortical interneurons in autism. Nat. Neurosci. *24*, 1648–1659. https://doi.org/10.1038/s41593-021-00967-6.
68. Kourdougli, N., Suresh, A., Liu, B., Juarez, P., Lin, A., Chung, D.T., Graven Sams, A., Gandal, M.J., Martínez-Cerdeño, V., Buonomano, D.V., et al. (2023). Improvement of sensory deficits in fragile X mice by increasing cortical interneuron activity after the critical period. Neuron *111*, 2863–2880.e6. https://doi.org/10.1016/j.neuron.2023.06.009.
69. Pirbhoy, P.S., Jonak, C.R., Syed, R., Argueta, D.A., Perez, P.A., Wiley, M.B., Hessamian, K., Lovelace, J.W., Razak, K.A., DiPatrizio, N.V., et al. (2021). Increased 2-arachidonoyl-sn-glycerol levels normalize cortical responses to sound and improve behaviors in Fmr1 KO mice. J. Neurodev. Disord. *13*, 47. https://doi.org/10.1186/s11689-021-09394-x.
70. Lovelace, J.W., Ethell, I.M., Binder, D.K., and Razak, K.A. (2020). Minocycline treatment reverses sound evoked EEG abnormalities in a mouse model of Fragile X Syndrome. Front. Neurosci. *14*, 771. https://doi.org/10.3389/fnins.2020.00771.
71. Holley, A.J., Shedd, A., Boggs, A., Lovelace, J., Erickson, C., Gross, C., Jankovic, M., Razak, K., Huber, K., and Gibson, J.R. (2022). A sound-driven cortical phase-locking change in the Fmr1 KO mouse requires Fmr1 deletion in a subpopulation of brainstem neurons. Neurobiol. Dis. *170*, 105767. https://doi.org/10.1016/j.nbd.2022.105767.
72. Gauthier, D.W., James, N., and Auerbach, B.D. (2025). Altered auditory feature discrimination in a rat model of Fragile X Syndrome. PLoS Biol. *23*, e3003248. https://doi.org/10.1371/journal.pbio.3003248.
73. Croom, K., Rumschlag, J.A., Erickson, M.A., Binder, D.K., and Razak, K.A. (2023). Developmental delays in cortical auditory temporal processing in a mouse model of Fragile X syndrome. J. Neurodev. Disord. *15*, 23. https://doi.org/10.1186/s11689-023-09496-8.
74. Jonak, C.R., Lovelace, J.W., Ethell, I.M., Razak, K.A., and Binder, D.K. (2020). Multielectrode array analysis of EEG biomarkers in a mouse model of Fragile X Syndrome. Neurobiol. Dis. *138*, 104794. https://doi.org/10.1016/j.nbd.2020.104794.
75. Ethridge, L., Thaliath, A., Kraff, J., Nijhawan, K., and Berry-Kravis, E. (2020). Development of neural response to novel sounds in Fragile X Syndrome: potential biomarkers. Am. J. Intellect. Dev. Disabil. *125*, 449–464. https://doi.org/10.1352/1944-7558-125.6.449.

76. Knoth, I.S., Vannasing, P., Major, P., Michaud, J.L., and Lippé, S. (2014). Alterations of visual and auditory evoked potentials in fragile X syndrome. Int. J. Dev. Neurosci. *36*, 90–97. https://doi.org/10.1016/j.ijdevneu.2014.05.003.

77. Bicks, L.K., Koike, H., Akbarian, S., and Morishita, H. (2015). Prefrontal cortex and social cognition in mouse and man. Front. Psychol. *6*, 1805. https://doi.org/10.3389/fpsyg.2015.01805.

78. Bissonette, G.B., and Powell, E.M. (2012). Reversal learning and attentional set-shifting in mice. Neuropharmacology *62*, 1168–1174. https://doi.org/10.1016/j.neuropharm.2011.03.011.

79. Chudasama, Y., and Robbins, T.W. (2006). Functions of frontostriatal systems in cognition: comparative neuropsychopharmacological studies in rats, monkeys and humans. Biol. Psychol. *73*, 19–38. https://doi.org/10.1016/j.biopsycho.2006.01.005.

80. Royall, D.R., Lauterbach, E.C., Cummings, J.L., Reeve, A., Rummans, T.A., Kaufer, D.I., LaFrance Jr, W.C., and Coffey, C.E. (2002). Executive control function: a review of its promise and challenges for clinical research. A report from the Committee on Research of the American Neuropsychiatric Association. J. Neuropsychiatry Clin. Neurosci. *14*, 377–405. https://doi.org/10.1176/jnp.14.4.377.

81. Siegel, J.J., Chitwood, R.A., Ding, J.M., Payne, C., Taylor, W., Gray, R., Zemelman, B.V., and Johnston, D. (2017). Prefrontal cortex dysfunction in Fragile X Mice depends on the continued absence of Fragile X mental retardation protein in the adult brain. J. Neurosci. *37*, 7305–7317. https://doi.org/10.1523/JNEUROSCI.0571-17.2017.

82. Longo, F., Aryal, S., Anastasiades, P.G., Maltese, M., Baimel, C., Albanese, F., Tabor, J., Zhu, J.D., Oliveira, M.M., Gastaldo, D., et al. (2023). Cell-type-specific disruption of cortico-striatal circuitry drives repetitive patterns of behavior in fragile X syndrome model mice. Cell Rep. *42*, 112901. https://doi.org/10.1016/j.celrep.2023.112901.

83. Neuhaus, E., Lowry, S.J., Santhosh, M., Kresse, A., Edwards, L.A., Keller, J., Libsack, E.J., Kang, V.Y., Naples, A., Jack, A., et al. (2021). Resting state EEG in youth with ASD: age, sex, and relation to phenotype. J. Neurodev. Disord. *13*, 33. https://doi.org/10.1186/s11689-021-09390-1.

84. Pedapati, E.V., Schmitt, L.M., Ethridge, L.E., Miyakoshi, M., Sweeney, J.A., Liu, R., Smith, E., Shaffer, R.C., Dominick, K.C., Gilbert, D.L., et al. (2022). Neocortical localization and thalamocortical modulation of neuronal hyperexcitability contribute to Fragile X Syndrome. Commun. Biol. *5*, 442. https://doi.org/10.1038/s42003-022-03395-9.

85. Smith, E.G., Pedapati, E.V., Liu, R., Schmitt, L.M., Dominick, K.C., Shaffer, R.C., Sweeney, J.A., and Erickson, C.A. (2021). Sex differences in resting EEG power in Fragile X Syndrome. J. Psychiatr. Res. *138*, 89–95. https://doi.org/10.1016/j.jpsychires.2021.03.057.

86. Cregenzán-Royo, O., Brun-Gasca, C., and Fornieles-Deu, A. (2022). Behavior Problems and Social Competence in Fragile X Syndrome: A Systematic Review. Genes *13*, 280. https://doi.org/10.3390/genes13020280.

87. Davidson, M., Sebastian, S.A., Benitez, Y., Desai, S., Quinonez, J., Ruxmohan, S., Stein, J.D., and Cueva, W. (2022). Behavioral problems in Fragile X Syndrome: a review of clinical management. Cureus *14*, e21840. https://doi.org/10.7759/cureus.21840.

88. Ding, Q., Sethna, F., and Wang, H. (2014). Behavioral analysis of male and female Fmr1 knockout mice on C57BL/6 background. Behav. Brain Res. *271*, 72–78. https://doi.org/10.1016/j.bbr.2014.05.046.

89. Kazdoba, T.M., Leach, P.T., Silverman, J.L., and Crawley, J.N. (2014). Modeling fragile X syndrome in the Fmr1 knockout mouse. Intractable Rare Dis. Res. *3*, 118–133. https://doi.org/10.5582/irdr.2014.01024.

90. Pietropaolo, S., Guilleminot, A., Martin, B., D'Amato, F.R., and Crusio, W.E. (2011). Genetic-background modulation of core and variable autistic-like symptoms in Fmr1 knock-out mice. PLoS One *6*, e17073. https://doi.org/10.1371/journal.pone.0017073.

91. Gholizadeh, S., Halder, S.K., and Hampson, D.R. (2015). Expression of fragile X mental retardation protein in neurons and glia of the developing and adult mouse brain. Brain Res. *1596*, 22–30. https://doi.org/10.1016/j.brainres.2014.11.023.

92. Dziembowska, M., Pretto, D.I., Janusz, A., Kaczmarek, L., Leigh, M.J., Gabriel, N., Durbin-Johnson, B., Hagerman, R.J., and Tassone, F. (2013). High MMP-9 activity levels in fragile X syndrome are lowered by minocycline. Am. J. Med. Genet. *161*, 1897–1903. https://doi.org/10.1002/ajmg.a.36023.

93. Laroui, A., Galarneau, L., Abolghasemi, A., Benachenhou, S., Plantefève, R., Bouchouirab, F.Z., Lepage, J.F., Corbin, F., and Çaku, A. (2022). Clinical significance of matrix metalloproteinase-9 in Fragile X Syndrome. Sci. Rep. *12*, 15386. https://doi.org/10.1038/s41598-022-19476-y.

94. Afshar, S., Lule, S., Yuan, G., Qu, X., Pan, C., Whalen, M., Brownell, A.-L., and Mody, M. (2022). Longitudinal PET studies of mGluR5 in FXS using an FMR1 knockout mouse model. Transl. Neurosci. *13*, 80–92. https://doi.org/10.1515/tnsci-2022-0217.

95. Dölen, G., and Bear, M.F. (2008). Role for metabotropic glutamate receptor 5 (mGluR5) in the pathogenesis of fragile X syndrome. J. Physiol. *586*, 1503–1508. https://doi.org/10.1113/jphysiol.2008.150722.

96. Osterweil, E.K., Krueger, D.D., Reinhold, K., and Bear, M.F. (2010). Hypersensitivity to mGluR5 and ERK1/2 leads to excessive protein synthesis in the hippocampus of a mouse model of Fragile X Syndrome. J. Neurosci. *30*, 15616–15627. https://doi.org/10.1523/JNEUROSCI.3888-10.2010.

97. Veeraragavan, S., Graham, D., Bui, N., Yuva-Paylor, L.A., Wess, J., and Paylor, R. (2012). Genetic reduction of muscarinic M4 receptor modulates analgesic response and acoustic startle response in a mouse model of fragile X syndrome (FXS). Behav. Brain Res. *228*, 1–8. https://doi.org/10.1016/j.bbr.2011.11.018.

98. Veeraragavan, S., Bui, N., Perkins, J.R., Yuva-Paylor, L.A., and Paylor, R. (2011). The modulation of fragile X behaviors by the muscarinic M4 antagonist, tropicamide. Behav. Neurosci. *125*, 783–790. https://doi.org/10.1037/a0025202.

99. Gocel, J., and Larson, J. (2012). Synaptic NMDA receptor-mediated currents in anterior piriform cortex are reduced in the adult fragile X mouse. Neuroscience *221*, 170–181. https://doi.org/10.1016/j.neuroscience.2012.06.052.

100. Yau, S.-Y., Bettio, L., Chiu, J., Chiu, C., and Christie, B.R. (2019). Fragile-X syndrome is associated with NMDA receptor hypofunction and reduced dendritic complexity in mature dentate granule cells. Front. Mol. Neurosci. *11*, 495. https://doi.org/10.3389/fnmol.2018.00495.

101. Daci, R., and Flotte, T.R. (2024). Delivery of adeno-associated virus vectors to the central nervous system for correction of single gene disorders. Int. J. Mol. Sci. *25*, 1050. https://doi.org/10.3390/ijms25021050.

102. Meyer, K., Ferraiuolo, L., Schmelzer, L., Braun, L., McGovern, V., Likhite, S., Michels, O., Govoni, A., Fitzgerald, J., Morales, P., et al. (2015). Improving single injection CSF delivery of AAV9-mediated gene therapy for SMA: a dose-response study in mice and nonhuman primates. Mol. Ther. *23*, 477–487. https://doi.org/10.1038/mt.2014.210.

103. Cobb, S.R., Hector, R.D., and Ross, P. (2023). Recombinant therapeutic fmr1 constructs and methods of treating fragile x syndrome and related disorders. In International patent number WO2023209364A1, filed April 26, 2023, and granted November 02, 2023.

104. Lovelace, J.W., Rais, M., Palacios, A.R., Shuai, X.S., Bishay, S., Popa, O., Pirbhoy, P.S., Binder, D.K., Nelson, D.L., Ethell, I.M., and Razak, K.A. (2020). Deletion of Fmr1 from forebrain excitatory neurons triggers abnormal cellular, EEG, and behavioral phenotypes in the auditory cortex of a mouse model of Fragile X Syndrome. Cereb. Cortex *30*, 969–988. https://doi.org/10.1093/cercor/bhz141.

105. Wagner, V.A., Thapa, R., Norman, A.O., Varallo, A., Wimberly, J., Rais, M., Gerasymchuk, D., Piepponen, T.P., Razak, K.A., Castren, M.L., and Ethell, I.M. (2026). Astrocytic GABA controls fidelity of temporal cortical processing in Fragile X Syndrome. Neurobiol. Dis. *219*, 107233. https://doi.org/10.1016/j.nbd.2025.107233.

106. Artieda, J., Valencia, M., Alegre, M., Olaziregi, O., Urrestarazu, E., and Iriarte, J. (2004). Potentials evoked by chirp-modulated tones: a new technique to evaluate oscillatory activity in the auditory pathway. Clin. Neurophysiol. *115*, 699–709. https://doi.org/10.1016/j.clinph.2003.10.021.

107. Pérez-Alcázar, M., Nicolás, M.J., Valencia, M., Alegre, M., Iriarte, J., and Artieda, J. (2008). Chirp-evoked potentials in the awake and anesthetized rat. A procedure to assess changes in cortical oscillatory activity. Exp. Neurol. *210*, 144–153. https://doi.org/10.1016/j.expneurol.2007.10.017.

108. Purcell, D.W., John, S.M., Schneider, B.A., and Picton, T.W. (2004). Human temporal auditory acuity as assessed by envelope following responses. J. Acoust. Soc. Am. *116*, 3581–3593. https://doi.org/10.1121/1.1798354.

109. Tallon-Baudry, C., Bertrand, O., Delpuech, C., and Pernier, J. (1996). Stimulus specificity of phase-locked and non-phase-locked 40 Hz visual responses in human. J. Neurosci. *16*, 4240–4249. https://doi.org/10.1523/JNEUROSCI.16-13-04240.1996.

110. Maris, E., and Oostenveld, R. (2007). Nonparametric statistical testing of EEG- and MEG-data. J. Neurosci. Methods *164*, 177–190. https://doi.org/10.1016/j.jneumeth.2007.03.024.

111. Kaidanovich-Beilin, O., Lipina, T., Vukobradovic, I., Roder, J., and Woodgett, J.R. (2011). Assessment of social interaction behaviors. J. Vis. Exp. *2473*. https://doi.org/10.3791/2473.

112. Nguyen, A.Q., Sutley, S., Koeppen, J., Mina, K., Woodruff, S., Hanna, S., Vengala, A., Hickmott, P.W., Obenaus, A., and Ethell, I.M. (2020). Astrocytic ephrin-B1 controls excitatory-inhibitory balance in developing hippocampus. J. Neurosci. *40*, 6854–6871. https://doi.org/10.1523/JNEUROSCI.0413-20.2020.

113. Nygaard, K.R., Maloney, S.E., and Dougherty, J.D. (2019). Erroneous inference based on a lack of preference within one group: Autism, mice, and the social approach task. Autism Res. *12*, 1171–1183. https://doi.org/10.1002/aur.2154.

OMTN, Volume *37*

Supplemental information

Neonatal expression of human FMRP isoform corrects cortical deficits and improves behavior in a mouse model of fragile X syndrome

Anna O. Norman, Courtney Scaramella, Dominik Biezonski, Ralph D. Hector, Alexandra Varallo, Aarushi Sahni, Nadia Farooq, Suzanne R. Burstein, Juliana Benito, Khaleel A. Razak, Jim Selfridge, Stuart Cobb, and Iryna M. Ethell

Supplemental Figures

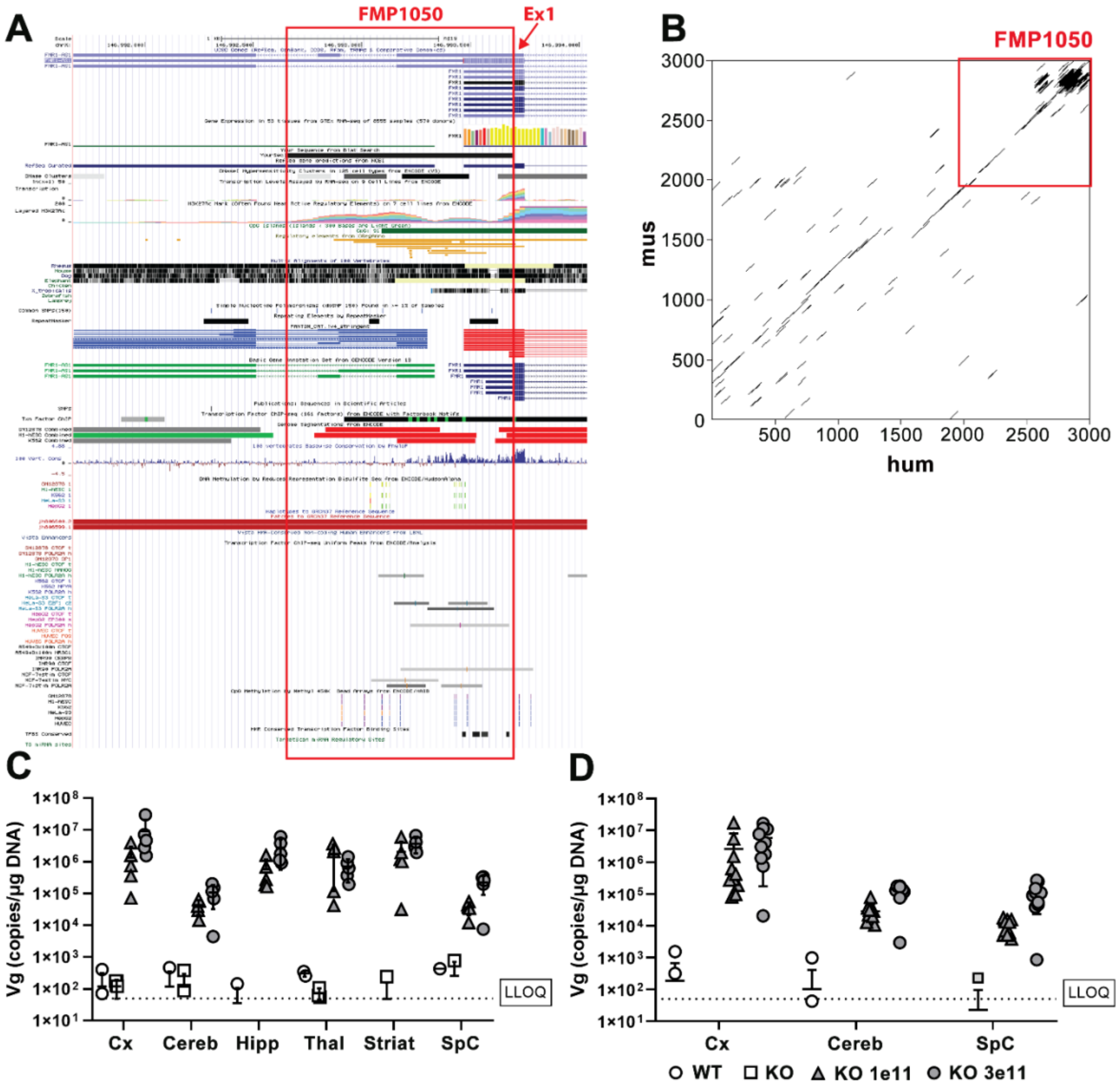

Fig. S1. Computational analysis of the human *FMR1* promoter region and analysis of transduction levels in the tissue of KO mice injected with AAV9-NG276 harvested after EEG and behavioral testing

(A) The human *FMR1* promoter region in the UCSC Genome Browser. The 1050 bp fragment (red box) contains key genetic regulatory elements needed for *FMR1* transgene expression, including CpG island and H3K27Ac marks. (B) Dotplot showing high levels of conservation between human and mouse sequences in the *FMR1* promoter region upstream of the coding exons. Generated by EMBOSS Dotmatcher. (C)

Vector genome (expressed per μg DNA) analyzed in the tissue of WT, KO, and KO injected with AAV9-NG276 at either 1e11 (low-dose) or 3e11 (high-dose) vg/animal (n=5 mice per group) after the EEG recording and (D) behavior testing. The highest dose-dependent levels of transduction were seen across the cortex, and lowest in the cerebellum and spinal cord. Data determined by qPCR analysis of tissues following test article administration. Circles represent individual animals. Error bars are ± SEM. Cx, cortex; Cereb, cerebellum; Hipp, hippocampus; Thal, thalamus; Striat, striatum; SpC, spinal cord; LLOQ, lower limit of quantification.

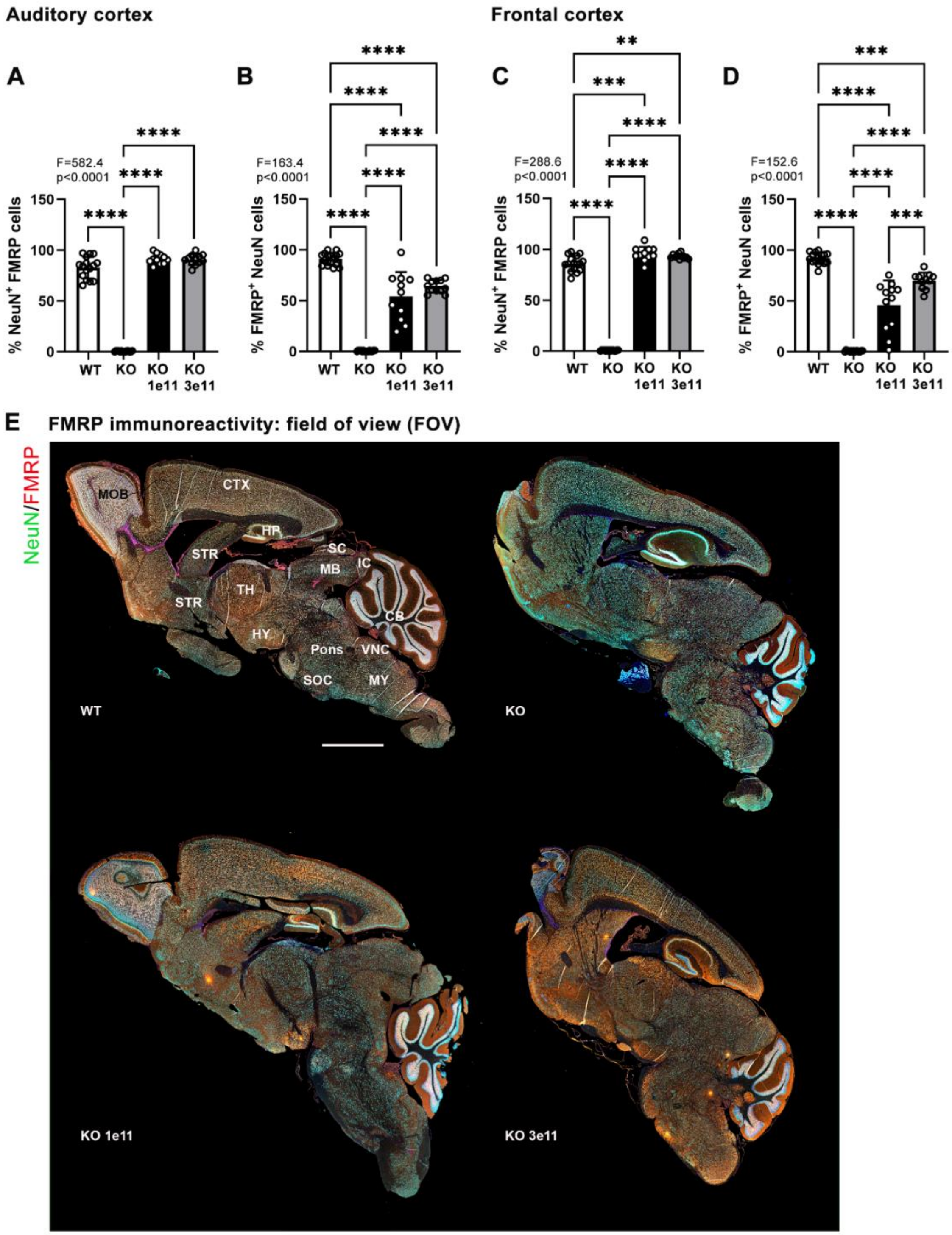

Fig. S2. FMRP levels in the AuC and FC of P28-P30 WT and KO mice ICV injected with vehicle (control) or AAV9-NG276 at a dose of 1e11 vg (1e11) or 3e11 vg (3e11) per mouse.

(A-D) Quantitative analysis of % NeuN+ FMRP cells (A, C) and % FMRP+ NeuN cells (B, D) in the AuC (A-B) and FC (C-D, n=10-15 images from 3-5 mice per group). (E) Representative images showing FMRP (red) and NeuN (green) immunoreactivity in P28-P30 vehicle-injected WT, vehicle-injected KO, and KO injected with AAV9-NG276 at low and high doses, scale bar 2 mm. CB, cerebellum; CTX, cortex; HY, hypothalamus; HP, hippocampus; IC, inferior colliculus; MB, midbrain; MOB, main olfactory bulb; MY, medulla; SC, superior colliculus; STR, striatum; TH, thalamus; VNC, vestibular nuclei. Statistical analysis was done using one-way ANOVA with a Tukey's post-hoc test: *, $p < 0.05$**, $p < 0.01$; ***, $p < 0.001$; ****, $p <0.0001$. All graphs represent average values, and the error bars indicate SD. p values reported in the figure represent ANOVA main effects. FMRP immunoreactivity was significantly increased in the AuC and FC of KO 3e11 group.

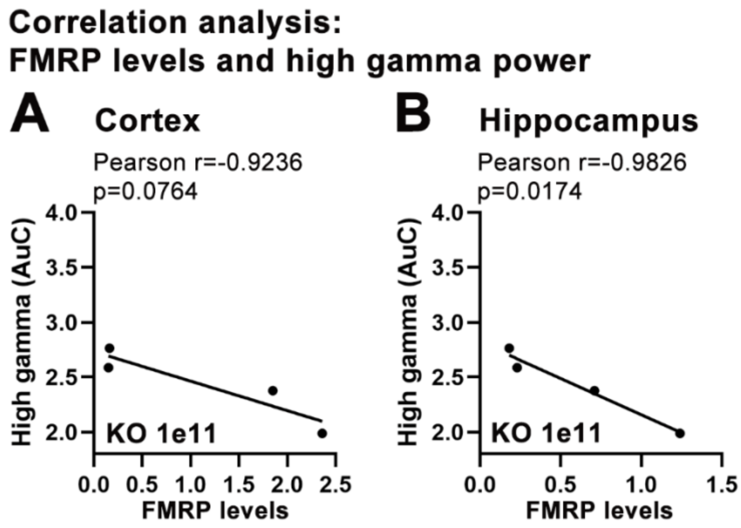

Fig. S3. Correlation between FMRP levels and high gamma power in the AuC of low-dose AAV9-NG276 mice

(A, B) Graphs show correlation between FMRP levels in cortex (A) and hippocampus (B) and high gamma power in AuC of P28-P30 KO injected with AAV9-NG276 at 1e11 (low-dose) vg/animal (n=4 mice per group). Statistical analysis was done using Pearson correlation. Negative correlation was observed between FMRP protein levels and high gamma power in the AuC of low-dose AAV9-NG276 mice.

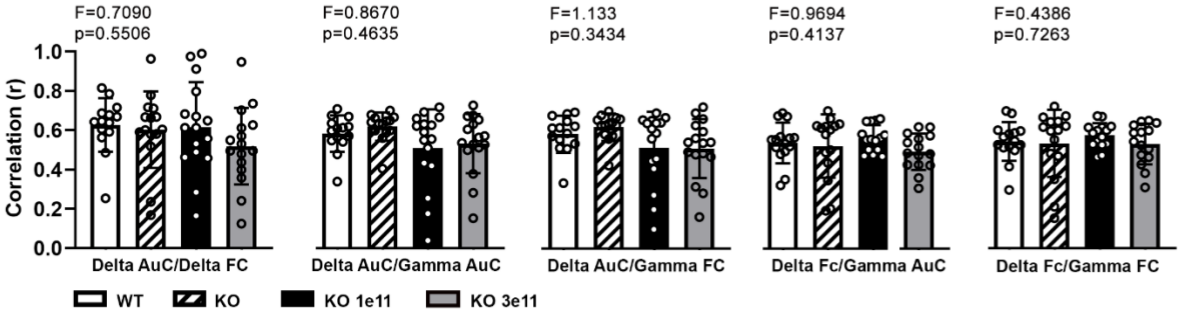

Fig. S4. No difference in delta/high gamma frequency power coupling was detected across the regions in the P28-P30 mice.

Graphs show Pearson's correlation (r) for delta/high gamma frequency power coupling within or across the regions. Values were grouped by frequency bands and analyzed with one-way ANOVA with Tukey's post hoc test. All graphs represent average values, and the error bars indicate SD. p values reported in the figure represent ANOVA main effects. No differences were detected in delta/gamma frequency power coupling across the regions.

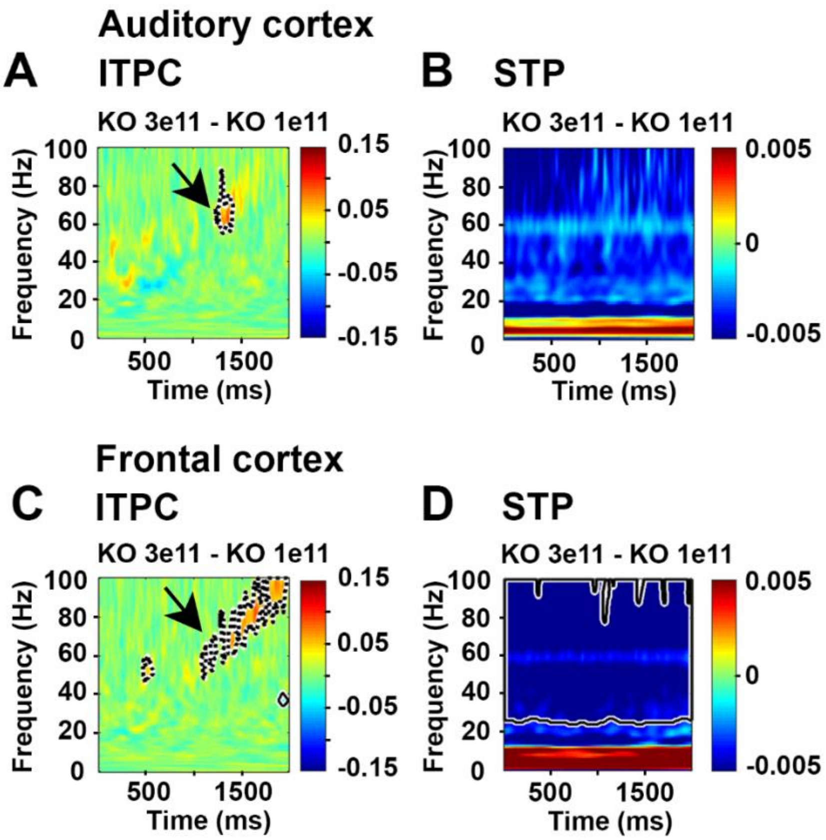

Fig. S5. Phase-locking to "up" chirp stimuli was significantly improved in high-gamma 60-100 Hz range in the P28-P30 AuC and FC of KO 3e11 compared to KO 1e11 mice.

Graphs show comparisons of ITPC (A, C) and STP (B, D) between KO 1e11 (n=16) and KO 3e11 (n=16) in the AuC (A, B) and FC (C, D) of mice. Blue areas indicate a decrease, green indicates no difference, and orange/red indicates an increase. Significant clusters ($p < 0.025$) are highlighted by bold-lined contours (the solid line depicts a significant decrease and the dotted line depicts a significant increase). Phase-locking to "up" chirp stimuli was higher in the AuC and FC of KO 3e11 compared to KO 1e11 mice.

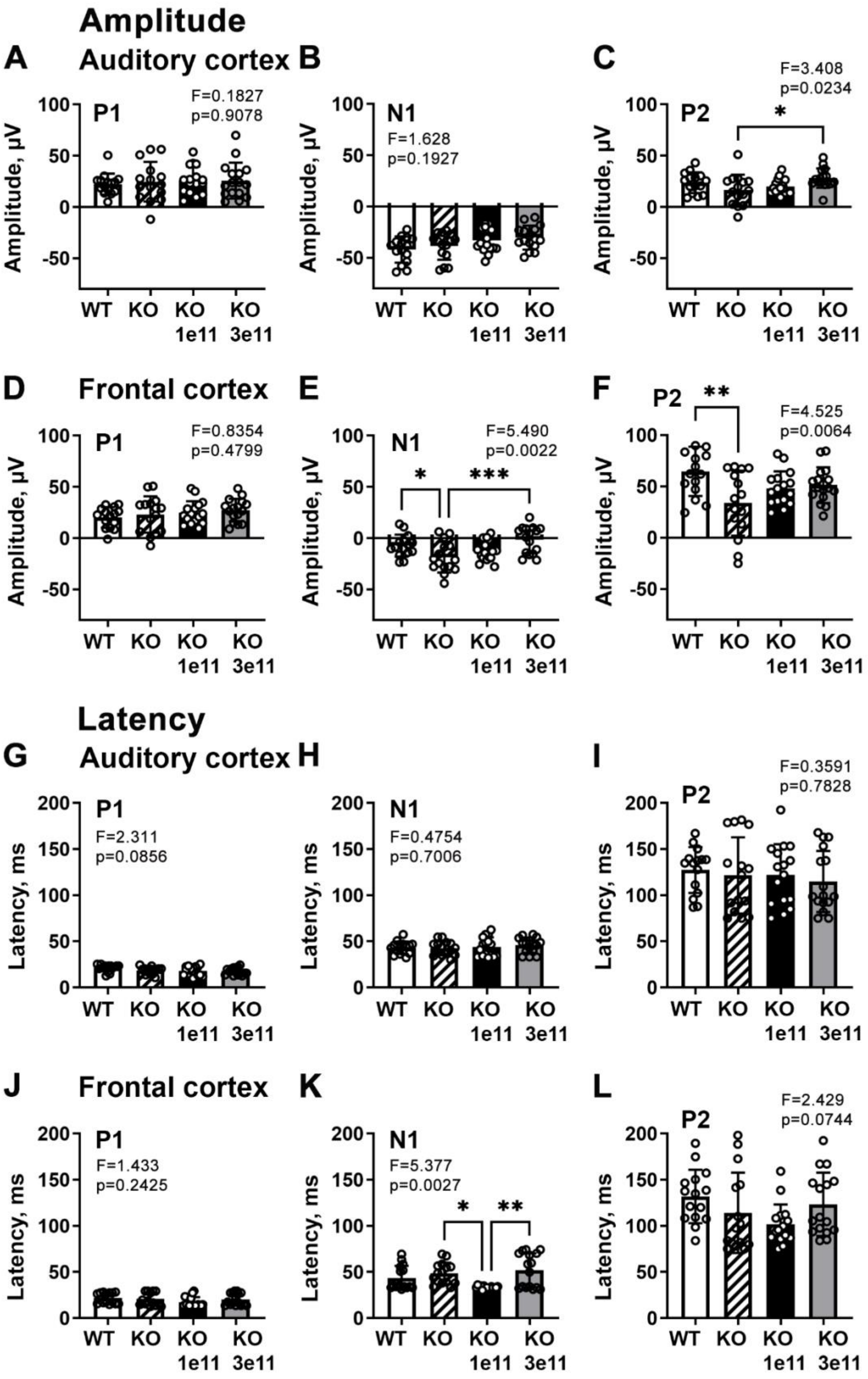

Amplitude
A
Auditory cortex
B
C
P1
F=0.1827
p=0.9078
N1
F=1.628
p=0.1927
P2
F=3.408
p=0.0234
Amplitude, µV
WT
KO
KO 1e11
KO 3e11
D
Frontal cortex
E
F
P1
F=0.8354
p=0.4799
N1
F=5.490
p=0.0022
P2
F=4.525
p=0.0064
Latency
G
Auditory cortex
H
I
P1
F=2.311
p=0.0856
N1
F=0.4754
p=0.7006
P2
F=0.3591
p=0.7828
Latency, ms
J
Frontal cortex
K
L
P1
F=1.433
p=0.2425
N1
F=5.377
p=0.0027
P2
F=2.429
p=0.0744

Fig. S6. N1 amplitude of auditory ERP was enhanced in the FC of P28-30 KO and was significantly reduced to WT levels in KO 3e11 mice.

(A-L) Auditory ERP amplitudes (A-F) and latencies (G-L) in AuC (A-C, G-I) and FC (D-F, J-L) of vehicle-injected WT (n=15), vehicle-injected KO (n=15), and KO injected with AAV9-NG276 at either 1e11 vg (n=16) or 3e11 vg/animal (n=16). Grand average ERPs obtained from P28-P30 mice in response to 100-ms broadband noise presented at 4-Hz repetition rate. P1, N1, and P2 were defined as maximum or minimum voltage deflections within 0–30 ms, 30–80 ms, or 80–150 ms, respectively. Statistical analysis was performed with a one-way ANOVA and Tukey's post hoc test. *$p < 0.05$, ** < 0.01. All graphs represent average values and the error bars indicate SD. p values reported in the figure represent ANOVA main effects. N1 amplitude was elevated in the FC of KO compared to WT mice, and it was normalized to WT levels in KO 3e11 mice.

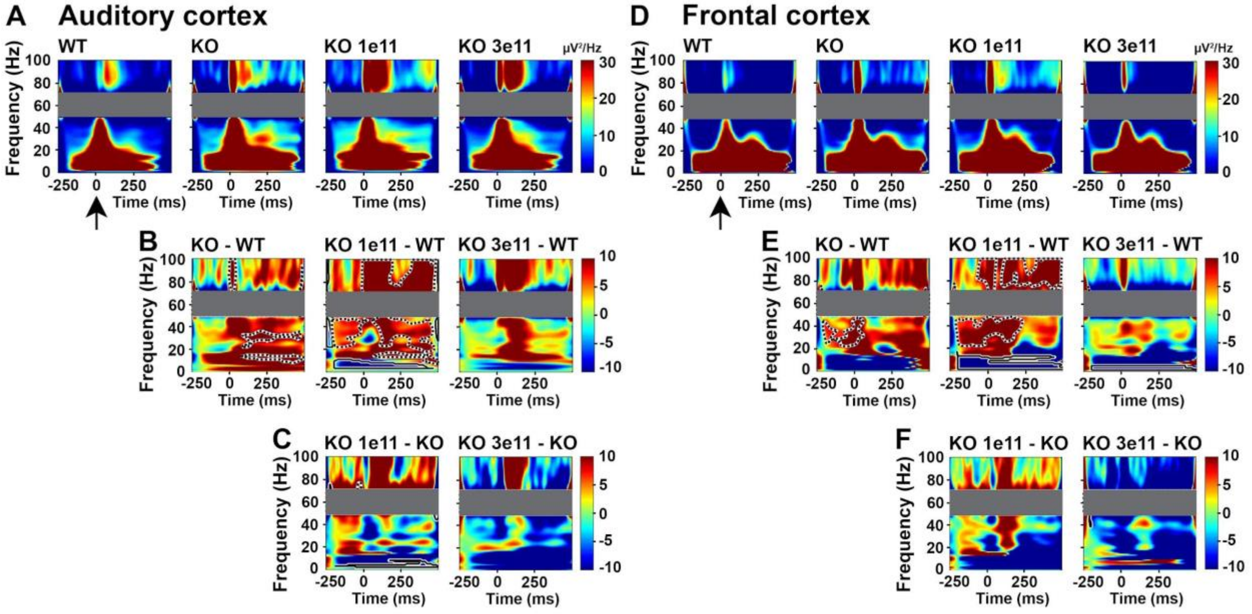

Fig. S7. The power of onset and ongoing responses to sound trains with 0.25 Hz repetition rate was impaired in the P28-P30 AuC and FC of KO mice and was improved in the high-dose group.

(A, D) Single Trial Power (STP) grand average for vehicle-injected WT (n=15), vehicle-injected KO (n=15), and KO injected with AAV9-NG276 at either 1e11 vg (n=16; low-dose) or 3e11 vg/animal (n=16; high dose) in the AuC (A-C) and FC (D-F). (B, C, E, F) Graphs show comparisons to vehicle-injected WT (B, E): KO/WT, low-dose/WT, high-dose/WT; and to vehicle-injected KO (C, F): low-dose/KO and high-dose/KO. Blue areas indicate a decrease, green indicates no difference, and orange/red indicate an increase. Significant clusters ($p < 0.025$) are highlighted by bold-lined contours (the solid line depicts a significant decrease, and the dotted line depicts a significant increase). The gray band indicates signals filtered out at ~60 Hz to prevent electrical line interference. Black arrows indicate signal presentation. The power of onset and ongoing responses to sound trains with 0.25 Hz repetition rate was enhanced in the AuC and FC of KO mice compared to WT mice, and it was improved in the high-dose, but not low-dose group.

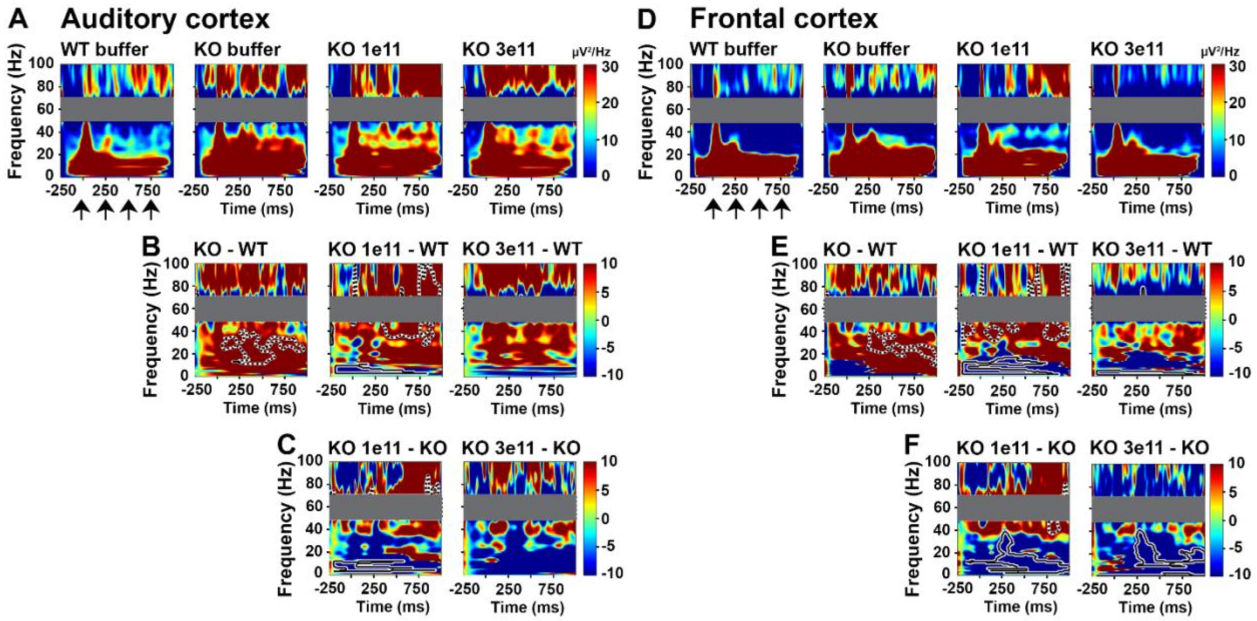

Fig. S8. The power of onset and ongoing responses to sound trains with 4 Hz repetition rate was impaired in the P28-P30 AuC and FC of KO mice and was improved in KO 3e11 but not KO 1e11 mice.

(A, D) Single Trial Power (STP) grand average for vehicle-injected WT (n=15), vehicle-injected KO (n=15), and KO injected with AAV9-NG276 at either 1e11 vg (n=16) or 3e11 vg/animal (n=16) in the AuC (A-C) and FC (D-F). (B, C, E, F) Graphs show comparisons to vehicle-injected WT (B, E): KO/WT, KO 1e11/WT, KO 3e11/WT; and to vehicle-injected KO (C, F): KO 1e11/KO and KO 3e11/KO. Blue areas indicate a decrease, green indicates no difference, and orange/red indicate an increase. Significant clusters ($p < 0.025$) are highlighted by bold-lined contours (the solid line depicts a significant decrease and the dotted line depicts a significant increase). The gray band indicates signals filtered out at ~60 Hz to prevent electrical line interference. Black arrows indicate signal presentation. The power of onset and ongoing responses to sound trains with 0.25 Hz repetition rate was enhanced in the AuC and FC of KO mice compared to WT mice, and it was improved in the KO 3e11 but not KO 1e11 group.

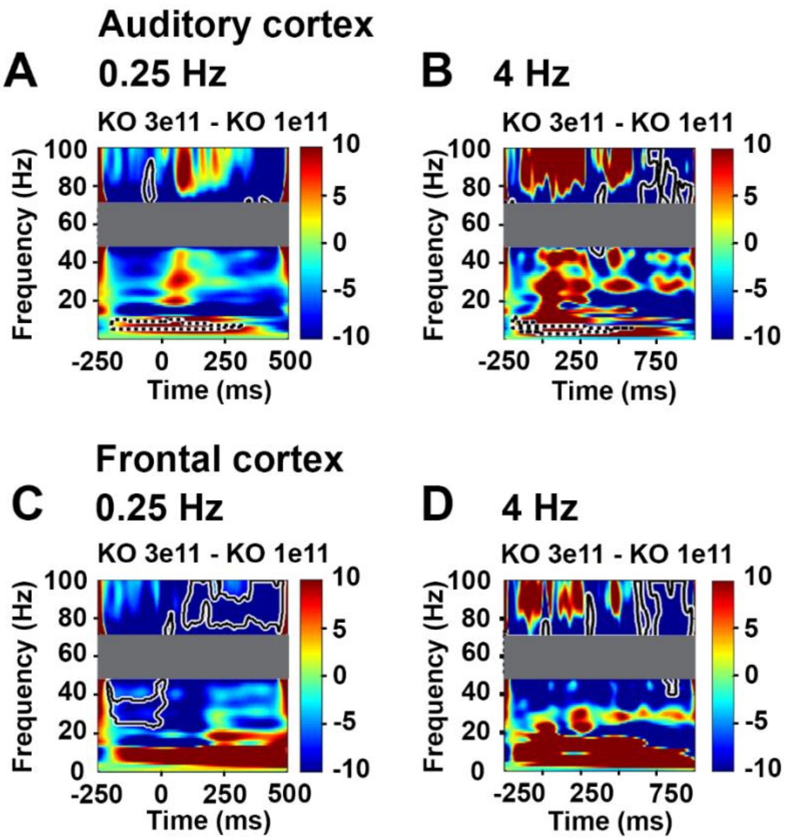

Fig. S9. The power of onset and ongoing responses to sound trains with 0.25 Hz and 4 Hz repetition rate was improved in the P28-P30 AuC and FC of KO 3e11 compared to KO 1e11 mice.

Graphs show comparisons of STP during sound trains with 0.25 Hz (A, C) and 4 Hz (B, D) repetition rate between KO 1e11 (n=16) and KO 3e11 (n=16) in the AuC (A, B) and FC (C, D) of mice. Blue areas indicate a decrease, green indicates no difference, and orange/red indicates an increase. Significant clusters ($p < 0.025$) are highlighted by bold-lined contours (the solid line depicts a significant decrease and the dotted line depicts a significant increase). The gray band indicates signals filtered out at ~60 Hz to prevent electrical line interference. The power of onset and ongoing responses to sound trains with 0.25 Hz and 4 Hz repetition rate was improved in the P28-P30 AuC and FC of KO 3e11 compared to KO 1e11 mice.

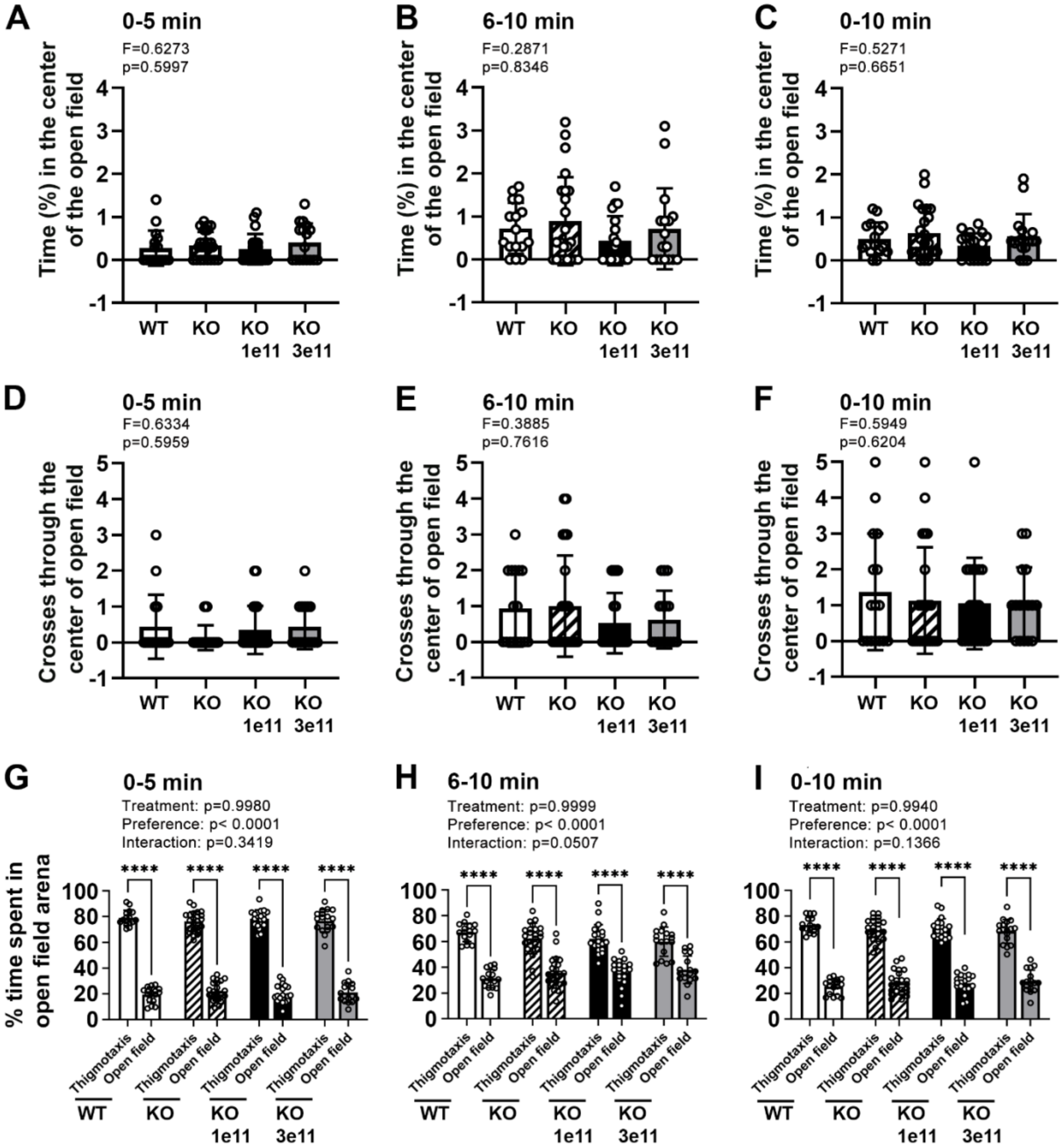

Fig. S10. No difference in exploratory behavior of P28-P30 mice was detected in the open field test.

Graphs demonstrate the performance of vehicle-injected WT (n=16), vehicle-injected KO (n=16), and KO injected with AAV9-NG276 at either 1e11 vg (n=20) or 3e11 vg/animal (n=17) in the open field test at P28-P30. Exploratory behaviors were evaluated by analyzing the % time spent in the center of the open field (A-C), crosses through the center of open field (D-F) and % time spent in thigmotaxis and open field (G-I) during first 5 min (A, D, G), second 5 min (B, E, H) and overall 10 min of testing (C, F, I). Statistical analysis was performed with a one-way (A-F) or two-way (G-I) ANOVA followed by Tukey's post hoc test.

****$p < 0.0001$. All graphs represent average values, and the error bars indicate SD. p values reported in the figure represent ANOVA main effects. No genotype and treatment difference in exploratory behavior was detected in P28-P30 mice.

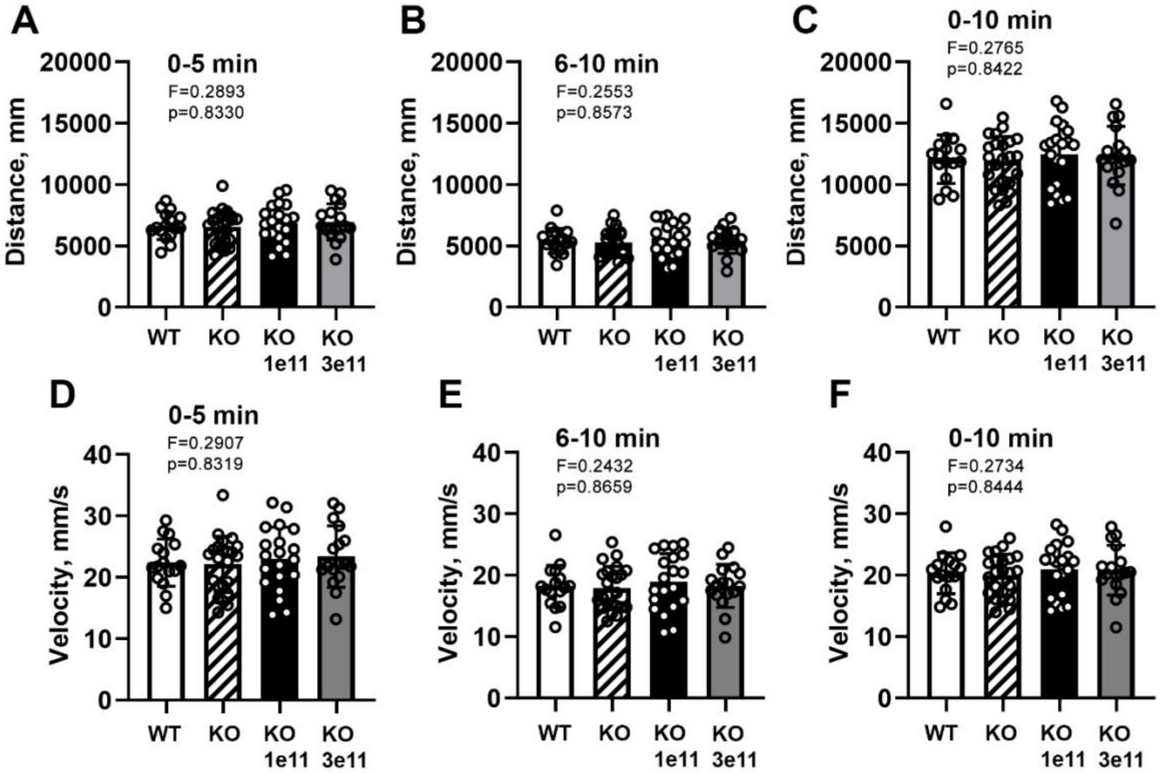

Fig. S11. No difference in locomotor activity of P28-P30 mice was detected in the open field test.

Graphs demonstrate the performance of vehicle-injected WT (n=16), vehicle-injected KO (n=16), and KO injected with AAV9-NG276 at either 1e11 vg (n=20) or 3e11 vg/animal (n=17) in the open field test at P28-P30. Locomotor activity was evaluated by analyzing overall distance traveled (A-C) and velocity (D-F) in of the open field during first 5 min (A, D), second 5 min (B, E) and overall 10 min of testing (C, F). Statistical analysis was performed with a one-way ANOVA followed by Tukey's post hoc test. All graphs represent average values, and the error bars indicate SD. p values reported in the figure represent ANOVA main effects. No genotype and treatment difference in locomotor activity was detected in P28-P30 mice.

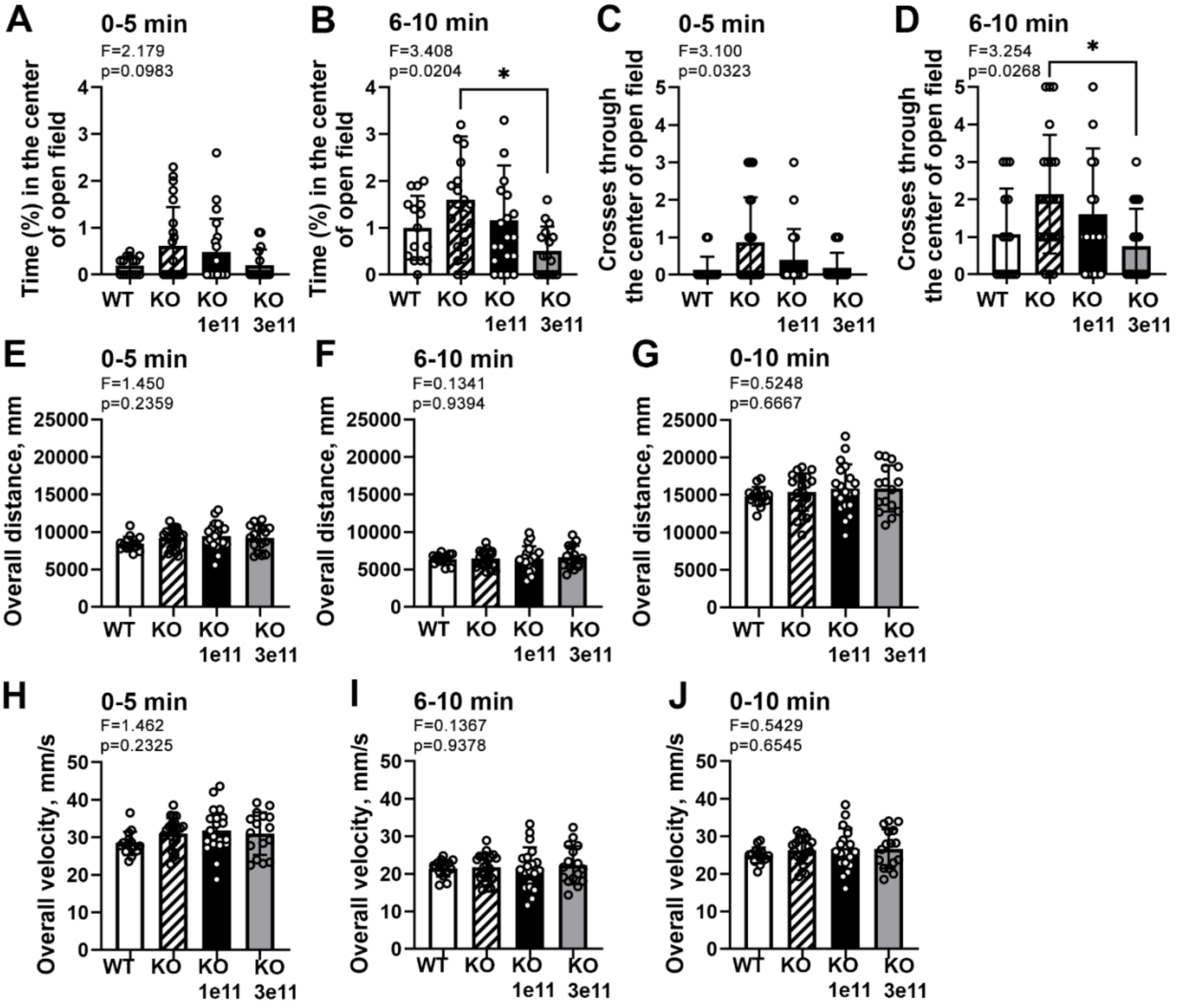

Fig. S12. No difference in locomotor activity of P60-P65 mice was detected in the open field test.

Graphs demonstrate the performance of vehicle-injected WT (n=16), vehicle-injected KO (n=16), and KO injected with AAV9-NG276 at either 1e11 vg (n=20) or 3e11 vg/animal (n=17) in the open field test at P60-P65. Exploratory behaviors were evaluated by analyzing the % time spent in the center of the open field (A, B), crosses through the center of open field (C-D) and locomotor activity was evaluated by analyzing distance (E-G) and velocity (H-J) during first 5 min (A, C, E, H), second 5 min (B, D, F, I) and overall 10 min of testing (G, J). Statistical analysis was performed with a one-way (A-F) or two-way (G-I) ANOVA followed by Tukey's post hoc test. *$p < 0.05$. All graphs represent average values, and the error bars indicate SD. p values reported in the figure represent ANOVA main effects. No genotype and treatment difference in locomotor activity was detected in P60-P65 mice.

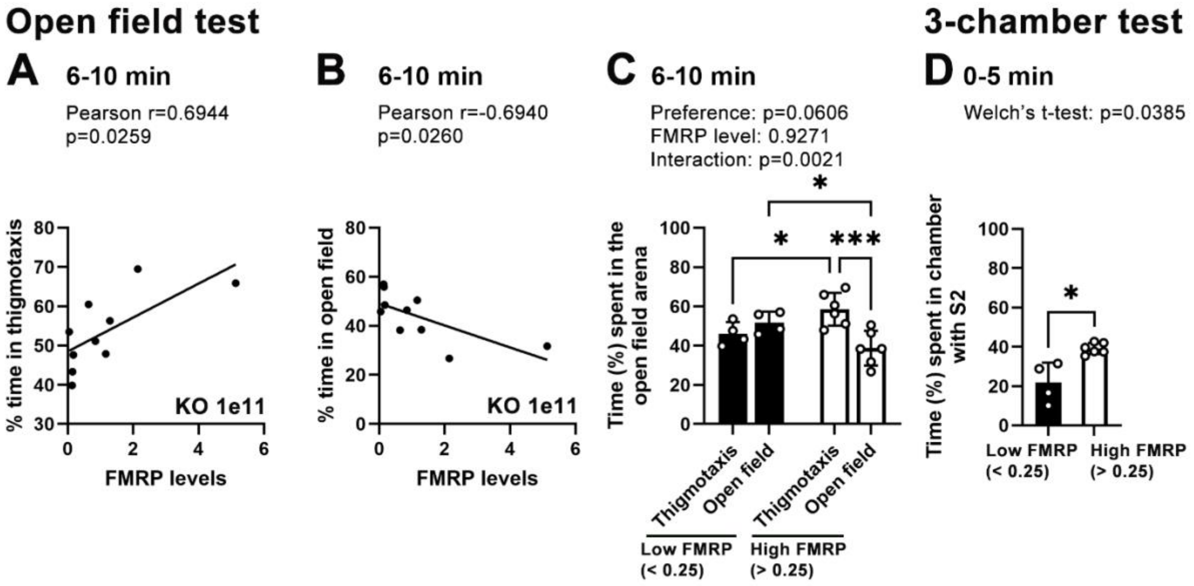

Fig. S13. Correlation between FMRP levels in cortex and exploratory behaviors of P60-P65 low-dose AAV9-NG276 mice

(A-C) Graphs show correlation between FMRP levels in cortex and exploratory behaviors, including % time spent in thigmotaxis (A), open field (B) and location preference based on FMRP levels (C) of P60-P65 KO injected with AAV9-NG276 at 1e11 (low-dose) vg/animal (n=10 mice per group). (D) Graph shows social novelty preference based on FMRP levels (low < 0.25 or high >0.25 FMRP compared to WT levels) in the cortex of low-dose KO mice. Statistical analysis was done using Pearson's correlation (A, B), two-way ANOVA followed by Fisher's LSD post-hoc test (C) or Welch's t-test (D). *$p < 0.05$, ***$p < 0.001$. Data represent mean ± standard deviation (SD). Negative correlation was observed between FMRP protein levels in cortex and exploratory behaviors of low-dose AAV9-NG276 mice.

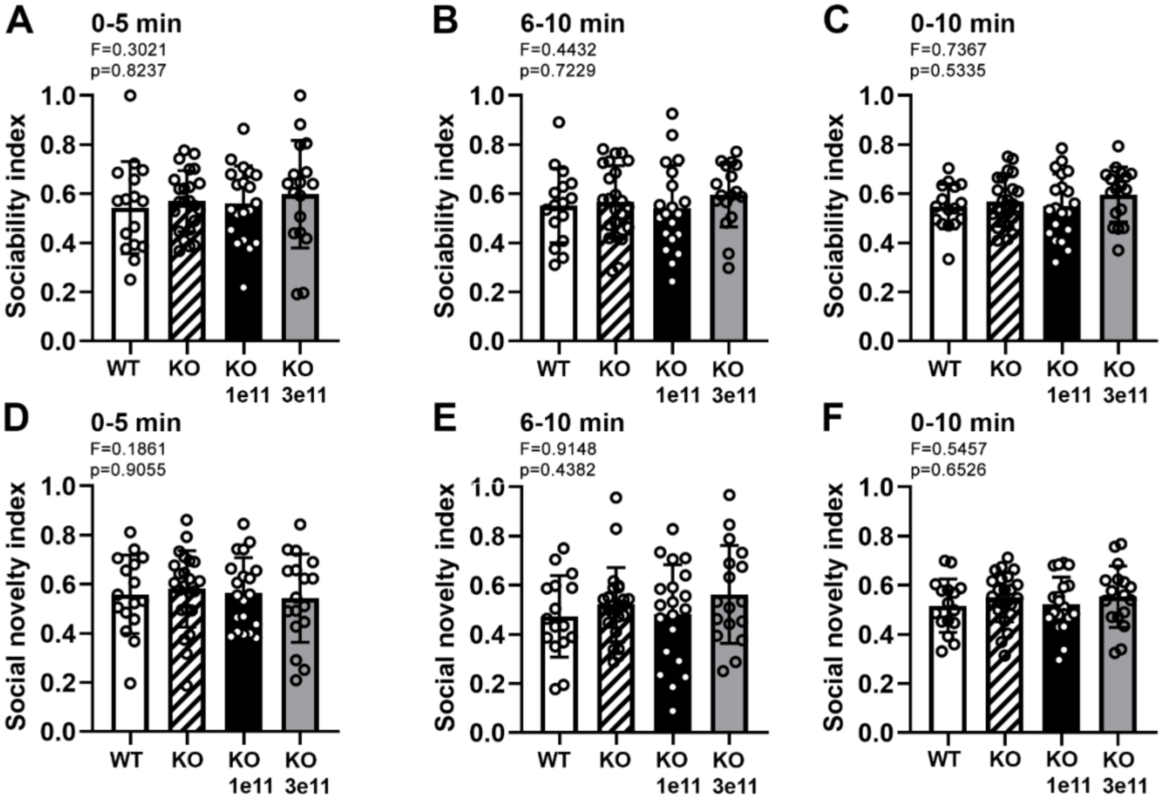

Fig. S14. No difference in sociability index of social preference index of P28-P30 mice was detected in the 3-chamber test.

Graphs demonstrate performance of P28-P30 vehicle-injected WT (n=16), vehicle-injected KO (n=16), and KO injected with AAV9-NG276 at either 1e11 vg (n=20) or 3e11 vg/animal (n=17) in the 3-chamber test (A-F). Sociability index (A-C) was evaluated by measuring the time spent with Stranger 1 mouse compared to the empty cage during session 1. Social novelty index (D-F) was evaluated by assessing the time spent with Stranger 2 mouse compared to the now familiar S1 mouse during session 2. For the sociability index, > 0.5 indicates more time spent in the chamber containing Stranger 1. For the social novelty preference index, > 0.5 indicates more time spent in the chamber containing Stranger 2 or a new stranger mouse. Analysis of first 5 min (A, D), second 5 min (B, E) and overall 10 min (C, F) of testing was done. Statistical analysis was performed with a one-way ANOVA followed by Fisher's LSD post-hoc test. All graphs represent average values and the error bars indicate SD. p values reported in the figure represent ANOVA main effects. No difference in sociability index of social preference index of P28-P30 mice was detected in the 3-chamber test.

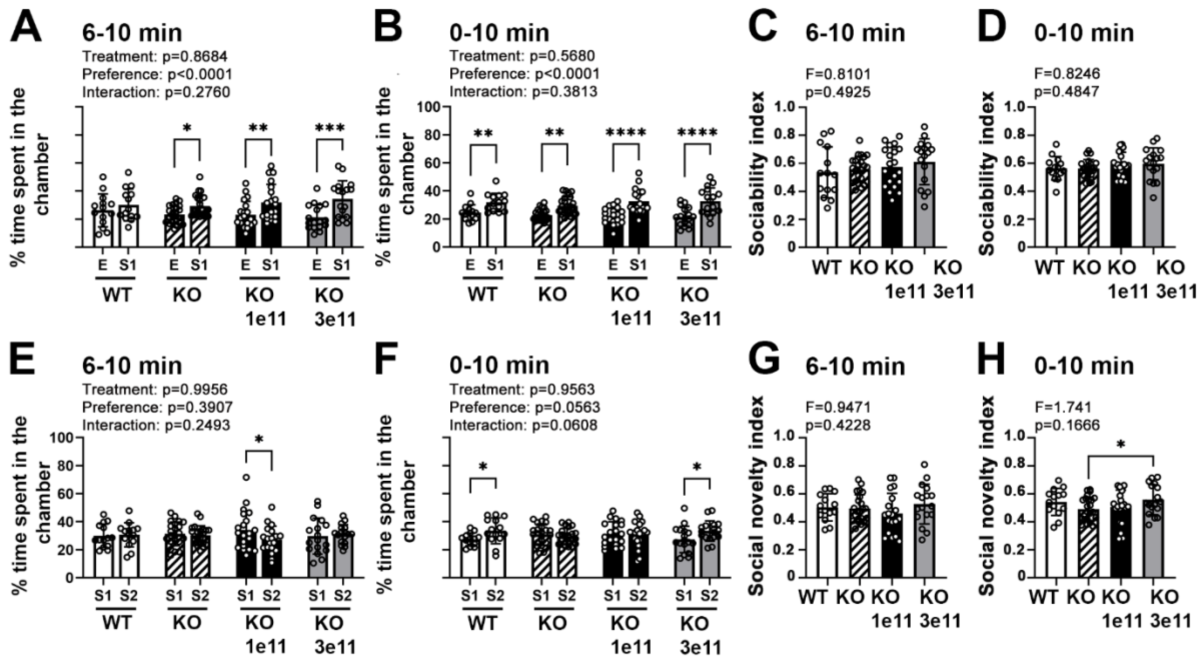

Fig. S15. No difference in sociability and social preference was detected in adult mice during second 5 min of testing.

Graphs show sociability (A, B), sociability index (C, D; evaluated by measuring the time spent with Stranger 1 mouse (S1) compared to the empty cage (E)), social novelty preference (E, F) and social novelty index (G, H; evaluated by assessing the time spent with Stranger 2 mouse (S2) compared to the now familiar S1 mouse (S1)) during second 5 min (A, C, E, G) and overall 10 min (B, D, F, H) in vehicle-injected WT (n=16), vehicle-injected KO (n=16), and KO injected with AAV9-NG276 at either 1e11 vg (n=20) or 3e11 vg/animal (n=17). For the sociability index, > 0.5 indicates more time spent in the chamber containing Stranger 1. For the social novelty preference index, > 0.5 indicates more time spent in the chamber containing Stranger 2 or a new stranger mouse. Statistical analysis was performed with a one-way (C, D, G, H) or two-way (A, B, E, F) ANOVA followed by Fisher's LSD post-hoc test. *p < 0.05, **p < 0.01, ***p < 0.001, ****p < 0.0001. All graphs represent average values and the error bars indicate SD. p values reported in the figure represent ANOVA main effects. No difference in sociability and social preference was detected in adult mice during second 5 min or overall 10 min of testing.

Supplemental tables

Table S1_Statistics for Figure 1

Table S2_Statistics for Figure S2

Table S3_Statistics for Figure 2

Table S4_Statistics for Figure S3

Table S5_Statistics for Figure S4

Table S6_Statistics for Figure 5

Table S7_Statistics for Figure S6

Table S8_Statistics for Figure S10

Table S9_Statistics for Figure S11

Table S10_Statistics for Figure 6

Table S11_Statistics for Figure S12

Table S12_Statistics for Figure S13

Table S13_Statistics for Figure S14

Table S14_Statistics for Figure 7

Table S15_Statistics for Figure S15
